# Adherence to Actigraphic Devices in Elementary School–Aged Children: Systematic Review and Meta-Analysis

**DOI:** 10.2196/79718

**Published:** 2025-11-03

**Authors:** Anna C Morris, Asilay Seker, Laurence Telesia, Alice Wickersham, Brian CF Ching, Rahul Roy, Sophie Epstein, Faith Matcham, Edmund Sonuga-Barke, Johnny Downs

**Affiliations:** 1 CAMHS Digital Lab, King’s Maudsley Partnership, IOPPN, King's College London and South London and Maudsley NHS Foundation Trust, 16 De Crespigny Park, London, SE5 8AB, United Kingdom; 2 Department of Child and Adolescent Psychiatry, IOPPN, King's College London, London, United Kingdom; 3 School of Psychology, University of Sussex, Brighton and Hove, United Kingdom; 4 Maudsley Biomedical Research Centre, IOPPN, King's College London and South London and Maudsley NHS Foundation Trust, London, United Kingdom

**Keywords:** actigraphy, adherence, children, ambulatory monitoring, fitness trackers, telemedicine, sleep, motor activity

## Abstract

**Background:**

Consistent wear is essential for valid and reliable actigraphy data. Adherence to actigraphy may be challenging in primary school children due to developmental and design considerations, yet no quantitative synthesis of adherence in this age group exists.

**Objective:**

The aim of this study was to provide the first pooled estimate of actigraphy adherence in primary school–aged children and examine the impact of individual, device, and study-specific factors on adherence.

**Methods:**

We searched seven electronic databases for studies reporting adherence to actigraphy in primary school–aged children. Searches were conducted in Embase, MEDLINE, PsycINFO, Social Policy and Practice via OVID, Education Resources Information Center, British Education Index, and CINAHL via EBSCO using database-specific search strategies conducted between January 2018 and January 24, 2023. Forward and backward citation searches were completed on the Web of Science Core Collection and Google Scholar. Gray literature searches were undertaken in PsycEXTRA and Healthcare Management Information Consortium. Empirical studies reporting quantitative data on adherence to community-based actigraphy in children aged 5‐11 years (or if ≥50% of the average age fell within this range) were included. Eligible studies were written in English and could be published or unpublished. Risk of bias was assessed using an 8-item checklist adapted from Berger et al’s actigraphy reporting standards. All included studies were narratively synthesized, and adherence data were pooled in a proportional meta-analysis. Adherence was calculated as the proportion of children meeting wear-time criteria to be included in the analysis compared to the number of children invited to use the device at baseline. Meta-regression was used to examine the impact of individual, device, and study-specific factors on adherence. Prediction intervals were calculated to estimate the range of adherence expected across future studies.

**Results:**

Data were extracted from 235 studies (N=148,161); of these, 135 studies (n=64,541) provided adherence data for proportional meta-analysis. Pooled adherence, measured across 1‐140 days, was 81.6% (95% CI 78.7%‐84.4%; *I*^2^=98.8%). The prediction intervals (42.8%-100%) indicated substantial variability in adherence estimates across studies. Meta-regression suggested that individual characteristics contributed to observed heterogeneity as children with a physical health diagnosis (b=0.236, 95% CI 0.009-0.464; *P*=.04) and those with neurodevelopmental or mental health diagnosis (b=0.395, 95% CI 0.125-0.665; *P*=.004) demonstrated higher adherence than undiagnosed children, though these effects were of modest magnitude. No significant effects were found for age, placement, protocol length, protocol deviation, or incentivization. Reporting quality was poor, with only 3.4% of studies satisfying all criteria.

**Conclusions:**

This review demonstrates generally high actigraphy adherence in primary school–aged children, particularly those with health conditions. However, observed variability indicates that adherence was much lower in some contexts, underscoring that the reported pooled adherence cannot be assumed across future actigraphy applications within this age group. Future research should use standardized adherence reporting and should plan for adherence variability.

## Introduction

Wearable activity trackers, especially actigraphs, are unparalleled in their ability to provide naturalistic and cost-effective motion data, making them invaluable for monitoring children’s physical activity and sleep patterns [1,2]. Actigraphy is now widely deployed across research settings, including clinical trials, and is increasingly being integrated into health care settings to obtain objective biomarkers for pediatric activity and sleep [3-8]. However, despite its growing use, several methodological barriers to collecting actigraphic data from children persist, including algorithm variability, monitoring protocols, device placement, and reporting standards [9-12].

Adequate adherence is required to collect valid and reliable data from these devices [13,14]. Inconsistent use or nonadherence can impact data quality and diminish the generalizability of findings [5,15]. What constitutes adequate wear duration varies, with a review identifying eight distinct timeframes, most commonly requiring three to four valid days of wear among youth [3]. This threshold is likely changing as interest in longer-term passive monitoring grows, particularly in health contexts where actigraphy can assess the ongoing treatment efficacy, symptom variation, and broader behavioral patterns over time [16-19].

Assessing adherence is therefore essential to understanding the acceptability and feasibility of actigraphy monitoring in children [7]. Unlike adults, children have distinct physical and behavioral requirements that typically necessitate modifications to products originally developed for adults to ensure accurate physiological measurements and to prevent adverse developmental and psychosocial consequences, such as fear of bullying or appearing different [7,20-22]. Though child-oriented wearables exist, most actigraphy studies continue to rely on general-purpose devices used across all age groups, with only small adjustments recommended for pediatric protocols [7,19,23].  

Qualitative research suggests children generally prefer actigraphs that are functional, visually appealing, and easy to use [24], whereas device or placement-related features including discomfort, charging difficulties, bulkiness, difficulties in engagement due to lack of visual information, and forgetting to reattach devices are often cited as reasons for reduced adherence [25-27]. Yet these subjective insights are mostly unsupported by robust behavioral data on children’s engagement or how individual, study, and device-related factors may affect adherence. Previous research on determinants of adherence in this group attempted to quantify the influence of a few factors such as age, device placement site, or incentivization. However, this limited evidence mainly comes from small-scale studies, which report contradictory findings [15,28-31].

To date, no attempt has been made to quantify adherence data from studies of actigraphy in children. Adherence information is often available within attrition metrics, and systematic synthesis of these data may offer valuable insight into patterns of engagement across populations, protocols, and devices. Accordingly, the primary aim of this systematic review and meta-analysis is to pool the overall rate of children’s adherence to actigraphs. The secondary aim is to assess how individual, device, and study-specific factors, including age, health status, placement, protocol length, deviation from protocol length, study purpose, and incentivization, influence adherence. This review is timely, as researchers or clinicians intending to use actigraphy with children lack clear guidance on protocol development, given the paucity of data on how long children will wear these devices.

## Methods

### Reporting Guidelines and Protocol Registration

The PRISMA (Preferred Reporting Items for Systematic Reviews and Meta-Analyses) [32], the PRISMA-S extension for reporting literature searches [33], and MOOSE (Meta-Analyses of Observational Studies in Epidemiology) [34] were followed for conducting this review and meta-analysis, and for reporting its findings (Checklists1  2). The review protocol was registered in PROSPERO (CRD42021232466) and subsequently peer-reviewed and published in BMJ Open, where the search strategy, eligibility for study inclusion, and data extraction details have been described [4]. Protocol changes are detailed in Multimedia Appendix 1.

### Search Strategy and Data Management

The electronic databases Embase, MEDLINE, PsycINFO, Social Policy and Practice through the OVID interface, Education Resources Information Center (ERIC), British Education Index, and CINAHL through the EBSCO interface were searched using database-specific search strategies. All search strategies (Multimedia Appendix 2) were developed and carried out by ACM, with assistance from a librarian who reviewed search strategies to ensure completeness and accuracy. All database searches were conducted between January 2018 and January 24, 2023, and no updated searches were conducted following this date. Search outputs (see Multimedia Appendix 2 for the number of articles retrieved for each database) were imported into EndNote (version 20), where duplicates were automatically detected by the software and manually confirmed and removed by ACM. Title and abstract screening were then conducted within EndNote. Full-text screening and data extraction were completed in Microsoft Excel. For all citations included in the review, both forward and backward citation searching were conducted on Web of Science Core Collection and Google Scholar. Gray literature searches were conducted within PsycEXTRA, a complementary database to PsycINFO and the Healthcare Management Information Consortium, comprising records from the Department of Health and the King’s Fund.

### Eligibility Criteria

Empirical research, including feasibility, pilot, observational cohort, or intervention studies, reporting on adherence to actigraph use by children aged 5‐11 years in a community setting was included in this review. No restrictions were placed on the purpose of actigraph use. Studies that involved children outside of the age range of interest were included where at least 50% of participants met the age criteria or if the reported mean, median, or IQR fell within the 5‐ to 11-year age range. Adherence, informed by the Theoretical Framework of Acceptability, is defined as a quantitative marker of acceptability (ie, behavioral acceptability, especially attrition rates) [35]. To be included, studies had to be written in English, owing to translation resource constraints, and could be published or unpublished.

### Data Extraction

Extracted data included individual characteristics (age, sex, health status, ethnicity), device characteristics (brand and model, purpose, placement, protocol length [the number of days children were instructed to wear the actigraphic device], and analysis wear time [number of valid days required to be included in the analytical sample]). Study-level data were also collected, including title, year of publication, author, journal or source, study location, design, and incentivization, as well as the number of participants invited and those who adhered to actigraph wear. For meta-analysis, the main outcome was the pooled adherence rate. In cases where adherence was not explicitly reported in the study publication, it was manually calculated as the percentage of children who provided valid data that met the analytic sample inclusion criteria relative to the total number of children invited to use an actigraph at baseline. Attrition due to device errors (eg, malfunction, failure to initialize, or data loss) was not categorized as nonadherence; only nonwearing by children was classified as nonadherence.

### Study Selection

Title and abstract screening were completed by two independent reviewers (ACM and RR) with 20% of retrieved records double screened, achieving an agreement of 0.89 using Cohen κ. Disagreements were resolved with the input of a third reviewer (JD). Three reviewers completed the full-text review with 20% of records entering this phase double-screened, achieving perfect agreement (ACM, RR, and LT).

### Data Extraction

Data extraction was completed by two independent reviewers (ACM and RR) with 12% of included studies double extracted. A third reviewer (AS) also checked all the data extracted for meta-analysis for validation purposes, with discrepancies resolved through discussion until consensus was achieved.

### Quality Assessment

As there were no available quality or bias assessment tools for publications reporting wearable adherence, Berger et al’s recommendations for use of actigraphic devices in research were modified to develop a customized checklist for evaluating reporting standards [36]. Two independent reviewers scored the checklist on a binary scale (0/1) based on eight domains of reporting quality (Multimedia Appendix 3). Briefly, these included whether studies reported on devices (1) type that is, brand or model, (2) placement, (3) allocation or collection, (4) usage instructions, (5) contextual data that is, wear diary, (6) protocol length, (7) analysis wear time, and (8) reporting data that is, reasons for missing or unusable data. Studies with a sum score of 8 were classified as high quality and all else as low quality.

### Data Synthesis and Analysis

We narratively synthesized relevant findings of all included studies. Studies that included discernible adherence rates were included in a random effects meta-analysis, in which the main outcome was pooled adherence, presented with a corresponding forest plot. This approach was chosen based on expected heterogeneity in adherence between different populations, actigraphs, and study characteristics. Between-study variance (τ^2^) was computed using the restricted maximum likelihood method, which is recommended in the presence of high heterogeneity [37]. Similarly, confidence intervals were computed using the Hartung–Knapp–Sidik–Jonkman method, which incorporates uncertainty in τ^2^ to produce more accurate estimates when heterogeneity is high [38].

To be eligible for meta-analysis studies, studies had to have investigated the acceptability of a single actigraph only, reported either a single adherence metric from the first time point the children were measured or both a protocol length and analysis wear time for manual computation of the adherence metric (if the same children are measured at different time points, only studies that included discernible adherence data from the first time they were measured were eligible), and included adequately discernible adherence data from the age group of interest (5‐ to 11-y olds).

Stata V.18 (StataCorp LLC) was used for all statistical analyses. Statistical significance was set at *α*=.05, with 95% CIs reported for all estimates. Associations between adherence rate and age group, diagnosis, device placement, protocol length, protocol deviation (the difference between protocol and analysis wear times, divided by protocol length, multiplied by 100 to convert it to a percentage), wear purpose, and incentivization were examined via univariate meta-regression models, which are also visually presented. *I*^2^ and τ^2^ were used to assess heterogeneity, and prediction intervals were computed to estimate anticipated adherence rates in future actigraph applications. Small-study effects, which may be indicative of publication bias or other causes of asymmetry, were assessed using a Doi plot and Luis Furuyama-Kanamori index (LFK). The presence of asymmetry in the Doi plot and an LFK index outside the range of (–1, +1) were deemed indicators of small-study effects [39,40].

## Results

**Figure 1.**
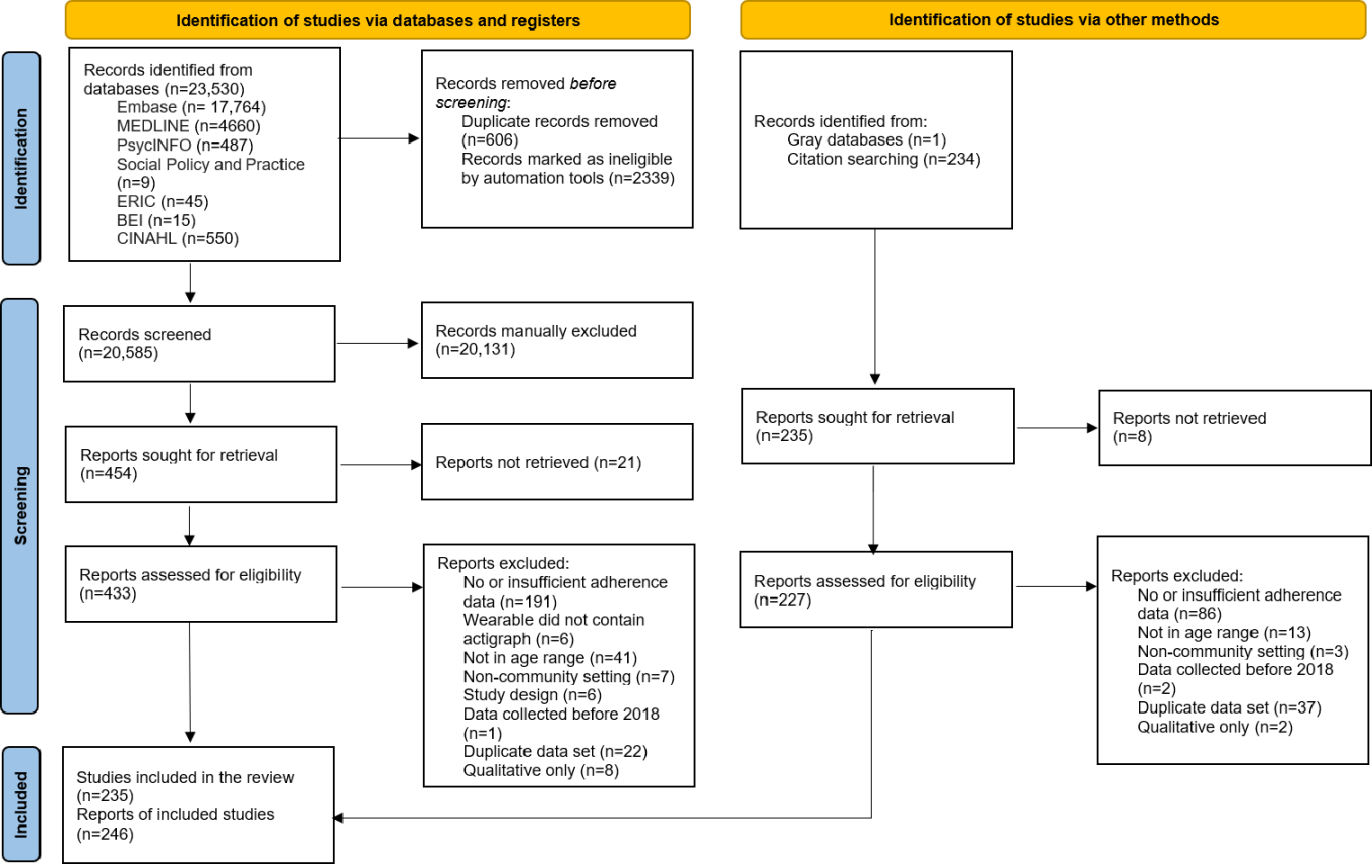
PRISMA (Preferred Reporting Items for Systematic Reviews and Meta-Analyses) flow diagram.

Searches identified 20,585 citations, 235 (n=148,161) of which were eligible (see Figure 1 and MultimediaAppendices 2  4). Additionally, Multimedia Appendix 5 provides details of individual, device, and study characteristics of all included studies, with Multimedia Appendix 6 providing a list of references for all articles included in the review.

### Summary of Individual and Study Characteristics

Europe was the most common study location (43.8%), followed by North America (27.2%). Locations also included the Western Pacific Region (11.9%), South-East Asia (9.4%), and very few from South America, Africa, and the Eastern Mediterranean Region (Table 1). Sample size ranged from 10 to 18,596 participants, with mean age ranging from 4.2 (SD 1.7) to 14.4 (SD 2.6) years. Sex distribution was largely balanced (74.9% of studies included 40%‐60% male participants), though male representation was higher in 12.8% of studies and a small number of studies included single sex samples. Ethnicity data were sparse (n=75, 31.9%) and heterogeneously reported, limiting meaningful synthesis. The most frequently reported categories were White, Black or African American, Hispanic or Latino, and Asian. A smaller number of studies reported mixed or multiracial, Native American or Alaska Native, Pacific Islander, or other participants. Full details of all ethnic categories are provided in Multimedia Appendix 5. Studies mostly included samples without health diagnosis (77%), with smaller numbers focused on participants with physical health disorders (13.2%) or neurodevelopmental or mental health conditions, in which the latter two were grouped together (6.8%).

**Table 1.** Study sample and participant characteristics across included studies (n=235).

	Studies, n (%)
Region	
Europe	103 (43.8)
North America	64 (27.2)
Western Pacific	28 (11.9)
South-East Asia	22 (9.4)
South America	4 (1.7)
Africa	4 (1.7)
Eastern Mediterranean	1 (0.4)
Multiple	3 (1.3)
Not reported	6 (2.6)
Diagnostic status	
No health diagnosis	181 (77)
Physical health diagnosis	31 (13.2)
Neurodevelopmental diagnosis	15 (6.4)
Mental health diagnosis	1 (0.4)
Mixed diagnosis	7 (2.6)
Not reported	1 (0.4)
Sex distribution	
Balanced (40%‐60% male)	176 (74.9)
Higher male (≥60%)	30 (12.8)
Lower male (≤40%)	8 (3.4)
Male only	5 (2.1)
Female only	2 (0.9)
Subgroup-specific sex distribution	9 (3.8)
Not reported	5 (2.1)

### Summary of Protocol and Actigraph Characteristics

*Actigraph* was the most featured device brand (n=146, 63.1%) used primarily for monitoring physical activity (n=123, 84.3%), with fewer focused on sleep (n=11, 9.5%) or both physical activity and sleep (n=12, 8.2%). Philips Respironics devices were used in 19 (8.1%) studies, focusing predominantly on sleep (n=10, 47.4%). Multimedia Appendix 7 provides a comprehensive overview of the actigraphs used across studies.

Protocol length ranged from 1 to 140 days. Shorter durations (≤7 d) were used in 75.3% of studies, with 7 days being the most frequent. Longer durations (≥8 d) were noted in 16.6% of studies. Commercial devices (9.8%) were evenly distributed among studies with short (n=12) and long (n=10) wear times, while research-grade devices (88.1%) favored a greater proportion of studies with short (n=166) versus long (n=31) wear times. Among undiagnosed samples, 140 of 170 followed a short protocol wear time, compared to long ones (n=30). In neurodevelopmental and mental health samples, almost all studies (n=14) followed a short wear time, except one study lasting 49 days. For physical health samples, longer wear times were more common (8 of 29 studies), see Multimedia Appendix 8.

Wear locations were most commonly the waist (47.7%), followed by the wrist (30.2%), while some studies reported multiple locations due to participant preference, multiple devices, or age-based recommendations. More details are provided in Table 2. Incentivization was reported in only 7.2% of studies (ie, financial rewards, vouchers, or sticker charts). Other strategies for improving adherence included detailed instructions (8.5%), wear log diaries (7.7%), and reminders, that is, text, phone, or in-person reminders (6.4%) [31].

**Table 2.** Overview of the protocol and actigraph characteristics (n=235).

	Studies, n (%)
Protocol length	
Shorter durations (<7 d)	44 (18.7)
7 days	133 (56.6)
Longer durations (≥8 d)	39 (16.6)
Mixed	6 (2.6)
Not reported	13 (5.5)
Wear location	
Waist	112 (47.7)
Wrist	71 (30.2)
Multiple locations	19 (8.1)
Other locations	8 (3.4)
Not reported	25 (10.6)
Type	
Research-grade	208 (88.5)
Commercial	25 (10.6)
Mixed	1 (0.4)
Not reported	1 (0.4)
Incentivization	
Incentivization	17 (7.2)
Not reported	218 (92.8)
Other support strategies	
Instructions	20 (8.5)
Wear-log diaries	18 (7.7)
Reminders	15 (6.4)
Mixed	9 (3.8)
Not reported	173 (73.6)

### Protocol Length and Average Days Worn

Deviation from protocol length was calculable for k=173 studies, of which k=22 showed low (<40%), k=122 medium (40%‐70%), and k=29 high (>70%) deviation. In short protocols (≤7 d), deviation rates were low in k=20 (11.3%) studies, medium in k=106 (59.9%) studies, and high in k=18 (10.2%). However, among long protocols (≥8 d), k=2 (5.1%) showed low deviation, k=16 (41%) medium deviation, and k=10 (25.6%) high deviation. This suggests that studies with longer monitoring periods were more than twice as likely to report high deviation rates compared to shorter protocols.

Mean wear time for adherent samples (participants meeting the required number of days to be included in the analysis) was reported in 52 studies, ranging from 2.4 to 26.1 days. Studies with shorter wear time requirements achieved higher adherence: 23 out of 40 studies with shorter protocols had participants complete ≥90% of their intended protocol wear time, while one out of 10 studies with longer protocol lengths met this threshold.

### Quality Assessment

Using the bespoke quality assessment checklist, only 8 studies (3.4%) met basic reporting standards and were deemed high quality, with the remaining 227 studies (96.6%) scored as low quality. Reporting of valid wear time, analysis wear time, and usable or missing data fared particularly poorly, with less than half of studies reporting on these domains. Multimedia Appendix 9 provides a full quality assessment report.

### Meta-Analysis

Data from 135 studies were included in the meta-analysis, with individual and pooled adherence rates as shown in Multimedia Appendix 10. Individual adherence rates showed considerable variation, ranging between 24.9% and 100%, with a pooled estimate of 81.6% (95% CI 78.7%‐84.4%) and substantial heterogeneity (*I*^2^=98.8%; τ^2^=0.17). Prediction intervals ranged from 42.8% to 100%, indicating variability across study contexts.

### Overall Adherence Rates Based on Individual, Actigraph, and Study Characteristics

**Figure 2.**
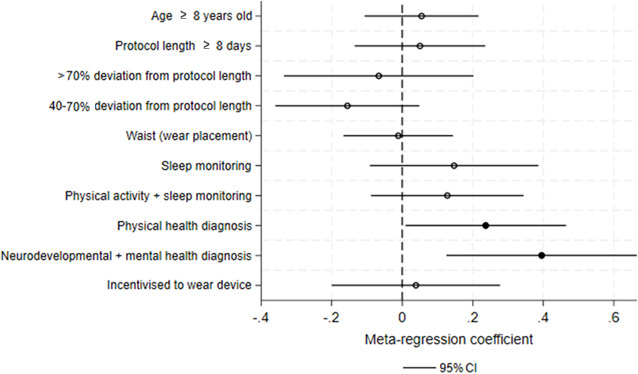
Univariable meta-regression models for associations between adherence to wearable devices and individual, device-related study design variables. Coefficients are reported relative to the following reference categories for each variable: <8 y (age), <8 d (protocol length), wrist (device placement), <40% (deviation from protocol length), physical activity monitoring (wear purpose), no diagnosis (diagnosis), and not incentivized to wear device (incentivization).

Univariable meta-regression revealed no significant association between adherence rates and age, device (purpose and placement), or study design (protocol length, deviation from the protocol length, and incentivization; Figure 2 and Table 3). However, participants with health diagnoses showed a significantly greater adherence to actigraphs (b=.236, *P*=.042, 95% CI 0.009-0.464), as did children with a neurodevelopmental or mental health diagnosis (b=.395, *P*=.004, 95% CI 0.125-0.665).

**Table 3.** Univariate meta-regressions examining the association between actigraph adherence rate and age group, diagnosis, device placement, protocol length, wear purpose, and device setup and incentivization.

Variable	β (SE)	*P* value	95% CIs
Age (≥8 y old)	0.054 (0.082)	.51	−0.106 to 0.216
Protocol length (≥8 d)	0.050 (0.094)	.59	−0.134 to 0.235
Device placement (waist)	−0.011 (0.079)	.89	−0.166 to 0.144
Deviation from protocol length			
Medium	−0.156 (0.104)	.14	−0.360 to 0.049
High	0.066 (0.137)	.63	−0.334 to 0.202
Wear purpose			
Sleep	0.147 (0.121)	.23	−0.091 to 0.385
Sleep and physical activity	0.127 (0.110)	.25	−0.088 to 0.344
Diagnosis			
Physical health	0.236 (0.116)	.04	0.009 to 0.464
Mental health	0.395 (0.137)	.004	0.125 to 0.665
Incentivization	0.038 (0.121)	.75	−0.199 to 0.277

a Coefficients are reported relative to the following reference categories for each variable: <8 y (age),<8 d (protocol length), wrist (device placement), low (deviation from protocol), physical activity (wear purpose and no diagnosis). Low, medium, and high deviations from protocol length represent (≤40%), (40%‐70%), and (>70%), respectively.

### Small-Study Effects

**Figure 3.**
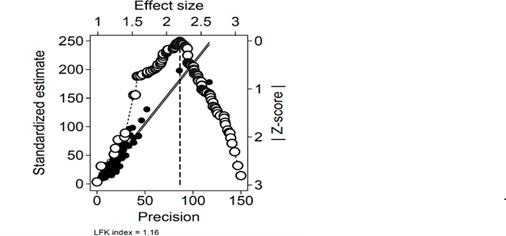
Doi plot for risk of small-study effects analysis. The Doi plot assesses small-study effects by displaying standardized effect sizes (left y-axis) against precision (bottom x-axis, inverse of SE). The upper x-axis represents actual effect sizes, while the right y-axis shows absolute *Z*-scores (standard normal deviation of effect sizes from the pooled estimate). Greater asymmetry suggests a higher likelihood of bias and small-study effects, quantified by the Luis Furuya-Kanamori (LFK) index. An LFK index <1.0 indicates no asymmetry, 1.0‐2.0 suggests minor asymmetry, and >2.0 indicates major asymmetry with a higher risk of small-study effects and publication bias. Here, LFK=1.16, concurring with the slight plot asymmetry, hence no strong evidence of bias.

The Doi plot (Figure 3) and the LFK index (1.16) indicated only minor small-study effects.

### Residual Heterogeneity and Homogeneity Tests

*I*^2^ remained greater than 98% across analyses, with significant Q-tests for homogeneity (*P*<.001). However, meta-regression explained up to 6.82% of the variance in specific models, suggesting some moderator effects.

## Discussion

### Principal Findings

This study synthesized quantitative evidence from 235 studies of actigraph adherence in children aged 5‐11 years. Across 135 studies included in the meta-analysis, pooled adherence was high at 81.6%; however, considerable variability across studies was evident, with adherence markedly lower in some studies. Children with health diagnoses, particularly neurodevelopmental or mental health conditions, demonstrated significantly higher adherence compared to their undiagnosed peers. No other factors had a statistically significant impact on adherence, including age, actigraph placement, protocol length, deviation from protocol length, study purpose, and incentivization. These findings significantly challenge views that actigraphic device adherence would be hindered by children with additional health needs and highlight these devices as a valuable opportunity for assessment and monitoring in this group [41].

While pooled adherence was encouragingly high, the results indicated substantial variability across studies. The meta-analysis reported a large *I*^2^ (98.8%), typically interpreted as high heterogeneity. However, *I*^2^ has recognized limitations in prevalence synthesis [42], as it does not reliably capture how much the true effect varies across study contexts, in part owing to the nature of proportional data and larger sample sizes inflating heterogeneity estimates [42]. Accordingly, we also reported both CIs, which indicate the precision of pooled adherence, and prediction intervals, which provide a range of expected estimates for future studies [42-44]. This review yielded prediction intervals of 42.8% to 100%, demonstrating a wide distribution of adherence across studies. While adherence was high on average, it was considerably lower in some studies. Our meta-regression findings indicate that study-level factors may partially explain this variability. Caution should therefore be exercised when generalizing pooled adherence estimates, and variability should be anticipated when developing and implementing actigraphy protocols, as these results indicate that future adherence may vary according to the sample, device, or setting under consideration. In the narrative review, studies with shorter wear durations reported higher average wear time, with nearly half reporting at least 90% compliance compared to 10% studies with longer protocols. The absence of a statistical association between protocol length and adherence may be partially explained by the distribution of device types. Commercial devices were equally common in both short and long protocol lengths, whereas research-grade wearables were mostly present in short protocols. This pattern may reflect a preference for commercial actigraphs in the development of longer wear protocols, likely based on assumptions that their enriched aesthetics and greater social acceptability improve user tolerance, ultimately leading to adequate adherence.

Accordingly, this finding suggests that most long-term actigraphic data are not of research-grade, potentially posing issues related to commercial-grade actigraphs [45]. These devices may lack data granularity and rely on nontransparent proprietary algorithms that are not subject to research scrutiny, allowing for undisclosed modifications to data processing and transfer policies. Additional risks include inconsistent data accuracy, restricted access to raw data, interoperability issues, and ethical concerns related to cloud-based storage. These factors may raise data privacy concerns and significantly limit the suitability of such devices for research purposes [46].

Children with health conditions exhibited higher adherence compared to those without any diagnoses, challenging some previous evidence on how additional health needs, particularly neurodevelopmental conditions, may hinder adherence [47]. This is likely explained through the Technological Acceptance Model, which specifies perceived usefulness as the strongest predictor of eHealth innovation engagement [14]. Children and their families may view wearable devices as tools to better understand or manage their conditions, potentially leading to additional support or encouragement from caregivers [48,49]. Moreover, these populations are likely more familiar and comfortable with health-related monitoring, resulting in greater device tolerance [50]. Additionally, much of the “no diagnosis” sample came from larger cohort studies, where adherence data by health status was not reported, thus limiting comparison with more homogeneous samples with a health condition. These results provide practical insights by illustrating the feasibility of actigraphy use in these populations for research purposes and by highlighting the potential to integrate wearable measurement into clinical systems to support diagnostic and treatment pathways.

No significant differences in adherence were observed depending on device purpose, wear location, or incentivization of participants. While there is scarce data to contextualize the impact of device purpose on adherence, Tudor-Locke et al reported higher average wear time for 24-hour monitoring periods compared to waking hours only [10]. Future research could benefit from distinguishing between within-day wear duration and the purpose of wearing the device to provide a more relevant measure of acceptability. Our findings align with the limited evidence, indicating that for primary school-aged children, actigraph placement can be flexibly determined based on study goals [11,12,28]. Evidence on the influence of incentivization is mixed, and there is disagreement on what type of incentives are more effective in increasing adherence to actigraphy [15,30]. This is likely fueled by the lack of standardization in the definition of an “incentive” in this context, and some evidence also suggests that strategies such as wear-log diaries or reminders can also increase adherence [30,31]. This variation in what is considered an “incentive” was reflected in the studies included in our review, making it difficult to quantify its overall impact on adherence.

### Strengths and Limitations

This research has several key strengths. It is the most comprehensive study of its kind, providing a quantitative assessment of actigraphy adherence in young children. Building on the existing acceptability literature, it presents the first attempt to pool adherence rates within a representative sample of 135 studies with 64,541 participants, from diverse populations and study designs as well as examining 13 device brands, including both research and commercial grade options [27,40,51-53]. Additionally, we employed statistical methods recommended for pooling prevalence data, including prediction intervals and Doi plots, which are often omitted in reports of proportional meta-analyses [42,44]. Specifically, Doi plots and the corresponding LFK index were used to assess small-study effects, rather than traditional approaches such as funnel plots and Egger tests. The latter method lacks reliability and interpretability for proportional data because its frequently skewed distribution violates the assumption of funnel plot symmetry [54]. Alternatively, Doi plots afford better sensitivity and specificity by accommodating skewed distributions and greater heterogeneity [40].

Notwithstanding the strengths of this research, the results should be interpreted in the context of several limitations. First, the observed pooled adherence may be overestimated owing to the manual methods used to calculate the number of participants who were adherent. Importantly, nonwear issues relating to device error, for example, failure to initialize or malfunctioning, were not categorized as nonadherence. Accordingly, cases where such issues led to discontinuing device use were not represented in overall pooled adherence.

Second, it was not possible to assess adherence rates by important demographic factors such as ethnicity, which was scarcely reported (31.9%) and varied substantially in how it was reported, prohibiting categorization and meaningful synthesis. This inconsistency limits the ability to examine equality in wearable technological use and may overlook critical differences in adherence across ethnic groups. Prior research in adults with cardiovascular disease has documented significant differences in wearable technology use across ethnicities [55]. Consistent reporting of key sociodemographic information is therefore essential to understand whether similar inequalities exist in actigraphy adherence among children and inform inclusive research practices. Third, for several variables, nuances in the data were simplified to enable statistical comparison between studies. For example, we did not differentiate between the type of days worn, that is, day or night, week or weekend, or school holidays. Similarly, where studies reported multiple measurement periods, only baseline measurements were included. While cohesive categories were necessary, future research would benefit from evaluating the impact of methodological complexities on adherence to actigraphs. Several of the devices reviewed in this study have now been discontinued, which may limit the relevance of these results. Risk of bias was assessed using a bespoke quality assessment checklist developed to capture domains integral to adherence reporting. However, as this tool is not yet formally validated, results cannot be directly compared to reviews of prevalence data that use established tools for assessing bias. Moreover, for studies scoring low quality, it is possible that methodological details are reported elsewhere and were therefore undetected rather than omitted by researchers, thus underrepresenting research quality. Finally, although extensive, our electronic database searches were last updated on the 24th of January 2023. Given the scope of the review and the large volume of records retrieved for screening, rerunning updated searches to include studies published after this data was beyond the resources available to our research team. Future work should build on this review to assess how our findings align with the most recent literature.

### Implications

Our findings revealed inconsistent reporting of information critical to understanding actigraphy adherence across most studies. Notably, nearly a quarter of studies (24.6%) failed to report the number of valid days to be included in the analysis. This figure has increased from the 17% previously reported for actigraphy studies involving children, demonstrating that reporting deficits remain a prominent issue [3]. Similarly, we detected frequent unexplained protocol deviation in relation to wear-time criteria, highlighting the need for more transparent and consistent reporting of decision rules around data inclusion and processing to prevent misinterpretation as bias [3]. The degree of studies failing to achieve basic reporting standards limits our understanding of acceptability and the factors that impact adherence.

To address these reporting deficiencies, we encourage better reporting of actigraphy data collection and processing, including the development of standard reporting guidelines for actigraphy adherence in pediatric populations, as also recommended by other researchers [31,56]. Such guidelines should expand on existing recommendations, for example, Berger et al [36], and include key parameters such as decision-making rules for analyzing wear time and clear reasons for missing data. They should also distinguish between human factors directly related to adherence or technical factors such as device breakage or failure to initialize. Establishing consensus-driven reporting norms will enhance transparency, improve replicability, and enable better comparison across studies, thus strengthening the adherence knowledge base that will be vital for planning future research or clinical applications of these devices in conjunction with young people.

Moreover, our findings of variable adherence highlight the importance of adapting study designs and support strategies to target populations and settings. This will optimize acceptability and data completeness. Improved reporting practices around incentivization and within-study support will facilitate the evaluation and application of these approaches in future research.

### Conclusion

This review demonstrates generally high adherence to actigraphy among primary school–aged children while identifying the need for diverse methodologies and device options to better understand factors influencing adherence. It provides key insights into actigraphic tolerance and encouragingly found high adherence in groups previously considered less likely to sustain use, such as those with neurodevelopmental, mental, or physical health conditions. However, reliance on commercial devices for long-term data raises quality concerns, while research-grade device prevalence in short-term studies suggests the need for improved design. As actigraphy gains traction, particularly in pharmaceutical trials, the declining quality of methodological reporting emphasizes the need for standardized assessment tools to assist future adherence data synthesis.

## Supplementary material

10.2196/79718 Multimedia Appendix 1 Changes to preregistered protocol.

10.2196/79718 Multimedia Appendix 2 Search methods and terms.

10.2196/79718 Multimedia Appendix 3 Quality assessment checklist.

10.2196/79718 Multimedia Appendix 4 List of articles excluded at full text and justification.

10.2196/79718 Multimedia Appendix 5 Summary of extracted data from included studies.

10.2196/79718 Multimedia Appendix 6 References included in the systematic review and meta-analysis

10.2196/79718 Multimedia Appendix 7 Overview of actigraphic device usage and characteristics in the included studies.

10.2196/79718 Multimedia Appendix 8 Protocol wear time distribution across health diagnosis categories.

10.2196/79718 Multimedia Appendix 9 Quality assessment scores of included studies.

10.2196/79718 Multimedia Appendix 10 Individual and pooled adherence prevalence estimates.

10.2196/79718 Multimedia Appendix 11 Full list of members of the CAMHS Digital Lab team**.**

10.2196/79718 Checklist 1 PRISMA checklist.

10.2196/79718 Checklist 2 MOOSE checklist.

## Abbreviations

LFK: Luis Furuyama-Kanamori
MOOSE: Meta-analyses of Observational Studies in Epidemiology
PRISMA: Preferred Reporting Items for Systematic Reviews and Meta-Analyses
