# Supplementary material for: Adherence to Actigraphic Devices in Elementary School–Aged Children: Systematic Review and Meta-Analysis
Source: J Med Internet Res. 2025 Nov 3;27:e79718. doi: 10.2196/79718 (PMC12582557; doi:10.2196/79718)
Supplement: Multimedia Appendix 1 [file jmir-v27-e79718-s001.docx]

**Multimedia appendix 1. Changes to pre-registered protocol**

In our PROSPERO-registered protocol (CRD42021232466), we stated

- "Where provided, details regarding the actigraphic device will also be recorded, including device name, purpose (eg, sleep, obesity, ADHD symptom tracking), features (eg, material, design and functionality), body-worn location and mode of delivery (eg, on its own or part of a multi-faceted tool)." However, to reduce heterogeneity and facilitate analysis, device purpose was simplified to physical activity monitoring, sleep monitoring, or both. Additionally, features and whether the device was part of a multi-faceted tool were rarely reported in preliminary searches, so this information was not extracted.
- “All text relating to relevant qualitative findings will be collected from the Results section of included papers, to record themes and subthemes pertaining to child-worn actigraph acceptability and engagement. Particular attention will be paid to any design or contextual factors that hinder or promote actigraphic device use in the target population. Where available, this will include direct participant quotes to afford greater data granularity at the data synthesis stage. Additionally, descriptions of the research methods used to assess acceptability (eg, structured questionnaire with space to report free text, structured or semi-structured interview or focus groups) and the information reporting source (eg, child, caregiver, or teacher) will be collected.” Unfortunately, due to the large number of studies providing some indication of behavioural acceptability, the study focus was refined from both quantitative (adherence) and qualitative (affective/cognitive) markers of acceptability to solely, allowing for a more detailed evaluation of behavioral adherence.
