# Supplementary material for: Adherence to Actigraphic Devices in Elementary School–Aged Children: Systematic Review and Meta-Analysis
Source: J Med Internet Res. 2025 Nov 3;27:e79718. doi: 10.2196/79718 (PMC12582557; doi:10.2196/79718)
Supplement: Multimedia Appendix 2 [file jmir-v27-e79718-s002.docx]

**Multimedia appendix 2. Search methods and terms**

With the assistance of librarian, we systematically searched Embase, MEDLINE, PsycINFO, and Social Policy and Practice via OVID, and Education Resources Information Centre (ERIC), British Education Index, and Cumulative Index to Nursing and Allied Health Literature (CINAHL) via EBSCO, using tailored strategies developed with librarian assistance from January 2018 to twenty-fourth January 2023. Forward and backward citation searches were conducted in Web of Science Core Collection, and Google Scholar where necessary. Grey literature searches were conducted on PsycEXTRA and HMIC. To be included studies had to be written in English and could be published or unpublished.

**Search strategy and results from each electronic database**

*Last search: January 24^th^, 2023*

**EMBASE (OVID)**

**Search terms:** 1. child*.ab,ti. 2. exp child/ 3. primary school.ab,ti. 4. youth*.ab,ti. 5. kindergar#en.ab,ti. 6. kid*.ab,ti. 7. pupil*.ab,ti. 8. juvenile*.ab,ti. 9. exp juvenile/ 10. young people*.ab,ti. 11. 1 or 2 or 3 or 4 or 5 or 6 or 7 or 8 or 9 or 10 12. (actigraph* or actimet* or actograp* or actomet* or acceleromet*).ab,ti. 13. motor activity.ab,ti. 14. exp motor activity/ 15. Fitbit.ab,ti. 16. ((electronic or remote or wearable or fitness or activity) adj3 (track* or monitor* or wearable* or device* or technolo*)).ab,ti. 17. step count*.ab,ti. 18. 12 or 13 or 14 or 15 or 16 or 17 19. acceptability.ab,ti. 20. experience*.ab,ti. 21. perception*.ab,ti. 22. feasibility.ab,ti. 23. feedback.ab,ti. 24. design*.ab,ti. 25. usability.ab,ti. 26. practicability.ab,ti. 27. willingness.ab,ti. 28. usefulness.ab,ti. 29. engagement.ab,ti. 30. opinion*.ab,ti. 31. 19 or 20 or 21 or 22 or 23 or 24 or 25 or 26 or 27 or 28 or 29 or 30 32. 11 and 18 and 31

**Results:** 17764 hits

**MEDLINE (OVID)**

**Search terms:** 1. child*.ab,ti. 2. exp child/ 3. primary school.ab,ti. 4. youth*.ab,ti. 5. kindergar#en.ab,ti. 6. kid*.ab,ti. 7. pupil*.ab,ti. 8. juvenile*.ab,ti. 9. young people*.ab,ti. 10. 1 or 2 or 3 or 4 or 5 or 6 or 7 or 8 or 9 11. (actigraph* or actimet* or actograp* or actomet* or acceleromet*).ab,ti. 12. motor activity.ab,ti. 13. exp motor activity/ 14. Fitbit.ab,ti. 15. ((electronic or remote or wearable or fitness or activity) adj3 (track* or monitor* or wearable* or device* or technolo*)).ab,ti. 16. step count*.ab,ti. 17. 11 or 12 or 13 or 14 or 15 or 16 18. acceptability.ab,ti. 19. experience*.ab,ti. 20. perception*.ab,ti. 21. feasibility.ab,ti. 22. feedback.ab,ti. 23. design*.ab,ti. 24. usability.ab,ti. 25. practicability.ab,ti. 26. willingness.ab,ti. 27. usefulness.ab,ti. 28. engagement.ab,ti. 29. opinion*.ab,ti. 30. 18 or 19 or 20 or 21 or 22 or 23 or 24 or 25 or 26 or 27 or 28 or 29 31. 10 and 17 and 30

**Results:** 4660

**PsychINFO (OVID)**

**Search terms:** 1. child*.ab,ti. 2. primary school.ab,ti. 3. youth*.ab,ti. 4. kindergar#en.ab,ti. 5. kid*.ab,ti. 6. pupil*.ab,ti. 7. juvenile*.ab,ti. 8. young people*.ab,ti. 9. 1 or 2 or 3 or 4 or 5 or 6 or 7 or 8 10. (actigraph* or actimet* or actograp* or actomet* or acceleromet*).ab,ti. 11. motor activity.ab,ti. 12. Fitbit.ab,ti. 13. ((electronic or remote or wearable or fitness or activity) adj3 (track* or monitor* or wearable* or device* or technolo*)).ab,ti. 14. step count*.ab,ti. 15. 10 or 11 or 12 or 13 or 14 16. acceptability.ab,ti. 17. experience*.ab,ti. 18. perception*.ab,ti. 19. feasibility.ab,ti. 20. feedback.ab,ti. 21. design*.ab,ti. 22. usability.ab,ti. 23. willingness.ab,ti. 24. usefulness.ab,ti. 25. engagement.ab,ti. 26. opinion*.ab,ti. 27. 16 or 17 or 18 or 19 or 20 or 21 or 22 or 23 or 24 or 25 or 26 28. 9 and 15 and 27

**Results:** 487

**Social Policy and practice via OVID**

**Search terms:** 1. child*.ab,ti. 2. primary school.ab,ti. 3. youth*.ab,ti. 4. kid*.ab,ti. 5. pupil*.ab,ti. 6. juvenile*.ab,ti. 7. young people*.ab,ti. 8. 1 or 2 or 3 or 4 or 5 or 6 or 7 9. (actigraph* or actimet* or actograp* or actomet* or acceleromet*).ab,ti. 10. ((electronic or remote or wearable or fitness or activity) adj3 (track* or monitor* or wearable* or device* or technolo*)).ab,ti. 11. 9 or 10 12. acceptability.ab,ti. 13. experience*.ab,ti. 14. perception*.ab,ti. 15. feasibility.ab,ti. 16. feedback.ab,ti. 17. design*.ab,ti. 18. usability.ab,ti. 19. willingness.ab,ti. 20. usefulness.ab,ti. 21. engagement.ab,ti. 22. opinion*.ab,ti. 23. 12 or 13 or 14 or 15 or 16 or 17 or 18 or 19 or 20 or 21 or 22 24. 8 and 11 and 23

**Results:** 9

**ERIC (EBSCOhost)**

**Search terms:** S1 - TI child* OR AB child* OR TI "primary school" OR AB "primary school" OR TI youth* OR AB youth* OR TI kindergar?en OR AB kindergar?en OR TI kid* OR AB kid* OR TI pupil* OR AB pupil* OR TI juvenile* OR AB juvenile* OR TI "young people*" OR AB "young people*" S2 - TI ( (actigraph* or actimet* or actograp* or actomet* or acceleromet*) ) OR AB ( (actigraph* or actimet* or actograp* or actomet* or acceleromet*) ) OR TI "motor activity" OR AB "motor activity" OR TI fitbit OR AB fitbit OR TI ( ((electronic or remote or wearable or fitness or activity) adj3 (track* or monitor* or wearable* or device* or technolo*)) ) OR AB ( ((electronic or remote or wearable or fitness or activity) adj3 (track* or monitor* or wearable* or device* or technolo*)) ) OR TI "step count*" OR AB "step count*" S3 - TI acceptability OR AB acceptability OR TI experience* OR AB experience* OR TI perception* OR AB perception* OR TI feasibility OR AB feasibility OR TI feedback OR AB feedback OR TI design* OR AB design* OR TI usability OR AB usability OR TI practicability OR AB practicability OR TI willingness OR AB willingness OR TI usefulness OR AB usefulness OR TI engagement OR AB engagement OR TI opinion* OR AB opinion* S4 – S1 AND S2 AND S3

**Results:** 45

**British Education Index (EBSCOhost)**

**Search terms:** S1 - TI child* OR AB child* OR TI "primary school" OR AB "primary school" OR TI youth* OR AB youth* OR TI kindergar?en OR AB kindergar?en OR TI kid* OR AB kid* OR TI pupil* OR AB pupil* OR TI juvenile* OR AB juvenile* OR TI "young people*" OR AB "young people*" S2 - TI ( (actigraph* or actimet* or actograp* or actomet* or acceleromet*) ) OR AB ( (actigraph* or actimet* or actograp* or actomet* or acceleromet*) ) OR TI "motor activity" OR AB "motor activity" OR TI fitbit OR AB fitbit OR TI ( ((electronic or remote or wearable or fitness or activity) adj3 (track* or monitor* or wearable* or device* or technolo*)) ) OR AB ( ((electronic or remote or wearable or fitness or activity) adj3 (track* or monitor* or wearable* or device* or technolo*)) ) OR TI "step count*" OR AB "step count*" S3 - TI acceptability OR AB acceptability OR TI experience* OR AB experience* OR TI perception* OR AB perception* OR TI feasibility OR AB feasibility OR TI feedback OR AB feedback OR TI design* OR AB design* OR TI usability OR AB usability OR TI practicability OR AB practicability OR TI willingness OR AB willingness OR TI usefulness OR AB usefulness OR TI engagement OR AB engagement OR TI opinion* OR AB opinion* S4 – S1 AND S2 AND S3

**Results:** 15

**CINAHL (EBSCOhost)**

**Search terms:** S1 - (MH "Child+") OR TI child* OR AB child* OR TI "primary school" OR AB "primary school" OR TI youth* OR AB youth* OR TI kindergar?en OR AB kindergar?en OR TI kid* OR AB kid* OR TI pupil* OR AB pupil* OR TI juvenile* OR AB juvenile* OR TI "young people*" OR AB "young people*" S2 - (MH "Motor Activity+") OR TI ( (actigraph* or actimet* or actograp* or actomet* or acceleromet*) ) OR AB ( (actigraph* or actimet* or actograp* or actomet* or acceleromet*) ) OR TI "motor activity" OR AB "motor activity" OR TI Fitbit OR AB Fitbit OR TI ( ((electronic or remote or wearable or fitness or activity) adj3 (track* or monitor* or wearable* or device* or technolo*)) ) OR AB ( ((electronic or remote or wearable or fitness or activity) adj3 (track* or monitor* or wearable* or device* or technolo*)) ) OR TI "step count*" OR AB "step count*" S3 - TI acceptability OR AB acceptability OR TI experience* OR AB experience* OR TI perception* OR AB perception* OR TI feasibility OR AB feasibility OR TI feedback OR AB feedback OR TI design* OR AB design* OR TI usability OR AB usability OR TI practicability OR AB practicability OR TI willingness OR AB willingness OR TI usefulness OR AB usefulness OR TI engagement OR AB engagement OR TI opinion* OR AB opinion* S4 – S1 AND S2 AND S3

**Results:** 550

**After merging and de-duplication: 20585 references**
