# Supplementary material for: Adherence to Actigraphic Devices in Elementary School–Aged Children: Systematic Review and Meta-Analysis
Source: J Med Internet Res. 2025 Nov 3;27:e79718. doi: 10.2196/79718 (PMC12582557; doi:10.2196/79718)
Supplement: Multimedia Appendix 3 [file jmir-v27-e79718-s003.docx]

| **Multimedia appendix 3: Quality assessment checklist** | |
| --- | --- |
| **Criterion**^a^ | **Question**^b^ |
| **1** | Actigraphic device type: Any mention of the actigraphic device brand/manufacturer or model? |
| **2** | Actigraphic device placement/location: Any mention of where the actigraphic device was worn on the participants’ body i.e. wrist, waist. |
| **3** | Information about device allocation/collection: Any mention of how devices were allocated to and/or whether they were collected from participants. |
| **4** | Instructions about the actigraphic device use: Any mention of instructions (however brief) given to participants about how to use the actigraphic device. |
| **5** | Diary/contextualising information: Any mention of if/how the study included a diary or other methods of collecting contextualising information regarding participants’ usage of the actigraphic device i.e. pressing an on-device maker to denote sleep initiation. |
| **6** | Valid wear time: Reporting the actigraphic device's valid wear time in the study protocol. |
| **7** | Analysis wear time: Reporting what was considered as a minimum amount of valid wear time for statistical analysis. |
| **8** | Usable and missing data: Reporting rates of usable and missing data whether full or partially explained. |
| ^a For each criterion, studies were assigned a score of 0 if the criterion was not met or 1 if it was met. If a study did not provide sufficient data to evaluate a criterion, it was given a score of 0, indicating that the criterion was not fulfilled. b Study quality was evaluated using a customised quality assessment checklist adapted from Berger et al’s recommendations for the use of actigraphic devices in research. [22]^ | |
