# Supplementary material for: Adherence to Actigraphic Devices in Elementary School–Aged Children: Systematic Review and Meta-Analysis
Source: J Med Internet Res. 2025 Nov 3;27:e79718. doi: 10.2196/79718 (PMC12582557; doi:10.2196/79718)
Supplement: Multimedia Appendix 4 [file jmir-v27-e79718-s004.docx]

**Multimedia appendix 4. List of articles excluded at full text and justification**

**Full text not available**

Blackwell JE, Kingshott RN, Weighall AR, Elphick HE, Nash HM. The Paediatric Narcolepsy Project: The relationships between sleep, physical activity, cognitive function and psychosocial well-being in children with narcolepsy. 2019.

Bastien L, Theoret R, Bernier A, Godbout R. et al. Habitual sleep and intraindividual variability of gifted children: An actigraphy study. 2022.

Dlugonski D, Schwab L, DuBose KD. Feasibility of the Mothers and Daughters Moving Together Physical Activity Intervention. 2022.

Douglas H, Raywood E, Kapoor K, et al. Assessment of activity tracker wear-time in longitudinal measurement of physical activity in children and young people with CF. 2019.

Gellner RA, Campolettano ET, Smith EP, Rowson S. et al. Are specific players more likely to be involved in high-magnitude head impacts in youth football? 2019.

Hamilton KC, Richardson MT, McGraw S, Owens T, Higginbotham JC. A controlled evaluation of a CBPR intervention's effects on physical activity and the related psychosocial constructs among minority children in an underserved community. 2020.

Leikauf JE, Bueno AN, Correa C, Peris Sempere V, Williams LM. 6.58 APPLE WATCH PILOT FOR YOUTH WITH ADHD. 2019.

McGovern J, Drewson SR, Hope A, Konopack JF. Gender differences in a youth physical activity intervention: Movement levels and children's perceptions. 2020.

Oreskovic NM, Neumeyer AM, Duggan MP, Kuhlthau KA. Assessment of walking routes as a possible approach for promoting physical activity in children with autism spectrum disorder: Brief report. 2020.

Zimmo L, Almudahka F, Ibrahim I, Al-kuwari MG, Farooq A. et al. Moderate to vigorous physical activity during physical education, recess, and class time among elementary school children in Qatar. 2019.

Visser E, Mazzoli E, Hinkley T, Utesch T, Barnett L. et al. Are children with a higher sense of perceived motor competency more physically active one year later? 2020.

Zezhao C, Huaijin X, Konishi D, Weimo Zhu WZ. Relationship among preschool children's fundamental motor skills, physical activity, and physical fitness: A national study. Presented at: 2021 ACSM Annual Meeting & World Congresses [Virtual]; June 1-5, 2021.

Smits A, Lammers GJ, Fazel M, Janssens K. et al. Sleep problems in people with intellectual disability (ID); diagnosis and treatment. 2019

Spittle AJ, Fitz Gerald TL, Mentiplay BF, et al. Physical activity in very preterm and term-born preschool-age children.

Steur L, Grootenhuis M, Van Eijkelenburg N, et al. Subjective but not objective increase in sleep problems during dexamethasone treatment in pediatric acute lymphoblastic leukemia. 2018.

Suresh S, Lindhiem O, Goel M. et al. Application of sensors and machine learning in the evaluation of hyperactivity in children. 2020.

Tian P, Choppara S, Harris A, Letourneau-Freiberg LR, Greeley AW. Participants with KATP-related neonatal diabetes (KATPNDM) experience more sleep disruption than sibling controls. 2020.

Voutsas G, Moraes T, Subbarao P, Brook J, To T, Narang I. et al. The impact of sleep disturbance on physical health in a severe asthmatic pediatric population. 2022.

Yu JJ, Tsai CL, Pan CY, Li R, Sit CHP. The relationship between physical activity and inhibition in children with and without motor impairments. 2020.

Limmeroth J, Raboldt M. et al. I do what I like: 8- to 10-year-old children's physical activity behavior is already interrelated with their automatic affective processes. 2022.

Gu X, Chen S, Zhang X. et al. Young Hispanic and non-Hispanic children's fundamental motor competence and physical activity behaviors. 2019.

Barreira TV, Broyles ST, Pietrobelli A, et al. Epidemiological transition in physical activity and sedentary time in children. 2019.

Yi L, Mason TB, Dunton GF, et al. Longitudinal associations between neighborhood park and open space access and children's accelerometer-assessed physical activity: Evidence from the MATCH study. 2021.

Robinson KA. Associations of actigraphy measures of sleep duration and continuity with executive function, vigilance, and fine motor control in children with snoring and mild sleep-disordered breathing. 2023.

Fletcher FE, et al. The association between anxiety symptoms and sleep in school-aged children: A combined insight from the Children's Sleep Habits Questionnaire and actigraphy. 2018.

Brazendale K, Rayan S, Leon A, et al. Obesogenic behaviors of rural children on school and nonschool days. 2021.

Kvalo SE, Natlandsmyr IK. The effect of physical-activity intervention on children’s health-related quality of life. 2021.

Lätt E, Mäestu J, Jürimäe J. Associations of accumulated time in bouts of sedentary behavior and moderate-to-vigorous physical activity with cardiometabolic health in 10- to 13-year-old boys. 2018.

Sharma G, Stewart T, Duncan S. et al. Effects of a curriculum-integrated dance program on children's physical activity. 2020.

**No adherence data**

Robbins J, et al. A school- and home-based intervention to improve adolescents' physical activity and healthy eating: A pilot study. 2020.

Chen S, Zhu X, Androzzi J, Nam YH. Evaluation of a concept-based physical education unit for energy balance education. 2018.

Reid RER, Fillon A, Thivel D, et al. Can anthropometry and physical fitness testing explain physical activity levels in children and adolescents with obesity? 2020.

Rubin DA, Wilson KS, Dumont-Driscoll M, Rose DJ. Effectiveness of a parent-led physical activity intervention in youth with obesity. 2019.

Korcz A, Krzysztoszek J, Lopatka M, Ludwiczak M, Gorska P, Bronikowski M. et al. The role of family time together in meeting the recommendation for physical activity among primary school children. 2020.

Liang X, Qiu H, Sit CHP. The mediating role of resilience in the association between MVPA and psychological ill-being in children with ADHD. 2022.

Sanabra M, Gomez-Hinojosa T, Alcover C, Sans O, Alda JA. Effects of stimulant treatment on sleep in attention deficit hyperactivity disorder (ADHD). 2021.

Amin SA, Duquesnay PJ, Wright CM, Chui K, Economos CD, Sacheck JM. The association between perceived athletic competence and physical activity: Implications for low-income schoolchildren. 2018.

Visser EL, Mazzoli E, Hinkley T, Lander NJ, Utesch T, Barnett LM. Are children with higher self-reported wellbeing and perceived motor competence more physically active? A longitudinal study. 2019.

Willis J, Hophing L, Ronen GM. Youth with epilepsy: Their insight into participating in enhanced physical activity study. 2018.

Armstrong B, Trude ACB, Johnson C, et al. CHAMP: A cluster randomized-control trial to prevent obesity in child care centers. 2019.

Aljahdali AA, Baylin A, Ruiz-Narvaez EA, et al. Sedentary patterns and cardiometabolic risk factors in Mexican children and adolescents: Analysis of longitudinal data. 2022.

Ballester P, Martinez Madrid MJ, Canet T, Richdale AL, Peiro AM. Sleep problems across a lifespan of children, adolescents, and adults with autism spectrum disorder and intellectual disability at a glance. 2020.

Baquet G, Aucouturier J, Gamelin FX, Berthoin S. et al. Longitudinal follow-up of physical activity during school recess: Impact of playground markings. 2018.

Bartelink N, van Assema P, Kremers S, Savelberg H, Gevers D, Jansen M. et al. Unravelling the effects of the Healthy Primary School of the Future: For whom and where is it effective? 2019.

Bartholomew JB, Golaszewski NM, Jowers E, et al. Active learning improves on-task behaviors in 4th grade children. 2018.

Centeio EE, Barcelona J, Fahlman M, Kulik N, Shen B, Garn A, et al. Differences of in-school moderate to vigorous physical activity among fourth grade urban youth. 2021.

Chandler JL, Brazendale K, Drenowatz C, et al. Structure of physical activity opportunities contribution to children's physical activity levels in after-school programs. 2019.

Chiva-Bartoll O, Marave-Vivas M, Salvador-Garcia C, Valverde-Esteve T. et al. Impact of a physical education service-learning programme on ASD children: A mixed-methods approach. 2021.

Clevenger KA, McNarry MA, Mackintosh KA, Berrigan D. et al. Association of recess provision with elementary school-aged children's physical activity, adiposity, and cardiorespiratory and muscular fitness. 2022.

Crozier M, Wasenius NS, Denize KM, da Silva DF, Nagpal TS, Adamo KB. Evaluation of afterschool activity programs' (ASAP) effect on children's physical activity, physical health, and fundamental movement skills. 2022.

da Costa BGG, da Silva KS, Bandeira AS, Martins CR, Vieira JAJ, Petroski EL. Pattern of sedentary behavior in different periods of school time of Brazilian adolescents. 2019.

Ee J, Parry S, de Oliveira BI, McVeigh JA, Howie E, Straker L. et al. Does a classroom standing desk intervention modify standing and sitting behaviour and musculoskeletal symptoms during school time and physical activity during waking time? 2018.

Elish PN, Bryan CS, Boedeker PJ, et al. The longitudinal association between objectively measured school-day physical activity and academic achievement in US elementary school students. 2022.

Fairclough SJ, Weaver RG, Johnson S, Rawlinson J. et al. Validation of an observation tool to assess physical activity-promoting physical education lessons in high schools: SOFIT. 2018.

Famelia R, Tsuda E, Bakhtiar S, Goodway JD. Relationships among perceived and actual motor skill competence and physical activity in Indonesian preschoolers. 2018.

Farbo D, Maler LC, Rhea DJ. The preliminary effects of a multi-recess school intervention: Using accelerometers to measure physical activity patterns in elementary children. 2020.

Faulkner G, Bassett-Gunter R, White L, Berry TR, Tremblay MS. Can The Moblees TM move Canadian children? Investigating the impact of a television program on children's physical activity. 2018.

Feng H. et al. Mental disorders and sleep and circadian rhythm dysregulation in the offspring of parents with bipolar disorder. 2022.

Fochesatto CF, Brand C, Dias AF, et al. Role of nutritional status and physical activity in the relationship between sleep quality and cardiometabolic profile of children. 2021.

Garden EM, Pallan M, Clarke J, et al. Relationship between primary school healthy eating and physical activity-promoting environments and children's dietary intake, physical activity, and weight status: A longitudinal study in the West Midlands, UK. 2020.

Goihl T, Ihlen EAF, Bardal EM, Roeleveld K, Ustad A, Braendvik SM. Effects of ankle-foot orthoses on acceleration and energy cost of walking in children and adolescents with cerebral palsy. 2021.

Gomez-Bruton A, Arenaza L, Medrano M, et al. Associations of dietary energy density with body composition and cardiometabolic risk in children with overweight and obesity: Role of energy density calculations, under-reporting energy intake, and physical activity. 2019.

Gomez-Del-Rio N, Gonzalez-Gonzalez CS, Toledo-Delgado PA, Munoz-Cruz V, Garcia-Penalvo F. et al. Health promotion for childhood obesity: An approach based on self-tracking of data. 2020.

Goon S, Kontulainen S, Muhajarine N. et al. Neighborhood built environment measures and association with physical activity and sedentary time in 9–14-year-old children in Saskatoon, Canada. 2020.

Hurst HT, Rylands L, Atkins S, Enright K, Roberts SJ. Profiling of translational and rotational head accelerations in youth BMX with and without neck brace. 2018.

Ishihara T, Sugasawa S, Matsuda Y, Mizuno M. et al. Relationship between sports experience and executive function in 6–12-year-old children: Independence from physical fitness and moderation by gender. 2018.

Hall A, Wolfenden L, Shoesmith A, et al. The impact of an implementation intervention that increased school's delivery of a mandatory physical activity policy on student outcomes: A cluster-randomised controlled trial. 2022.

Katzmarzyk PT, Broyles ST, Chaput JP, et al. Sources of variability in childhood obesity indicators and related behaviors. 2018.

Khan M, Bell R. et al. Effects of a school-based intervention on children's physical activity and healthy eating: A mixed-methods study. 2019.

King-Dowling S, Rodriguez C, Missiuna C, Timmons BW, Cairney J. et al. Health-related fitness in preschool children with and without motor delays. 2018.

Kobak M, Lepp A, Rebold M, Glickman E, Barkley JE. The experimental effect of parent versus peer influence on children's physical activity and sedentary behavior. 2021.

Kohler M, Sandiford C, Schilds L, Payne JD. Memory for emotional images across sleep versus wake in school-aged children. 2022.

Leikauf JE, Correa C, Bueno AN, Sempere VP, Williams LM. StopWatch: Pilot study for an Apple Watch application for youth with ADHD. 2021.

Loiacono B, Sunnquist M, Nicholson L, Jason LA. Activity measurement in pediatric chronic fatigue syndrome. 2022.

Lourenco J, Rodrigues C, Flores F, Soares D. et al. Physical activity time and intensity in physical education during the COVID-19 pandemic. 2022.

Masini A, Sanmarchi F, Kawalec A, et al. Mediterranean diet, physical activity, and family characteristics associated with cognitive performance in Italian primary school children: Analysis of the I-MOVE project. 2022.

Massey WV, Stellino MB, Geldhof J. et al. An observational study of recess quality and physical activity in urban primary schools. 2020.

Kwon S, Kim Y, Bai Y, Burns RD, Brusseau TA, Byun W. et al. Validation of the Apple Watch for estimating moderate-to-vigorous physical activity and activity energy expenditure in school-aged children. 2021.

Salvador-García C, Santágueda-Villanueva M, Valverde-Esteve T, Chiva-Bartoll Ó. Socio-ecological correlates of physical activity in children with autism spectrum disorder: A cross-sectional study in Spain. 2022.

Fishbein AB, Lor J, Penedo FJ, Forrest CB, Griffith JW, Paller AS. Patient-reported outcomes for measuring sleep disturbance in pediatric atopic dermatitis: Cross-sectional study of the Patient Reported Outcomes Measurement Information System pediatric sleep measures and actigraphy. 2023.

Schmidt M, Mavilidi MF, Singh A, Englert C. et al. Combining physical and cognitive training to improve kindergarten children's executive functions: A cluster randomized controlled trial. 2020.

Vetter M, O'Connor HT, O'Dwyer N, Chau J, Orr R. et al. "Maths on the move": Effectiveness of physically active lessons for learning maths and increasing physical activity in primary school students. 2020.

McIver KL, Pate RR, Dowda M, Johnson SB, Yang J, Butterworth M, et al. Cross-country comparisons of physical activity and sedentary behavior among 5-year-old children. 2020.

McLoughlin GM, Edwards CG, Jones A, Chojnacki MR, Baumgartner NW, Walk AD, et al. School lunch timing and children's physical activity during recess: An exploratory study. 2019.

Howells K, Coppinger T. et al. Children's perceived and actual physical activity levels within the elementary school setting. 2021.

Peacock J, Bowling A, Finn K, McInnis K. et al. Use of outdoor education to increase physical activity and science learning among low-income children from urban schools. 2021.

Moghaddaszadeh A, Belcastro AN. Guided active play promotes physical activity and improves fundamental motor skills for school-aged children. 2021.

Malik JA, Coto J, Pulgaron ER, et al. Sedentary behavior moderates the relationship between physical activity and cardiometabolic risk in young Latino children. 2021.

Grasten A, Huhtiniemi M, Kolunsarka I, Jaakkola T. et al. Developmental associations of accelerometer-measured moderate-to-vigorous physical activity and sedentary time with cardiorespiratory fitness in schoolchildren. 2022.

Tortella P, Haga M, Ingebrigtsen JE, Fumagalli GF, Sigmundsson H. et al. Comparing free play and partly structured play in 4-5-year-old children in an outdoor playground. 2019.

St Laurent CW, Burkart S, Alhassan S. et al. Feasibility, acceptability, and preliminary efficacy of a recess-based fitness intervention in elementary school children. 2019.

Wong MM, Brower KJ, Conroy DA, Craun EA. Convergence between the Child Behavior Checklist sleep items, actigraphy, and other sleep measures among children of parents with alcoholic disorders and controls. 2022.

So H-K, Chua GT, Yip K-M, et al. Impact of COVID-19 pandemic on school-aged children's physical activity, screen time, and sleep in Hong Kong: A cross-sectional repeated measures study. 2022

Ye S, Pope ZC, Lee JE, Gao Z. et al. Effects of school-based exergaming on urban children's physical activity and cardiorespiratory fitness: A quasi-experimental study. 2019

Siaplaouras J, Frerix M, Apitz A, Zoller D, Apitz C. et al. Effects of exercise training on heart rate variability in children and adolescents with pulmonary arterial hypertension: A pilot study. 2021

Siegel BI, Cakmak A, Reinertsen E, et al. Use of a wearable device to assess sleep and motor function in Duchenne muscular dystrophy. 2020.

Sung YS, Lin LY. Preliminary study on physical activity and motor performance in preschool children with autism spectrum disorder. 2019.

Taylor LG, Clark AF, Wilk P, Button BL, Gilliland JA. Exploring the effect of perceptions on children's physical activity in varying geographic contexts: Using a structural equation modelling approach to examine a cross-sectional dataset. 2018.

Swindell N, Wachira L-J, Okoth V, et al. Prevalence and correlates of compliance with 24-h movement guidelines among children from urban and rural Kenya—The Kenya-LINX project. 2022.

Tarp J, Bugge A, Andersen LB, et al. Does adiposity mediate the relationship between physical activity and biological risk factors in youth?: A cross-sectional study from the International Children's Accelerometry Database (ICAD). 2018.

Tobe RH, MacKay-Brandt A, Lim R, et al. A longitudinal resource for studying connectome development and its psychiatric associations during childhood. 2021.

Taylor SL, Noonan RJ, Knowles ZR, McGrane B, Curry WB, Fairclough SJ. Acceptability and feasibility of single-component primary school physical activity interventions to inform the AS:Sk Project. 2018.

Volmut T, Pisot R, Planinsec J, Simunic B. et al. Physical activity drops during summer holidays for 6- to 9-year-old children. 2020.

Sit CHP, Huang WY, Yu JJ, McKenzie TL. Accelerometer-assessed physical activity and sedentary time at school for children with disabilities: Seasonal variation. [Year Missing]

Strizich G, Kaplan RC, Sotres-Alvarez D, et al. Objectively measured sedentary behavior, physical activity, and cardiometabolic risk in Hispanic youth: Hispanic Community Health Study/Study of Latino Youth. 2018.

Tokarek NR, Cho CC, Strath SJ, Swartz AM. The impact of stand-biased desks on afterschool physical activity behaviors of elementary school children. 2022.

van den Heuvel MM, Oei EHG, Renkens JJM, Bierma-Zeinstra SMA, van Middelkoop M. et al. Structural spinal abnormalities on MRI and associations with weight status in a general pediatric population. 2020.

Gammon C, Atkin AJ, Corder K, et al. Influence of guideline operationalization on youth activity prevalence in the International Children’s Accelerometry Database. 2022.

Ariz U, Fernandez-Atutxa A, Ruiz-Litago F. et al. Physical activity at school recess: A key element in balancing social disparities. 2022.

Zhou M, Lalani C, Robinson TN. Sleep duration, timing, variability, and measures of adiposity among 8- to 12-year-old children with obesity. 2018.

Brazendale K, Beets MW, van Sluijs EMF. Children's moderate-to-vigorous physical activity on weekdays versus weekend days: A multi-country analysis. 2021.

Spanou M, Kaioglou V, Venetsanou F. et al. "Move" their brain: Motor competence mediates the relationship of physical activity and executive functions in children. 2022.

Heemskerk CHHM, Sumatic M, Malmberg LE. Individual differences in the effects of physical activity on classroom behaviour. 2022.

Migueles JH, Martinez-Nicolas A, Ortega FB. Activity-rest circadian pattern and academic achievement, executive function, and intelligence in children with obesity. 2021.

Al Backer NB, Alzawad M, Bashir S. et al. The relationship between sleep and cognitive performance in autism spectrum disorder (ASD): A pilot study. 2018.

Wen CKF, Liao Y, Dunton GF. Relationships among affective states, physical activity, and sedentary behavior in children: Moderation by perceived stress. 2018.

Guimaraes RD, Mathieu ME, Barnett TA. Physical activity, screen time, and sleep trajectories from childhood to adolescence: The influence of sex and body weight status. 2021.

Ganzar LA, Salvo D, Hoelscher DM. Longitudinal changes in objectively measured physical activity and sedentary time among school-age children in Central Texas, US during the COVID-19 pandemic. 2022.

Schmidt MD, Rathbun SL, Ahn SJ. Agreement between Fitbit and ActiGraph estimates of physical activity in young children. 2023.

Mayorga-Vega D, Casado-Robles C, Viciana J. et al. Validity of activity wristbands for estimating daily physical activity in primary schoolchildren under free-living conditions: School-Fit study. 2023.

Burchartz A, Kolb S, Woll A. et al. How specific combinations of epoch length, non-wear time, and cut-points influence physical activity: Processing accelerometer data from children and adolescents in the nationwide MoMo study. 2023.

Haapala EA, Rantalainen T, Duckham RL. Accelerometer-based osteogenic indices, moderate-to-vigorous and vigorous physical activity, and bone traits in adolescents. 2022.

Alves JM, Chow T, Page KA. Associations between sleep and metabolic outcomes in preadolescent children. 2022.

Medrano M, Cadenas-Sanchez C, Labayen I. et al. Associations of fitness and physical activity with specific abdominal fat depots in children with overweight/obesity. 2022.

Haapala EA, Gao Y, Finni T. et al. Validity of traditional physical activity intensity calibration methods and the feasibility of self-paced walking and running on individualised calibration of physical activity intensity in children. 2020.

Hoffmann B, Kobel S, Steinacker JM. High sedentary time in children is not only due to screen media use: A cross-sectional study. 2019.

Agar G, Oliver C, Richards C. et al. Direct assessment of overnight parent-child proximity in children with behavioral insomnia: Extending models of operant and classical conditioning. 2022.

Alves JM, Chow T, Page KA. Associations between sleep and metabolic outcomes in preadolescent children. 2022.

Wyszynska J, Matlosz P, Herbert J. et al. The association of actigraphic sleep measures and physical activity with excess weight and adiposity in kindergarteners. 2021.

Kahn M, Schnabel O, Sadeh A. et al. Sleep, screen time, and behaviour problems in preschool children: An actigraphy study. 2021.

Smith C, Galland B, Meredith-Jones K. et al. ActiGraph GT3X+ and Actical wrist and hip-worn accelerometers for sleep and wake indices in young children using an automated algorithm: Validation with polysomnography. 2020.

Zhou M, Lalani C, Robinson TN. Sleep duration, timing, variability, and measures of adiposity among 8- to 12-year-old children with obesity. 2018.

Pesola AJ, Esmaeilzadeh S, Rinne T. et al. Sensitivity and specificity of measuring children's free-living cycling with a thigh-worn Fibion® accelerometer. 2023.

Bolling M, Mygind E, Elsborg P. et al. The association between education outside the classroom and physical activity: Differences attributable to the type of space? 2021.

Villalba-Heredia L, Rodriguez C, Mendez-Gimenez A. et al. A cross-sectional study to measure physical activity with accelerometry in ADHD children according to presentations. 2023.

Hikihara Y, Watanabe M, Tanaka S. et al. Does earlier acquisition of motor competence promote pubertal physical activity in Japanese elementary school children: A 4-year follow-up study. 2022.

dos Santos MAM, Nevill AM, Maia JAR. Modeling children's development in gross motor coordination reveals key modifiable determinants: An allometric approach. 2018.

Schwenke P, Coenen M. et al. Influence of sit-stand tables in classrooms on children's sedentary behavior and teacher's acceptance and feasibility: A mixed-methods study. 2022.

Verswijveren SJJM, Lamb KE, Ridgers ND. Cross-sectional associations of total daily volume and activity patterns across the activity spectrum with cardiometabolic risk factors in children and adolescents. 2020.

Bartelink N, van Assema P, Jansen M, Savelberg H, Kremers S. The moderating role of the school context on the effects of the Healthy Primary School of the Future. 2019.

Smith C, Galland B, Taylor R, Meredith-Jones K. ActiGraph GT3X+ and Actical wrist and hip-worn accelerometers for sleep and wake indices in young children using an automated algorithm: Validation with polysomnography. 2020

Jindal I, Puyau M, Adolph A, Butte N, Musaad S, Bacha F. The relationship of sleep duration and quality to energy expenditure and physical activity in children. 2021

Altenburg TM, Wang XH, Chinapaw MJM. The consequences of using different epoch lengths on the classification of accelerometer-based sedentary behaviour and physical activity. 2021.

Smith E, Fazeli F, Clark CCT. Physical behaviors and fundamental movement skills in British and Iranian children: An isotemporal substitution analysis. 2021.

Hooke MC, Hoelscher A, Mathiason MA. Kids are moving: A physical activity program for children with cancer. 2019.

Mooses K, Oja M, Kull M. et al. Validating Fitbit Zip for monitoring physical activity of children in school: A cross-sectional study. 2018.

Grasten A, Yli-Piipari S, Jaakkola T. et al. Predicting accelerometer-based physical activity in physical education and total physical activity: The Self-Determination Theory approach. 2019.

Ji MM, Tang A, Lin Q. et al. The relationship between obesity, sleep, and physical activity in Chinese preschool children. 2018.

Estrada. Stress, physical activity, and sedentary behavior in minority girls. 2023.

Lin YY, Borghese MM, Janssen I. et al. Bi-directional association between sleep and outdoor active play among 10-13-year-olds. 2018.

Barnett TA, Contreras G, Henderson M. et al. Identifying risk profiles for excess sedentary behavior in youth using individual, family, and neighborhood characteristics. 2021.

Judice PB, Magalhaes JP, Sardinha RLB. Sensor-based physical activity, sedentary time, and reported cell phone screen time: A hierarchy of correlates in youth. 2021.

Brudy L, Meyer M, Muller J. et al. Move more - be happier? Physical activity and health-related quality of life in children with congenital heart disease. 2021.

Willinger L, Brudy L, Muller J. et al. Association between objectively measured physical activity and arterial stiffness in children with congenital heart disease. 2021.

Broad AA, et al. Classroom activity breaks improve on-task behavior and physical activity levels regardless of time of day. 2021.

Silva ECM. Two-year effectiveness of a controlled trial with physically active lessons on behavioral indicators of school children. 2020.

Wick K, et al. I can stand learning: A controlled pilot intervention study on the effects of increased standing time on cognitive function in primary school children. 2018.

Pagels P. et al. Pupils' use of school outdoor play settings across seasons and its relation to sun exposure and physical activity. 2020.

Sales D, et al. Association between school environment with sedentary behavior and physical activity intensity in children. 2023.

James ME, King-Dowling S, Cairney J. et al. Effects of comorbid developmental coordination disorder and symptoms of attention deficit hyperactivity disorder on physical activity in children aged 4-5 years. 2021.

Sedumedi CM, Janssen X, Monyeki MA. Association between objectively determined physical activity levels and body composition in 6-8-year-old children from a Black South African population: BC-IT study. 2021.

Migueles JH, Nystroem CD, Lof M. et al. Longitudinal associations of movement behaviors with body composition and physical fitness from 4 to 9 years of age: Structural equation and mediation analysis with compositional data. 2023.

McGee M, Unger S, O'Connor DL. Lean mass accretion in children born very low birth weight is significantly associated with estimated changes from sedentary time to light physical activity. 2020.

Watson A, Dumuid D, Olds T. et al. Associations between 24-hour time use and academic achievement in Australian primary school-aged children. 2020.

Burchartz A, Oriwol D, Woll A. et al. Comparison of self-reported and device-based measured physical activity among children in Germany. 2021.

Arregi A, Lertxundi A, Lertxundi N. et al. Environmental noise exposure and sleep habits among children in a cohort from Northern Spain. 2022.

Kokkonen JM, Vepsaelaeinen H, Ray C. et al. Associations between parent-child nature visits and sleep, physical activity, and weight status among Finnish 3-6-year-olds. 2021.

Fairclough SJ, Rowlands AV, Boddy LM. Cut-point-free accelerometer metrics to assess children's physical activity: An example using the school day. 2020.

Gerber M, Lang C, Walter C. et al. Physical activity, sedentary behavior, weight status, and body composition among South African primary schoolchildren. 2022.

Kunicki ZJ, Kattelmann KK, White AA. Dyadic analysis of a self-report physical activity measure for adult-youth dyads. 2022.

Tarp J, Child A, Brage S. et al. Physical activity intensity, bout-duration, and cardiometabolic risk markers in children and adolescents. 2018.

Williams G, Aggio D, Smith L. et al. Physical activity levels in children with sensory problems: Cross-sectional analyses from the Millennium Cohort Study. 2018.

Steene-Johannessen J, Hansen BH, Ekelund U. et al. Variations in accelerometry-measured physical activity and sedentary time across Europe - Harmonized analyses of 47,497 children and adolescents. 2020.

Judice PB, Hetherington-Rauth M, Northstone,K. Changes in Physical Activity and Sedentary Patterns on Cardiometabolic Outcomes in the Transition to Adolescence: International Children's Accelerometry Database 2.0. 2020

Jaakkola T, Yli-Piipari S, Huhtiniemi M. et al. Longitudinal associations among cardiorespiratory and muscular fitness, motor competence, and objectively measured physical activity. 2019.

**No accelerometer**

Koohsari MJ, Oka K, Shibata A, et al. Correlates of domain-specific sedentary behaviors and objectively assessed sedentary time among elementary school children. 2022.

Kubik MY, Fulkerson JA, Sirard JR, et al. School-based secondary prevention of overweight and obesity among 8- to 12-year old children: Design and sample characteristics of the SNAPSHOT trial. 2018.

Diaz A, Perez S, Lopez DM. Adaptation component based on wearable technology to support personalized tracking of physical activity in children. 2019.

Dudley DA, Cotton WG, Peralta LR, Winslade M. et al. Playground activities and gender variation in objectively measured physical activity intensity in Australian primary school children: a repeated measures study. 2018.

Sharaievska I, Battista RA, Zwetsloot J. et al. Use of physical activity monitoring devices by families in rural communities: qualitative approach. 2019.

Abdelkarim O, Fritsch J, Jekauc D, Bos K. et al. Examination of construct validity and criterion-related validity of the German Motor Test in Egyptian schoolchildren. 2021.

**Adherence data unclear**

Antczak D, Lonsdale C, del Pozo Cruz B, et al. Reliability of GENEActiv accelerometers to estimate sleep, physical activity, and sedentary time in children. 2021.

Bellamy J, Broderick C, Hardy LL, et al. Feasibility of a school-based exercise intervention for children with intellectual disability to reduce cardiometabolic risk. 2020.

Bloemen MA, Takken T, de Groot JF, et al. Determinants of physical activity in young wheelchair-users with spina bifida. 2020.

Boddy LM, Noonan RJ, Kim Y, et al. Comparability of children's sedentary time estimates derived from wrist worn GENEActiv and hip worn ActiGraph accelerometer thresholds. 2018.

Boddy LM, Noonan RJ, Rowlands AV, et al. The backwards comparability of wrist worn GENEActiv and waist worn ActiGraph accelerometer estimates of sedentary time in children. 2019.

Adab P, Barrett T, Bhopal R, et al. The West Midlands ActiVe lifestyle and healthy Eating in School children (WAVES) study: a cluster randomised controlled trial testing the clinical effectiveness and cost-effectiveness of a multifaceted obesity prevention intervention programme targeted at children aged 6-7 years. 2018.

Covington L, Satti A, Brewer B, et al. Concordance in caregiver and child sleep health metrics among families experiencing socioeconomic disadvantage: A pilot study. 2022.

Gaba A, Pedisic Z, Stefelova N, et al. Sedentary behavior patterns and adiposity in children: A study based on compositional data analysis. 2020.

Premelc J, Meh K, Vaha-Ypya H, et al. Do fitter children better assess their physical activity with questionnaire than less fit children? 2022.

Kerr C, Smith L, Charman S, et al. Physical education contributes to total physical activity levels and predominantly in higher intensity physical activity categories. 2018.

Jarvamagi M, Riso E-M, Reisberg K, Jurimae J. et al. Development of cardiorespiratory fitness in children in the transition from kindergarten to basic school according to participation in organized sports. 2022.

El-Banna G, Baskar D, Segovia N, Frick S. et al. Clubfoot activity and recurrence exercise study (CARES). 2022.

Bernal C, Lhuisset L, Bru N, Fabre N, Bois J. et al. Effects of an intervention to promote physical activity and reduce sedentary time in disadvantaged children: Randomized trial. 2021.

Witcraft SM, Wickenhauser ME, Russell KM, et al. Do anxiety and mood vary among disparate sleep profiles in youth with craniopharyngioma? A latent profile analysis. 2022.

Wyatt K, Lloyd J, Creanor S, et al. Cluster randomised controlled trial and economic and process evaluation to determine the effectiveness and cost-effectiveness of a novel intervention [Healthy Lifestyles Programme (HeLP)] to prevent obesity in school children. 2018.

Yang C-H, Huh J, Mason TB, et al. Mother-child dyadic influences of affect on everyday movement behaviors: Evidence from an ecological momentary assessment study 2020

Sidol C. et al. Evaluating the effect of poor sleep efficiency and next-day behavior among children with attention deficit/hyperactivity disorder. Adherence data unclear. 2020

Kolunsarka I, Grasten A, Jaakkola T. et al. Development of children's actual and perceived motor competence, cardiorespiratory fitness, physical activity, and BMI. 2021.

**Adherence data non discernible**

Santaliestra-Pasias AM, Dios JEL, Sprengeler O, Hebestreit A, De Henauw S, Eiben G, et al. Food and beverage intakes according to physical activity levels in European children: the IDEFICS (Identification and prevention of Dietary and lifestyle induced health EFfects In Children and infantS) study. 2018.

Larson JN. Fit 'n' cool kids: Children's experiences and the effects of character peer modeling and goal setting on children's physical activity and fruit and vegetable consumption. 2019.

Clementi MA. Efficacy of an integrated sleep and anxiety intervention for anxious children: A pilot randomized controlled trial. 2019.

Solomon-Moore E, Toumpakari Z, Sebire SJ, Thompson JL, Lawlor DA, Jago R. et al. Roles of mothers and fathers in supporting child physical activity: A cross-sectional mixed-methods study. 2018.

Barnett LM, Lubans DR, Timperio A, Salmon J, Ridgers ND. What is the contribution of actual motor skill, fitness, and physical activity to children's self-perception of motor competence? 2018.

Adams J, Veitch J, Barnett L. et al. Physical activity and fundamental motor skill performance of 5-10 year old children in three different playgrounds. 2018.

Adank AM, Van Kann DHH, Hoeboer JJAA, de Vries SI, Kremers SPJ, Vos SB. Investigating motor competence in association with sedentary behavior and physical activity in 7-to 11-year-old children. 2018.

Cabanas-Sanchez V, Martinez-Gomez D, Esteban-Cornejo I, Castro-Pinero J, Conde-Caveda J, Veiga OL. Reliability and validity of the Youth Leisure-time Sedentary Behavior Questionnaire (YLSBQ). 2018.

Cadenas-Sanchez CC, Migueles JH, Verdejo-Roman J, Erickson KI, Esteban-Cornejo I, Catena A, et al. Physical activity, sedentary time and fitness in relation to brain shapes in children with overweight/obesity: links to intelligence. 2022.

Do B, Mason TB, Yi L, Yang C-H, Dunton GF. Momentary associations between stress and physical activity among children using ecological momentary assessment. 2021.

Estevan I, Clark C, Molina-Garcia J, Menescardi C, Barton V, Queralt A. et al. Longitudinal association of movement behaviour and motor competence in childhood: A structural equation model, compositional, and isotemporal substitution analysis. 2022.

Clementi MA, Alfano CA. An integrated sleep and anxiety intervention for anxious children: A pilot randomized controlled trial. 2020.

Garcia-Hermoso A, Hormazabal-Aguayo I, Gonzalez-Calderon N, Russell-Guzman J, Vicencio-Rojas F, Chacana-Canas C, et al. Exercise program and blood pressure in children: The moderating role of sedentary time. 2020.

Kerfeld CI, Hurvitz PM, Bjornson KF. Physical activity measurement in children who use mobility assistive devices: Accelerometry and global positioning system. 2021.

Koopman-Verhoeff ME, Bolhuis K, Cecil CAM, Kocevska D, Hudziak JJ, Hillegers MHJ, et al. During day and night: Childhood psychotic experiences and objective and subjective sleep problems. 2019.

Gråstén A, Yli-Piipari S, Huhtiniemi M, Salin K, Hakonen H, Jaakkola T. et al. A one-year follow-up of basic psychological need satisfactions in physical education and associated in-class and total physical activity. 2021.

Lubasch JS, Thumann B, Bucksch J, Brackmann LK, Wirsik N, Donnelly A, et al. School- and leisure time factors are associated with sitting time of German and Irish children and adolescents during school: Results of a DEDIPAC feasibility study. 2020.

Marin-Puyalto J, Maestu J, Gomez-Cabello A, Latt E, Remmel L, Purge P, et al. Vigorous physical activity patterns affect bone growth during early puberty in boys. 2018.

Masini A, Marini S, Leoni E, Lorusso G, Toselli S, Tessari A, et al. Active breaks: A pilot and feasibility study to evaluate the effectiveness of physical activity levels in a school based intervention in an Italian primary school. 2020.

Rubin L, Gaba A, Pelclova J, Stefelova N, Jakubec L, Dygryn J, et al. Changes in sedentary behavior patterns during the transition from childhood to adolescence and their association with adiposity: A prospective study based on compositional data analysis. 2022.

Schoeppe S, Salmon J, Williams S, Power D, Waters K, Alley S, et al. Feasibility of using activity trackers and apps to increase physical activity in whole families: The Step it Up Family intervention. 2022.

Schoeppe S, Salmon J, Williams SL, Power D, Alley S, Rebar AL, et al. Effects of an activity tracker and app intervention to increase physical activity in whole families-the Step it Up Family feasibility study. 2020.

Vlaar J, Brussoni M, Janssen I, Masse LC. Roaming the neighbourhood: Influences of independent mobility parenting practices and parental perceived environment on children's territorial range. 2019.

Wang H, Blake H, Chattopadhyay K. et al. School-based behaviour change intervention to increase physical activity levels among children: A feasibility cluster non-randomised controlled trial in Yangzhou, China. 2021.

Manasse SM, Haedt-Matt AA, Goldschmidt AB. The moderating role of sleep duration on momentary relations between negative affect and loss-of-control eating in children and adolescents. 2022.

Veijalainen A, Haapala EA, Lakka TA. Associations of physical activity, sedentary time, and cardiorespiratory fitness with heart rate variability in 6-to 9-year-old children: the PANIC study. 2022.

Bartholomew JB. Individual- and environmental-level predictors of recess activity and sedentary behavior: Findings from the I-CAN! Study. 2022.

Constable AM, Porter JE, Lakka TA. The positive relationship between moderate-to-vigorous physical activity and bone mineral content is not mediated by free leptin index in prepubertal children: The PANIC study. 2021.

McQuillan ME, Bates JE, Honaker SM. Children's sleep and externalizing problems: A day-to-day multilevel modeling approach. 2022.

Medd ER, Beauchamp MR, Rhodes RE. Family-based habit intervention to promote parent support for child physical activity in Canada: Protocol for a randomized trial. 2020.

Have M, Nielsen JH, Kristensen PL. Classroom-based physical activity improves children’s math achievement – A randomized controlled trial. 2018.

Lopez NV, Yang CH, Belcher BR, Margolin G, Dunton GF. Within-subject associations of maternal physical activity parenting practices on children's objectively measured moderate-to-vigorous physical activity. 2022.

Miatke A, Maher C, Olds T. et al. Are all MVPA minutes equal? Associations between MVPA characteristics, independent of duration, and childhood adiposity. 2021.

Migueles JH, Cadenas-Sanchez C, Ortega FB. Associations of objectively-assessed physical activity and sedentary time with hippocampal gray matter volume in children with overweight/obesity. 2020.

Lin YY, Tremblay MS, Chaput JP. Temporal and bi-directional associations between sleep duration and physical activity/sedentary time in children: An international comparison. 2018.

Gu XL, Tamplain PM, Wang J. et al. A mediation analysis of the association between fundamental motor skills and physical activity during middle childhood. 2021.

Callender LK, Borghese MM, Janssen I. et al. Which intensities, types, and patterns of movement behaviors are most strongly associated with cardiometabolic risk factors among children? 2021.

Verswijveren SJJM, Douglas B, Ridgers ND. Count- versus MAD-based accelerometry-assessed movement behaviors and associations with child adiposity and fitness. 2021.

Aadland E, Andersen LB, Resaland GK. A comparison of 10 accelerometer non-wear time criteria and logbooks in children. 2018.

Button BLG, Clark AF, Gilliland JA. Understanding factors associated with children achieving recommended amount of MVPA on weekdays and weekend days. 2020.

Foulkes JD, Knowles Z, Foweather L. et al. Is foundational movement skill competency important for keeping children physically active and at a healthy weight? 2022.

Li F, Yin L, Gao Z. et al. Examining relationships among Chinese preschool children's meeting 24-hour movement guidelines and fundamental movement skills. 2022.

Quan MH, Zhang HB, Chen PJ. Preschoolers' technology-assessed physical activity and cognitive function: A cross-sectional study. 2018.

**Abstract only**

Suresh S, Lindhiem O, Goel M. et al. Application of sensors and machine learning in the evaluation of hyperactivity in children. 2020.

Wagner K, Cripe L, Eagle M, et al. EP.83Design of a Phase 2/3 randomized controlled trial of suvodirsen (WVE-210201) in patients with Duchenne muscular dystrophy amenable to exon 51 skipping. 2019

Williams S, Mizen L. et al. Feasibility of undertaking at-home PSG and actigraphy upon children who have SYNGAP1-related intellectual disability. 2022.

Wong LS, Reilly JJ, McCrorie P, Harrington DM. Moderate-to-vigorous intensity physical activity during school hours in a representative sample of 10-11-year-olds in Scotland. 2022.

Watson AJL, Timperio A, Brown H, Hesketh KD. A pilot primary school active break program (ACTI-BREAK): Effects on academic and physical activity outcomes for students in Years 3 and 4. 2019.

Adams J, Barnett L, Veitch J. et al. What sort of playground design facilitates physical activity and encourages children to use diverse motor skills? 2018.

Albizu-Jacob A, Loecher N, Gray H, Stern M. et al. Engaging parents of pediatric cancer survivors with obesity in a healthy lifestyle intervention during the Covid-19 pandemic: Implications for trial recruitment and data collection. 2021.

Barnett E. et al. Physical activity and enjoyment: Measurement, evaluation, and theory. 2021.

Bastien L, Theoret R, Godbout R. et al. Circadian preference, social jetlag, and socio-emotional functioning of gifted children. 2020.

Batson T, Singh H, Zhang C, Colquitt G, Modlesky C. et al. Reliability of a progressive lateral step-up test and its relationship with physical activity in children with cerebral palsy. 2020.

Beani E, De Cavalieri M, Filogna S, et al. Wearable sensors for detecting upper limb use during daily life in children with unilateral cerebral palsy. 2022.

Bendixen R, Hartman A, Little N, Feltman M. P. et al.199 Accelerometer prediction equations in boys with Duchenne muscular dystrophy: Importance of disease-specific equations. 2019.

Cassim R, Dharmage S, Milanzi E, et al. Is asthma longitudinally related to physical activity in early childhood? 2019.

Fals AM. Effective multidisciplinary approach to childhood obesity prevention and treatment: Integration of technology, health coaching of child, and fitness options. 2019.

Faulkner MS, Michaliszyn SM. Engaging Hispanic adolescents with type 2 diabetes or obesity in personalized exercise. 2018.

Fitzgerald T, Cameron K, Albesher R, et al. Associations between physical activity and motor competence in preschool-age children born very preterm and at term. 2022.

Fitzgerald T, McGinley J, Cheong J, Doyle L, Spittle A. et al. Strength, motor competence, and physical activity in very preterm and term-born preschool-age children. 2019.

Fritch J, Blay R, Cole M, Krause C, Bilek L, Acquazzino M. et al. HEALTHY BONES HEALTHY LIFE: Effect of physical activity on bone health in pediatric cancer survivors. 2022.

Fukakusa B, Kuan M, Bennett E, Voss C, Harris K. et al. THE FEASIBILITY OF A VIRTUAL PHYSICAL ACTIVITY COUNSELLING INTERVENTION IN CHILDREN WITH CONGENITAL HEART DISEASE. 2022.

Fulkerson JA, Barr-Anderson DJ, Horning M, et al. Home food environment and child dietary and physical activity outcomes of the nu-home RCT. 2021.

Galbraith L, Bull K, Hill C. et al. A novel home video behavior analysis algorithm to diagnose childhood chronic insomnia. 2018.

Garcia ML, Castaneda SF, Gallo LC, et al. Socio-environmental influences on youth total sedentary time: Results from the Hispanic community health study/study of Latino youth. 2018.

Gardas S, Lysaght C, Gross Mcmillan A, et al. An accelerometry-based approach to quantify intensity of Hand Arm Bimanual Intensive Therapy (HABIT) and performance gains in children with unilateral cerebral palsy. 2022.

Goodhines P, Barker D, Gredvig-Ardito C, et al. CHARACTERIZING SLEEP REGULARITY FROM ACTIGRAPHY IN YOUNGER AND OLDER ADOLESCENTS. 2022.

Hanish A, Klein AJ, Mathews T, et al. Sleep smart in adolescents with neurodevelopmental disorders. 2021.

Hoyt C. et al. Accelerometry for the early detection of motor deficits in children. 2018.

Hoyt C, Sherman S, Brown S, et al. Using wearable technology to describe impact of disability among pediatric populations. 2019.

Griffiths LJ, Cortina-Borja M, Tingay K, et al. ARE ACTIVE BOYS AND GIRLS AT INCREASED RISK OF HOSPITAL ATTENDANCE OR ADMISSION FOR INJURY? A LONGITUDINAL STUDY IN WALES AND SCOTLAND USING LINKED COHORT AND ELECTRONIC HEALTH RECORDS. 2018.

Ha L, Mizrahi D, Johnston K, et al. Improving physical activity levels among childhood cancer survivors: A digital educational program. 2021.

Kirkpatrick B, Leader G. et al. Using assistive technology to support parents in the management of sleep problems experienced by children with autism spectrum disorder. 2018.

Koinis-Mitchell D. et al. Cultural and contextual mechanisms relevant to asthma and physical activity in urban children. 2018.

Koinis-Mitchell D, Kopel SJ, McQuaid EL, et al. Physical activity and asthma outcomes in urban children: Does sleep matter? 2022.

Koinis-Mitchell D, Kopel SJ, McQuaid EL, et al. Physical activity and asthma outcomes in urban children: Differences by ethnic group and weight status. 2019.

Konrad J, Marrus N, Lang C. et al. Using accelerometry to quantify motor traits of children with and without autism. 2021.

Copeland KA, Brown C, Percy Z, et al. Peer-led group sessions to improve healthful eating and physical activity in a low-income primary care clinic population. 2018.

Koopman-Verhoeff ME, Bolhuis K, Cecil CAM, et al. During day and night: Childhood psychotic-like experiences and nightmares. 2018.

Lavelle G, Noorkoiv M, Theis N, et al. Validity of the International Physical Activity Questionnaire Short Form (IPAQ-SF) as a measure of physical activity (PA) in young people with cerebral palsy: A cross-sectional study. 2020.

Leclerc JB, Chevrier E, Chicoine M, Godbout R. et al. Sleep characteristics of children with Tourette syndrome and explosive outbursts. 2019.

Lee J, Batson W, Wilhoite S, et al. Lower limb muscle quality is more strongly related to physical activity than functional strength in children with cerebral palsy. 2021.

Logue C, Flynn J, Gallagher A, et al. Get A Move On: Using intelligent personal systems to promote behavior change within the home setting - A process evaluation. 2020.

McFatrich M, Mann CM, Bennett AV, et al. Assessing how a child with cancer functions using patient-reported and objective physical activity data. 2019.

Meers JM, Bower JL, Alfano CA. Objective sleep and child resiliency in deployed compared to non-deployed military families. 2018.

Mitchell JA, Morales K, Williamson A, et al. Changes in childhood sleep patterns in an intervention study prior to and during COVID-19 restrictions. 2021.

Mitchell JA, Eck C, Hickey J, et al. Parent-child perceptions about healthy sleep promotion in a mobile health sleep extension intervention. 2020.

Moore B, Callahan M, Martin S, et al. Sedentary behavior and physical activity in children at high versus low risk for obesity. 2021.

Nezami B, Power J, Hurley L, Tate D. et al. A randomized controlled trial of two diet monitoring approaches in a mobile weight loss intervention. 2020.

Norgaard M, Herlin T. et al. Gender differences in physical fitness related to selection in club-sport activities in JIA. 2020.

Palmer C, Alfano CA, Weems CF, LaVoy E. et al. Childhood sleep patterns longitudinally predict later post-traumatic stress after Hurricane Harvey. 2019.

Preti PSA, Ambaglio C, Carnevale Pellino V, et al. Can an educational camp be useful to promote an active lifestyle in people with hemophilia? Study design of hemophilia educational physical activity (HEPA) camp. 2020.

Reedman S, Frampton K, Gomersall S, et al. Convergent validity of the Physical Literacy Assessment for Youth (PLAYfun) in ambulant children with cerebral palsy. 2022.

Reedman S, Johnson E, Sakzewski L, et al. Sedentary behavior peaks at 4-5 years of age in a longitudinal, population-based study of children with cerebral palsy followed between 1.5 and 12 years. 2019.

Reedman S, Trost S, Sakzewski L, Boyd R. et al. Environmental supports are associated with increased moderate-vigorous physical activity in children with cerebral palsy. 2022.

Reeves S, Poh BK, Cheah WL, et al. ToyBox Study Malaysia: A feasibility study to improve healthy energy balance and obesity-related behavior. 2020.

Williamson A, Wu K, Jawahar A, et al. Variation in sleep beliefs and behaviors among caregiver-child dyads participating in a sleep extension intervention. 2021.

Zarkogianni K, Athanasiou M, Mitsis K, et al. The Endorse pilot trial.

Zarrouf LR. The effect of extending total sleep time and weighted blankets on teenage swimmers' performance. 2020.

Sheth A, Yip H, Jaimini U, et al. Feasibility of recording sleep quality and sleep duration using Fitbit in children with asthma. 2018.

Silverman N, Beck M, Hall D, Hartman A, Bendixen R. et al. Using actigraphy for understanding activity and sleep patterns in young boys with Duchenne muscular dystrophy (DMD) during a clinical drug trial. 2020 AOTA Annual Conference & Expo.

Simpson EJ, Horstman AMH, Bawden SJ, et al. 13C-magnetic resonance spectroscopy; a viable technique to study overnight liver glycogen depletion and response to feeding in 8-12-year-old children. 2022

St Laurent C, Holmes J, Andre C, Spencer R. et al. Temporal and bidirectional associations between objectively measured physical activity and sleep in preschoolers. 2021

Torres-Lopez LV, Cadenas-Sanchez C, Migueles JH, et al. Associations of sleep-related outcomes with behavioral and emotional functioning in children with overweight/obesity. 2022.

Tuominen PPA, Raitanen J, Husu P, Kujala UM, Luoto RM. The effects of mothers' musical background on sedentary behavior, physical activity, and exercise adherence in their 5-6-year-old children using movement-to-music video program. 2018.

Verdone K, Mathew S. et al. Initial findings of the effects of early mobilization on inflammatory cytokines in critically ill pediatric patients: A pilot study. 2022.

Verswijveren SJJM, Lamb KE, Leech RM, et al. Activity accumulation and cardiometabolic risk in youth: A latent profile approach. 2020

Ward AL, Galland BC, Haszard JJ, et al. The effect of mild sleep deprivation on diet and eating behavior in children: Protocol for the Daily Rest, Eating, and Activity Monitoring (DREAM) randomized cross-over trial. 2019.

Schierloh U, Aguayo GA, Fichelle M, et al. Does predicted low suspend pump treatment improve control and quality of sleep in children with type 1 diabetes and their caregiver? The QUEST study. 2018.

Suk M, Park I, Kwon J. et al. Relationship between physical activity and physical fitness of children with cerebral palsy. 2019.

Sinisterra M, Hamburger S, Tully C, et al. Assessing sleep characteristics in young children with type 1 diabetes.

Ten Velde G, Plasqui G, Willeboordse M, et al. Feasibility and effect of the exergame BOOSTH introduced to improve physical activity and health in children: Protocol for a randomized controlled trial. 2020.

Fernandez-Rio J, Martinez AP. Increasing one school's physical activity levels through a self-regulated, multifactorial intervention during recess. 2021.

**Not in the age range**

Fiedler J, Seiferth C, Eckert T, Woll A, Wunsch K. et al. A just-in-time adaptive intervention to enhance physical activity in the SMARTFAMILY2.0 trial. 2022.

Loprinzi PD, Frith E. et al. Accelerometer-assessed physical activity and school absenteeism due to illness or injury among children and adolescents: NHANES 2003 to 2006. 2018.

Aittasalo M, Jussila AM, Tokola K, Sievanen H, Vaha-Ypya H, Vasankari T. et al. Kids Out; evaluation of a brief multimodal cluster randomized intervention integrated in health education lessons to increase physical activity and reduce sedentary behavior among eighth graders. 2019.

Robinson JC, Temple ML, Duck A, Klamm M. et al. Feasibility and effectiveness of two built environmental interventions on physical activity among 3-5-year-old preschoolers. 2019.

Pawlowski CS, Andersen HB, Schipperijn J. et al. Difference in outdoor time and physical activity during recess after schoolyard renewal for the least-active children. 2020.

Petersen TL, Brond JC, Kristensen PL, Aadland E, Grontved A, Jepsen R. et al. Resemblance in accelerometer-assessed physical activity in families with children: the Lolland-Falster Health Study. 2020.

Carson V, Zhang Z, Kuzik N, Adamo KB, Predy M, Crozier M, et al. The impact of new government childcare accreditation standards on children's in-care physical activity and sedentary time. 2022.

Eaton CK, Henning E, Lam J, Paasch V. et al. Actigraphy technology: Informing assessment and intervention for sleep disturbances in young children. 2019.

Emm-Collison LG, Sebire SJ, Salway R, Thompson JL, Jago R. et al. Multidimensional motivation for exercise: A latent profile and transition analysis. 2020.

Gerber C, Carcreff L, Paraschiv-Ionescu A, Armand S, Newman C. et al. How is daily performance associated with gross motor capacity in children with cerebral palsy? 2018.

Goodyear VA, Kerner C, Quennerstedt M. et al. Young people's uses of wearable healthy lifestyle technologies: surveillance, self-surveillance, and resistance. 2019.

Greenspan B. et al. Design and validation of wearable technologies for children with physical disabilities. 2020.

Haines J, Douglas S, Mirotta JA, O'Kane C, Breau R, Walton K, et al. Guelph Family Health Study: Pilot study of a home-based obesity prevention intervention. 2018.

Guseman EH, Sisson SB, Whipps J, Howe CA, Byra MM, Silver LE. Neighborhood and family characteristics associated with adiposity and physical activity engagement among preschoolers in a small rural community. 2022.

Rabbitts JA, Holley AL, Zhou C, Chen L. et al. Physical activity as a predictor of chronic pain following pediatric spinal surgery. 2021.

Razak LA, Yoong SL, Wiggers J, Morgan PJ, Jones J, Finch M, et al. Impact of scheduling multiple outdoor free-play periods in childcare on child moderate-to-vigorous physical activity: A cluster randomised trial. 2018.

Roscoe CMP, James RS, Duncan MJ. Accelerometer-based physical activity levels differ between week and weekend days in British preschool children. 2019.

Janzen L, Kuntze G, Toomey C, Condliffe E, Brunton L, Esau S, et al. Physical activity, adiposity, and function in youth with and without spastic cerebral palsy: A pilot cohort study. 2018.

Kalajas-Tilga H, Koka A, Hein V, Tilga H, Raudsepp L. et al. Motivational processes in physical education and objectively measured physical activity among adolescents. 2020.

Kang AW, Gans KM, Minkel J, Risica PM. Correlates of objectively measured sleep and physical activity among Latinx 3-to-5-year-old children. 2021.

Kudchadkar SR, Alsafi E, Melvin P. et al. Day-night activity rhythms in children admitted to the pediatric ICU for non-surgical diagnoses. 2018.

Lin YY, Lee WT, Yang HL, Weng WC, Lee CC, Jeng SF, et al. Screen time exposure and altered sleep in young children with epilepsy. 2020.

Lunt L, Shoop-Worrall S, Khanom S, Bolger I, Fantana AL, Short MA, et al. What do young people think about continuous data collection in clinical research and the types of electronic devices? 2019.

Mazzucca SL. Physical activity and sedentary behavior in early care and education centers: Identifying opportunities and testing strategies to support active classroom environments. 2018.

Mameli C, Brunetti D, Colombo V, Bedogni G, Schneider L, Penagini F, et al. Combined use of a wristband and a smartphone to reduce body weight in obese children: Randomized controlled trial. 2018.

Delisle Nystrom C, Alexandrou C, Henstrom M, Nilsson E, Okely AD, Wehbe El Masri S, et al. International study of movement behaviors in the early years (SUNRISE): Results from SUNRISE Sweden's pilot and COVID-19 study. 2020.

Fisher M, Wiseman-Hakes C, Obeid J, DeMatteo C. et al. Does sleep quality influence recovery outcomes after postconcussive injury in children and adolescents? 2022.

Fisher M, Wiseman-Hakes C, Obeid J, DeMatteo C. et al. Examining the trajectory and predictors of post-concussion sleep quality in children and adolescents. 2022.

Malden S, Reilly J, Gibson A, Bardid F, De Craemer M, Androutsos O, et al. Feasibility of the ToyBox-Scotland obesity prevention intervention in preschools: Results of a cluster randomised controlled trial. 2019.

Solomon-Moore E, Jago R, Beasant L, Brigden A, Crawley E. et al. Physical activity patterns among children and adolescents with mild-to-moderate chronic fatigue syndrome/myalgic encephalomyelitis. 2019

Romar J-E, Enqvist I, Kulmala J, Kallio J, Tammelin T. et al. Physical activity and sedentary behaviour during outdoor learning and traditional indoor school days among Finnish primary school students. 2019.

Rhodes RE, Quinlan A, Naylor PJ, Warburton DER, Blanchard CM. Predicting personal physical activity of parents during participation in a family intervention targeting their children. 2020.

Schmidt EM, Hoffman JA, Mule C, Briesch AM. Effects of a teacher training program to promote physically active play among preschoolers with autism spectrum disorders. 2021.

Ziesenitz VC, Lambrechtse P, Atkinson A, Bos EJ, Welzel T, Gilgen Y, et al. Feasibility of monitoring physical activity in children using commercial activity trackers. 2018.

Skau Pawlowski C, Bondo Andersen H, Schipperijn J. et al. Difference in outdoor time and physical activity during recess after schoolyard renewal for the least-active children. 2020

Rhodes RE, et al. Predicting personal physical activity of parents during participation in a family intervention targeting their children. 2020.

Hurter L, McNarry M, Stratton G, Mackintosh K. et al. Back to school after lockdown: The effect of COVID-19 restrictions on children's device-based physical activity metrics. J Sport Health Sci. 2022

Eijkemans M, Mommers M, Thijs C. et al. Physical activity and asthma development in childhood: Prospective birth cohort study. 2020.

Vanhala A, Haapala EA, Sääkslahti A, Hakkarainen A, Widlund A, Aunio P. et al. Associations between physical activity, motor skills, executive functions, and early numeracy in preschoolers. 2022.

Rosiek MA, Etnier JL, Willoughby MT. A comparison of the effects of outdoor physical activity and indoor classroom-based activities on measures of executive function in preschoolers. 2022.

Healy S, Brewer B, Garcia J, Daly J, Patterson F. et al. Sweat, sit, sleep: A compositional analysis of 24-hr movement behaviors and body mass index among children with autism spectrum disorder. 2021

Harrington DM, Davies MJ, Bodicoat DH, et al. Effectiveness of the 'Girls Active' school-based physical activity programme: A cluster randomised controlled trial. 2018

Sahlberg L, Lapinleimu H, Virtanen I. et al. Normative values for sleep parameters in preschoolers using actigraphy. 2018.

Button BLG. Measuring temporal differences in rural Canadian children's moderate-to-vigorous physical activity. 2020.

Kang AW, Gans KM, Risica PM. Physical activity levels among preschool-aged children in family child care homes: A comparison between Hispanic and non-Hispanic children. 2021.

Fossdal TS. Oh oobe doo, I wanna be like you: Associations between physical activity of preschool staff and preschool children. 2018.

Legnani RFS, Legnani E, Campos W. et al. Validation of a web questionnaire on physical activity for children and adolescents. 2022.

Hartman AG, McKendry S, Bendixen R. et al. Comparing contributors of parental sleep health in families with and without a child with Duchenne muscular dystrophy. 2022.

Costa RM, et al. Effect of a school-based multicomponent intervention on time-segmented physical activity and sedentary behavior among Brazilian adolescents: The Movimente Study. 2023.

Yang C-H, Wang S, Wang W-L, Belcher BR, Dunton GF. Day-level associations of physical activity and sedentary time in mother-child dyads across three years: A multi-wave longitudinal study using accelerometers. 2022

Taylor RW, Gray AR, Heath ALM, Galland BC, Lawrence J, Sayers R, et al. Sleep, nutrition, and physical activity interventions to prevent obesity in infancy: Follow-up of the Prevention of Overweight in Infancy (POI) randomized controlled trial at ages 3.5 and 5 years. 2018.

**Non-community setting**

Ghomrawi HM, Baumann LM, Kwon S, Hebal F, Hsiung G, Williams K, et al. Using accelerometers to characterize recovery after surgery in children. 2018.

Abadi MRH, Hase B, Dell C, Johnston JD, Kontulainen S. et al. Dog-assisted physical activity intervention in children with autism spectrum disorder: A feasibility and efficacy exploratory study. 2022.

Crawford S, Utt A, Beebe C, Armbruster R, Fisher A, Olney A, et al. Quality of sleep in a pediatric hospital: A descriptive study based on an assessment of interruptions, perceptions, and the environment. 2019.

Kudchadkar SR, Aljohani O, Johns J, Leroux A, Alsafi E, Jastaniah E, et al. Day-night activity in hospitalized children after major surgery: An analysis of 2271 hospital days. 2019.

Lin HP, Lynk N, Moore LL, Cabral HJ, Heffernan KS, Dumas AK, et al. A pragmatic approach to the comparison of wrist-based cutpoints of physical activity intensity for the MotionWatch8 accelerometer in children. 2020.

Rostami Haji Abadi M, Hase B, Dell C, Johnston JD, Kontulainen S. et al. Dog-assisted physical activity intervention in children with autism spectrum disorder: A feasibility and efficacy exploratory study. 2022.

Wulterkens BM, Fonseca P, Hermans LWA, Ross M, Cerny A, Anderer P, et al. It is all in the wrist: Wearable sleep staging in a clinical population versus reference polysomnography. 2021

Okely AD, Batterham MJ, Van Loo CMT, et al. Wrist acceleration cut points for moderate-to-vigorous physical activity in youth. 2018.

Byun W, Lee JM, Brusseau TA, et al. Classification accuracy of a wearable activity tracker for assessing sedentary behavior and physical activity in 3- to 5-year-old children. 2018.

Godino JG. Performance of a commercial multi-sensor wearable (Fitbit Charge HR) in measuring physical activity and sleep in healthy children. 2020.

**Protocol only**

Vaipuna TFW, Williams SM, Farmer VL, Meredith-Jones KA, Richards R, Galland BC, et al. Sleep patterns in children differ by ethnicity: Cross-sectional and longitudinal analyses using actigraphy. 2018.

van Delden AEQ, Band GPH, Slaets JPJ. A good beginning: Study protocol for a group-randomized trial to investigate the effects of sit-to-stand desks on academic performance and sedentary time in primary education. 2020.

Bai S, Goudie A, Borsheim E, Weber JL. The Arkansas Active Kids Study: Identifying contributing factors to metabolic health and obesity status in prepubertal school-age children. 2021.

Gerber M, Ayekoé SA, Beckmann J, Bonfoh B, Coulibaly JT, Daouda D, et al. Effects of school-based physical activity and multi-micronutrient supplementation intervention on growth, health, and well-being of schoolchildren in three African countries: The KaziAfya cluster randomized controlled trial protocol with a 2×2 factorial design. 2020.

Malden S, Hughes AR, Gibson AM, Bardid F, Androutsos O, De Craemer M, et al. Adapting the ToyBox obesity prevention intervention for use in Scottish preschools: Protocol for a feasibility cluster randomized controlled trial. 2018.

**Review**

Kracht CL, Staiano AE. Thinking inside the box: The future of young children's physical activity and the home environment. 2022.

**Duplicate dataset**

Lambrechtse PIP, Ziesenitz VC, Atkinson A, Bos EJ, Welzel T, Gilgen Y, et al. Monitoring the recovery time of children after elective tonsillectomies using commercial activity trackers: A prospective feasibility study. 2019.

Bringolf-Isler B, Schindler C, de Hoogh K, Kayser B, Suggs LS, Dossegger A, et al. Association of objectively measured and perceived environment with accelerometer-based physical activity and cycling: A Swiss population-based cross-sectional study of children. 2019.

Rowlands AV, Sherar LB, Fairclough SJ, Yates T, Edwardson CL, Harrington DM, et al. A data-driven, meaningful, easy-to-interpret, standardized accelerometer outcome variable for global surveillance. 2019.

Umstattd Meyer MR, Bridges Hamilton CN, Prochnow T, McClendon ME, Arnold KT, Wilkins E, et al. Come together, play, be active: Physical activity engagement of school-age children at Play Streets in four diverse rural communities in the U.S. 2019.

Mack I, Juchler N, Rey S, Hirsch S, Hoelz B, Eckstein J, et al. Prevalence and correlates of objectively measured weight status among urban and rural Mozambican primary schoolchildren: A cross-sectional study. 2022.

Wilkie HJ, Standage M, Gillison FB, Cumming SP, Katzmarzyk PT. Correlates of intensity-specific physical activity in children aged 9-11 years: A multilevel analysis of UK data from the International Study of Childhood Obesity, Lifestyle and the Environment. 2018.

Dumuid D, Wake M, Clifford S, Burgner D, Carlin JB, Mensah FK, et al. The association of the body composition of children with 24-hour activity composition. 2019.

Osborn W, Simm P, Olds T, Lycett K, Mensah FK, Muller J, et al. Bone health, activity, and sedentariness at age 11-12 years: Cross-sectional Australian population-derived study. 2018.

Geraci M. et al. Additive quantile regression for clustered data with an application to children's physical activity. 2019.

Clemes SA, Bingham DD, Pearson N, Chen YL, Edwardson C, McEachan R, et al. Sit-stand desks to reduce sedentary behaviour in 9- to 10-year-olds: The Stand Out in Class pilot cluster RCT. 2020.

Esbensen AJ, Hoffman EK, Beebe DW, Byars K, Carle AC, Epstein JN, et al. Randomized behavioral sleep clinical trial to improve outcomes in children with Down syndrome. 2022.

Gaser D, Peters C, Oberhoffer-Fritz R, Gotte M, Feuchtinger T, Schmid I, et al. Effects of strength exercise interventions on activities of daily living, motor performance, and physical activity in children and adolescents with leukemia or non-Hodgkin lymphoma: Results from the randomized controlled ActiveADL Study. 2022.

Sabelhaus E, Goodwin B, Bjornson K, Pham K, Walker W, Steele K. et al. Wearable technology to monitor hand movement during constraint-induced movement therapy for children with cerebral palsy. 2018.

Kobel S, Kettner S, Hermeling L, Dreyhaupt J, Steinacker JM. Objectively assessed physical activity and weight status of primary school children in Germany with and without migration backgrounds. 2019.

Pate RR, Schenkelberg MA, Dowda M, McIver KL. Group-based physical activity trajectories in children transitioning from elementary to high school. 2019.

Wang Y, He G, Ma K, Li D, Wang C. et al. Preschool children's physical activity and community environment: A cross-sectional study of two cities in China. 2022.

Matricciani L, Paquet C, Olds T. et al. Sleep profiles of Australian children aged 11-12 years and their parents: Sociodemographic characteristics and lifestyle correlates. 2020.

Manyanga T, Barnes JD, Tremblay MS. Body mass index and movement behaviors among schoolchildren from 13 countries across a continuum of human development indices: A multinational cross-sectional study. 2019.

Watson A, Dumuid D, Olds T. et al. Associations between meeting 24-hour movement guidelines and academic achievement in Australian primary school-aged children. 2022.

Migueles JH, Cadenas-Sanchez C, Ortega FB. Comparability of published cut-points for the assessment of physical activity: Implications for data harmonization. 2019.

Schwarzfischer P, Gruszfeld D, Grote V. et al. Physical activity and sedentary behavior from 6 to 11 years. 2019.

Farooq A, Basterfield L, Reilly JJ. Moderate-to-vigorous intensity physical activity and sedentary behavior across childhood and adolescence, and their combined relationship with obesity risk: A multi-trajectory analysis. 2021.

Ahn JV, Sera F, Flouri E. et al. Associations between objectively measured physical activity and later mental health outcomes in children: Findings from the UK Millennium Cohort Study. 2018.

Dumuid D, Stanford TE, Olds T. et al. Adiposity and the isotemporal substitution of physical activity, sedentary time, and sleep among school-aged children: A compositional data analysis approach. 2018.

Bringolf-Isler B, Schindler C, Probst-Hensch N. et al. Objectively measured physical activity in population-representative parent-child pairs: Parental modeling matters and is context-specific. 2018.

Longitudinal association of childhood physical activity and physical fitness with physical activity in adolescence: Insights from the IDEFICS/I.Family study. [Year Missing]

Hulst RY, et al. Accelerometer-measured physical activity, sedentary behavior, and sleep in children with cerebral palsy and their adherence to the 24-hour activity guidelines. 2023.

Jago R, Salway R, Lawlor DA. Association of BMI category with change in children's physical activity between ages 6 and 11 years: A longitudinal study. 2020.

Johnstone A, Hughes AR, Reilly JJ. An active play intervention to improve physical activity and fundamental movement skills in children of low socio-economic status: Feasibility cluster randomized controlled trial. 2019.

Kippe KO, Lagestad PA. Kindergarten: Producer or reducer of inequality regarding physical activity levels of preschool children. 2018.

Londal K, Lund S, Riiser K. et al. First graders' stationary behavior in Norwegian after-school programs: A mixed-methods investigation. 2021.

McCrorie P, Mitchell R, Ellaway A. et al. The relationship between living in urban and rural areas of Scotland and children's physical activity and sedentary levels: A country-wide cross-sectional analysis. 2020.

Migueles JH, Cadenas‐Sanchez C, Esteban‐Cornejo I. et al. Associations of sleep with gray matter volume and their implications for academic achievement, executive function, and intelligence in children with overweight/obesity. 2021

Oakley J, et al. Backyard benefits? A cross-sectional study of yard size and greenness and children’s physical activity and outdoor play. 2021.

Ranunn BM, Wichstrom L, Steinsbekk S. et al. Persistent short sleep from childhood to adolescence: Child, parent, and peer predictors. 2021.

Riiser K, Haugen ALH, Londal K. et al. Physical activity in young schoolchildren in after-school programs. 2019.

Robbins LB, Ling JY, Chang MW. Organized physical activity program participation, physical activity, and related psychosocial factors among urban adolescents. 2021.

Schmutz EA, Haile SR, Kriemler S. et al. Physical activity and sedentary behavior in preschoolers: A longitudinal assessment of trajectories and determinants. 2018.

Schwarzfischer P, Gruszfeld D, Socha P. et al. Longitudinal analysis of physical activity, sedentary behavior, and anthropometric measures from ages 6 to 11 years. 2018

Verswijveren SJJM, Lamb KE, Ridgers ND. Using compositional data analysis to explore accumulation of sedentary behavior, physical activity, and youth health. 2022.

Carver A, Cerin E, Saelens BE. Associations of home and neighborhood environments with children's physical activity in the US-based Neighborhood Impact on Kids (NIK) longitudinal cohort study. 2023.

Farooq MA, Parkinson KN, Reilly JJ. Timing of the decline in physical activity in childhood and adolescence: Gateshead Millennium Cohort Study. 2018.

Pike KC, Griffiths LJ, Dezateux C, Pearce A. et al. Physical activity among children with asthma: Cross-sectional analysis in the UK Millennium Cohort. 2019.

van Rijssen IM, Hulst RY, Gorter JW, et al. Device-based and subjective measurements of sleep in children with cerebral palsy: A comparison of sleep diary, actigraphy, and bed sensor data. 2023.

**Pooled data from articles all published prior to 2018**

Maher JP, Dzubur E, Nordgren R, Huh J, Chou C-P, Hedeker D, et al. Do fluctuations in positive affective and physical feeling states predict physical activity and sedentary time? 2017.

Tarp J, Andersen LB, Damsgaard CT, et al. Physical activity intensity, bout-duration, and cardiometabolic risk markers in children and adolescents. 2018

Hansen BH, Anderssen SA, Kolle E, et al. Cross-sectional associations of reallocating time between sedentary and active behaviours on cardiometabolic risk factors in young people: an International Children's Accelerometry Database (ICAD) analysis. 2018.

**Not intended study cohort (teachers not children or caregivers)**

Creaser AV, Frazer MT, Costa S, Bingham DD, Clemes SA. The use of wearable activity trackers in schools to promote child and adolescent physical activity: a descriptive content analysis of school staff's perspectives. 2022.

**Accelerometery data taken from a follow-up assessment (not baseline measurement)**

Guerlich K, Gruszfeld D, Grote V, et al. Sleep duration and problem behaviour in 8-year-old children in the Childhood Obesity Project. 2022.

**Qualitative only**

Goodwin BM, Sabelhaus EK, Pan YC, et al. Accelerometer measurements indicate that arm movements of children with cerebral palsy do not increase after constraint-induced movement therapy (CIMT). 2020.

Avelar AFM, Orsi KCSC, Pinheiro EM, Llaguno NS. The use of actigraphy dosimeter by school-aged children: perceptions of children and parents. 2019.

Ross AB, Quinlan A, Blanchard CM, Naylor PJ, Warburton DER, Rhodes RE. Benefits and barriers to engaging in a family physical activity intervention: a qualitative analysis of exit interviews. 2022

Muller J, Hoch AM, Zoller V, Oberhoffer R. et al. Feasibility of physical activity assessment with wearable devices in children aged 4-10 years-a pilot study. 2018.

Albrecht BM, Flaskamp FT, Koster A, Eskofier BM, Bammann K. et al. Cross-sectional survey on researchers' experience in using accelerometers in health-related studies. 2022.

Oygur I, Su ZY, Chen YA, et al. The lived experience of child-owned wearables: comparing children's and parents' perspectives on activity tracking. 2021.

Westrhenen J, et al. Parental experiences and perspectives on the value of seizure detection while caring for a child with epilepsy: a qualitative study. 2021.

Shelley J, Fairclough SJ, Knowles ZR, et al. A formative study exploring perceptions of physical activity and physical activity monitoring among children and young people with cystic fibrosis and health care professionals. 2018.

Creaser AV, Clemes SA, Costa S, et al. Applying the COM-B model to understand wearable activity tracker use in children and adolescents. 2022.

Creaser AV, Clemes SA, Costa S, et al. Exploring families' acceptance of wearable activity trackers: a mixed-methods study. 2022.
