# Supplementary material for: Adherence to Actigraphic Devices in Elementary School–Aged Children: Systematic Review and Meta-Analysis
Source: J Med Internet Res. 2025 Nov 3;27:e79718. doi: 10.2196/79718 (PMC12582557; doi:10.2196/79718)
Supplement: Multimedia Appendix 5 [file jmir-v27-e79718-s005.docx]

| **Multimedia appendix 5: Summary of extracted data from included studies** | | | | | | | | | | |
| --- | --- | --- | --- | --- | --- | --- | --- | --- | --- | --- |
| **Citation** | **Location** | **Design** | **Participant characteristics*** | **Device model** | **Actigraph purpose** | **Wear location** | **Protocol wear time** | **Analysis wear time (minimum)** | **Adherent (%)** | **Days worn** |
| Migueles, 2021 | Spain | Cross-sectional study | 110 (M = 10.02 (1.13) years, 60.42 per cent male, no diagnosis) | ActiGraph GT3X+ | Physical activity and sleep | Wrist | 7 days | 4 days | 96.36 | 6.95 (0.4) |
| Fairclough, 2019 | UK | NR | 226 (M = 9.6 (0.3) years, 42.80 per cent male, no diagnosis) | ActiGraph GT9X | Physical activity | Wrist | 7 days | 3 days | 64.60 | NR |
| Beltran-Valls, 2019 | UK | Longitudinal observational study | 617 (M = boys 7.5 (0.5)  girls 7.5 (0.4) years, 50.29 per cent male, no diagnosis) | ActiGraph GT1M | Physical activity | Waist | 7 days | 3 days | 82.17 | Boys 6.5 (0.9), girls 6.3 (1.2) |
| Pearce, 2018 | UK | Longitudinal study | 13681 (7 years, 51.50 per cent male, White: 89%, Mixed: 9.4%, Indian: 1.9%, Pakistani/Bangladeshi: 3.9%, Black or Black British: 2.7%, Other: 1.6%, no diagnosis) | ActiGraph GT1M | Physical activity | NR | 7 days | 2 days | 47.49 | NR |
| Silva, 2018 | Multinational*^1^ | Cross-sectional study | 7372 (M = 10.4 (0.6) years, 45.60 per cent male, no diagnosis) | ActiGraph GT3X+ | Physical activity | Waist | 7 days | 4 days | 83.53 | NR |
| Adank, 2021 | Netherlands | Longitudinal cohort study | 371 (M = 10.1 (0.5) years, 47.70 per cent males, no diagnosis) | ActiGraph GT3X+ | Physical activity | Waist | 7 days | 2 days | 93.50 | NR |
| Alder, 2023 | USA | Cross-sectional study | 32 (M = 5.6 (1.8) years, 75.00 per cent male, American Indian, Alaska Native (3.10%) Black, African American (18.80%) White (78.10%) Unknown (3.1%), Neurodevelopmental disorder) | ActiGraph wGT3X-BT | Physical activity and sleep | Wrist | 5 days | 4 days | 84.37 | NR |
| Allen, 2019 | Australia | NR | 345 (M = 11.30 (0.58) years, 50.00 per cent male, no diagnosis) | ActiGraph GT1M | Physical activity | Waist | 8 days | 3 days | 86.60 | NR |
| Anselma, 2023 | Netherlands | Randomised controlled trial (by school) | Int: 397 (M = 10.6 (1.0), 47.10 per cent male, no diagnosis)  Con: 303 (M = 10.6 (1.1), 49.1 per cent male, no diagnosis) | ActiGraph GT3X+ | Physical activity | Waist | 7 days | 4 days | Int: 24.94  Con: 40.02 | NR |
| Bagley, 2018 | USA | Longitudinal study | 210 (M = 11.3 (0.63) years, 54.29 per cent male, European American = 66.7% African American = 3.3%, no diagnosis) | Motionlogger Octagonal Basic | Sleep | Wrist | 7 days | 5 days | 77.90 | 5.39 (1.9) |
| Bedell, 2022 | USA | Cross-sectional study | 37 (M = 4.6 (1.2) years, 65 per cent male, 10 (27%) Hispanic  24 (65%) White,  2 (5%) Black 1 (3%) multiracial, no diagnosis) | Garmin Vívofit Jr. 2 | Physical activity | NR | 5 days | 4 days | 91.89 | NR |
| Beemer, 2020 | USA | NR | 27 (M = 8.7 (1.2) years, 43 per cent male, 38.10 % = White  33.33 % = African American  23.81 % = Asian/Pacific Islander  4.76% = Other, no diagnosis) | ActiGraph GT3X | Physical activity | Waist | 7 days | 4 days | 77.77 | 4.5 (0.7) school days 1.8 (0.4) weekend days |
| Bejarano, 2021 | USA | Cross-sectional study | 756 (M = 9.04 years, 50.00 per cent male, White, non-Hispanic = 68.0% Hispanic = 32.0%, no diagnosis) | ActiGraph GT1M | Physical activity | Waist | 7 days | 3 days | 95.80 | NR |
| Bekelman, 2021 | USA | Cross-sectional study | 778 (M = 4.6 (0.4) years, 54.00 per cent male, White 263 Hispanic 115 Black or African American 44 Other 60, no diagnosis) | ActiGraph wGT3X-BT | Physical activity | Waist | 7 days | 1 day | 76.99 | 6 (2.0) |
| Berge, 2021 | USA | Longitudinal study | 150 (M = boys 6.43 (0.81) years, girls 6.38 (0.78) years, 54.10 per cent male, Caucasian boys 19 girls 14 Black boys 13 girls 19 Hispanic boys 19 girls15 Hmong boys 18 girls 16 Native American boys16 girls 18 Somali boys 15 girls 18, no diagnosis) | ActiGraph GT1M | Physical activity | Waist | 8 days | 4 days | 98.00 | NR |
| Bolger, 2019 | Republic of Ireland | Randomised controlled trial | 228 (M = senior infant = 6.5 (0.6) years, fourth/fifth = 10.4 (0.6) years, 53.50 per cent male, no diagnosis) | ActiGraph GT3X+ | Physical activity | Waist | 7 days | 3 days | 79.82 | NR |
| Brønd, 2019 | Norway | Population study | 1145 (10 years, no diagnosis) | ActiGraph GT3X+ | Physical activity | Waist | 7 days | 1 day | 97.90 | 7 (1.5) |
| Brudy, 2020 | Germany | Cross-sectional study | Case: 162 (M = 11.8 (3.2) years, 62.96 per cent male, Cardiovascular/respiratory diagnosis)  Control (M = 10.9 (3.8) years, 48.96 per cent male, no diagnosis) | Garmin Vívofit Jr | Physical activity | Wrist | 7 days | 4 days | 100.00 | NR |
| Caserta, 2022 | Australia | Cross-sectional study | 27 (M = 6.62 (2.29) years, 63.00 per cent male, neuromotor/muscular skeletal diagnosis) | ActiGraph ActiSleep-BT | Physical activity and sleep | Wrist | 7 days | 4 days | 81.48 | NR |
| Cassim, 2021 | Australia | Population study | 682 (6 years, 50.60 per cent male, no diagnosis and cardiovascular/respiratory diagnosis) | Activinsights GENEActiv | Physical activity | Wrist | 8 days | 4 days | 57.33 | NR |
| Chen, 2020 | Singapore | Cross-sectional study (mixed methods) | 73 (M = 4.4 (1.1) years, 38.40 per cent male, Chinese = 63% with = 55.1 without = 79.2 Malay = 32.9 with = 38.8 without = 20.8 Other = 4.1 with = 6.1 without = 0%, no diagnosis) | Actigraph wGT3X-BT | Physical activity | Wrist | 7 days | 3 days | 67.12 | median = 6.7  IQR 6.1–7.0 |
| Christian, 2020 | United Kingdom | Mixed methods | 85 (9–11 years, 51.76 per cent male, no diagnosis) | Unilever Discover GENEA | Physical activity | Wrist | 7 days | 3 days | 84.71 | NR |
| Clark, 2019 | Canada | Case-control study | 272 (10-13 years, no diagnosis) | Philips Actical | Physical activity | Waist | 8 days | 1 day | 59.93 | Average 3 days |
| Costa, 2019 | Brazil | Secondary analysis | Int: 567 (M = 12.1 (1.2) years, 47 per cent male, no diagnosis)  Con: 411 (M = 12.6 (1.4) years, 44 per cent male, no diagnosis) | Actigraph GT3X + | Physical activity | Waist | 5 days | 3 days | Int: 36.33  Con: 33.58 | NR |
| Cradock, 2019 | USA | Quasi-experimental study | Int: 262 (M = 8.4 (1.8), 55 per cent male, no diagnosis)  Con: 226 (M = 8.8 (1.7), 51 per cent male, no diagnosis)  Overall sample Non-Hispanic White overall 249 (58%)  Non-Hispanic Black overall 17 (4%)  Hispanic/Latino overall 61 (14%)  Non-Hispanic Asian 28 (7%)  Non-Hispanic Asian 28 (7%)  Unknown 40 (9%) | Fitbit Charge HR | Physical activity | Wrist | 5 days | 2 days | Int: 91.98  Con: 82.30 | Int: 3.3 (0.7)  Con: 3.1 (0.8) |
| Danielsen, 2022 | Norway | Case-control study | 170 (M = 12 (3) years, 41.18 per cent male, no diagnosis) | Philips Actiwatch 2 | Physical activity and sleep | Wrist | 7 days | 4 days | 90.59 | NR |
| Draper, 2019 | South Africa | Cross-sectional study | 268 (M = 5.17 (0·70) years, 50.75 per cent male, no diagnosis) | Actigraph GT3X + | Physical activity | Waist | 7 days | 4 days | 85.45 | NR |
| Duck, 2021 | USA | Quasi-experimental study | 32 (M = 9.2 (0.41) years, 34.30 per cent male, Black 31 (88.6%) White 2 (5.7%) Other 2 (5.7%), no diagnosis) | Actigraph GT3X | Physical activity | Waist | 7 days | 2 days | 93.75 | NR |
| Evenson, 2019 | USA | Population cohort study | 1466 (8-12 years, 51.2 per cent male, Central American 112 6.0% Cuban 103 5.4% Dominican 167 12.8% Mexican 648 46.4% Puerto Rican 128 9.5% South American 68 4.0% Mixed 135 9.6% Other/missing 105 6.4%, no diagnosis) | Philips Actical version B-1; model 198–0200-03 | Physical activity | Waist | 7 days | 3 days | 75.31 | NR |
| Fang, 2020 | China | Cross-sectional study | 373 (M = 4.82 (0.45) years, 55.48 per cent male, no diagnosis) | ActiGraph GT3X + | Physical activity | Waist | 7 days | 3 days | 82.80 | NR |
| Fraysse, 2019 | Australia | Cross-sectional study | 1874 (M = 12.0 (0.4) years, 50 per cent male, no diagnosis) | Activinsights GENEActiv | Physical activity and sleep | Wrist | 8 days | 4 days | 67.29 | NR |
| Gaser, 2022 | Germany | Cross-sectional study | 41 (M = 10.0 (4.0) years, 66 per cent male, Oncology diagnosis) | Movisens Move 3 | Physical activity | Waist | 7 days | 4 days | 80.49 | 6 (1) |
| Gerber, 2021 | South Africa | Cluster randomised controlled trial | 1369 (M = 8.3 (1.4) years, 52 per cent, no diagnosis) | ActiGraph wGT3X-BT | Physical activity | Waist | 7 days | 5 days | 94.16 | 7 days = 93.8% 6 days = 5.0%) 5 days = 1.2% |
| Hall, 2019 | United Kingdom | Cross-sectional study | 92 (M = 5.37 (0.79) years, 63.16 per cent male, no diagnosis) | Activinsights GENEActiv | Physical activity | Wrist | 4 days | 4 days | 41.30 | NR |
| Holzhausen, 2020 | USA | Population study | 530 (M = 11.4 (3.3) years, 52.00 per cent male, White (self-reported) 183 78% (proxy report) 131 73% Black (self-reported) 26 11% (proxy report) 25 14% Hispanic (self-reported) 9 4% (proxy report) 13 7% Other(self-reported) 17 7% (proxy report) 10 6%, no diagnosis) | ActiGraph wGT3X-BT | Sleep | Wrist | 7 days | 2 days | 79.43 | 6.8 |
| Hulst, 2023 | Netherlands | Cross-sectional study | 60 (Median 6. 6 years, 55.60 per cent male, Neuromotor/Muscular skeletal) | ActiGraph wGT3X-BT | Physical activity and sleep | Waist | 7 days | 5 days | 91.67 | 6.7 (0.7) |
| Joensuu, 2018 | Finland | Cross-sectional study | 971 (M = 12.4 (1.3) years, 44.28 per cent male, no diagnosis) | ActiGraph GT3X + | Physical activity | Waist | 7 days | 3 days | 78.99 | NR |
| Joschtel, 2021 | Australia | Cross-sectional study | 46 (M = 7.5 (2.6) years, 63.00 per cent male, Cardiovascular/respiratory diagnosis) | ActiGraph GT3X + | Physical activity | Waist | 7 days | 3 days | 89.13 | 5.5 (1.7) |
| Kattelmann, 2019 | USA | Randomised controlled trial | Int: 108 (M = 9.4 (0.6) years, no diagnosis)  Con: 47 (M = 9.4 (0.6) years, no diagnosis) Overall sample 49 per cent male, 78.5% white | ActiGraph GT3X + | Physical activity | Waist | 7 days | 4 days | Int: 81.48  Con: 72.34 | NR |
| Kippe, 2022 | Norway | NR | 289 (4-6 years, 51.23 per cent male, 18% of the children had parents born outside Norway, no diagnosis) | Actigraph GT1M | Physical activity | Waist | 7 days | 2 days | 84.43 | NR |
| Klamm, 2022 | USA | Secondary analysis | 32 (9 years, 26.3 per cent male, black (84.2%), no diagnosis) | ActiGraph wGT3X-BT | Physical activity | Waist | 7 days | 2 days | 59.38 | NR |
| Knox, 2019 | United Kingdom | Randomised controlled trial | 49 (M = 10.63 (1.0) years, 55.1 per cent male, White Con 22 (88.0) Int 20 (83.3) All 42 (85.7) Black British Con 0 (0.0) Int 1 (4.2) All 1 (2.0) Asian Con 1 (4.0) Int 1 (4.2) All 2 (4.1) Mixed race Con 1 (4.0) Int 2 (8.3) All 3 (6.1) Other Con 1 (4.0) Int 0 (0.0) All 1 (2.0), Endocrine diagnosis) | Polar Active activity watch | Physical activity | NR | 7 days | 3 days | 87.76 | NR |
| Li, 2021 | China | NR | 86 (M = 8.45 (1.40) years, 82.56 per cent male, Neurodevelopmental disorder) | ActiGraph wGT3X-BT | Physical activity | Wrist | 7 days | 4 days | 100.00 | NR |
| Ludwig & Rauch, 2018 | Germany | NR | 108 (M = 4.86 (0.71) years, 57 per cent male, no diagnosis) | ActiGraph wGT3X-BT | Physical activity | Waist | 10 days | 4 days | 90.74 | 6.58 (0.64) |
| Manyanga, 2018 | Multinational | Cross-sectional study | 7372 (M = 10.4 (0.6) years, 45.10 per cent male, no diagnosis) | ActiGraph GT3X + | Sleep | Waist | 7 days | 4 days | 85.70 | NR |
| Mazza, 2020 | France | NR | 100 (M = 8.5 (0.3) years, 43.4 per cent male, no diagnosis) | Philips Actiwatch 2 | Sleep | Wrist | 7 days | 6 days | 89.00 | NR |
| McCrorie, 2018 | United Kingdom | Longitudinal cohort study | 1096 (10 years, 46.12 per cent male, no diagnosis) | ActiGraph GT3X + | Physical activity | NR | 8 days | 5 days | 70.62 | NR |
| Riiser, 2020 | Norway | Matched-pair cluster randomized design | 456 (5-6 years, 52.2 per cent male, no diagnosis) | ActiGraph GT3X | Physical activity | Waist | 5 days | 2 days | 93.42 | Int 4.2 (1.0)  Con 4.2 (0.9) |
| Hartman, 2020 | Multinational | Longitudinal observational study | Case: 20 (M = 10.10 (3.04) years, 100.00 per cent male, Black 0 (0%) White/Non-Hispanic 17 (90%) White/Hispanic 1 (5%) Other/Multiple 1 (5%), Neuromotor/Muscular skeletal diagnosis)  Control: 17 (M = 8.65 (3.41) years, 100 per cent male, Black 0 (0%) White/Non-Hispanic 15 (88%)  White/Hispanic 1 (6%) Other/Multiple 1 (6%), no diagnosis) | ActiGraph GT9X Link | Sleep | Wrist | 30 days | 10 days | Case: 100.00  Con: 100 | NR |
| Gråstén & Yli-Piipari, 2019 | Finland | NR | 76 (Int M = 12.23 (0.42) Con M= 12.04 (0.21), 47 per cent male, no diagnosis | ActiGraph GT3X + | Physical activity | Waist | 7 days | 3 days | 34.21 | NR |
| Gråstén, 2021 | Finland | NR | 510 (M = 11.26 (0.33), 44.12 per cent male, mostly Caucasian, no diagnosis | Actigraph GT3X+ | Physical activity | Waist | 7 days | 3 days | 88.82 | NR |
| Wiersma, 2019 | Netherlands | Population study | 1474 (M = 5.7 (0.8), 52.20 per cent male, 90.6% Dutch (n = 865), no diagnosis | ActiGraph GT3X | Physical activity | Waist | 4 days | 3 days | 64.86 | NR |
| Williams, 2022 | Hong Kong | NR | 211 (M = 12.6 (1.7), 52.17 per cent male, no diagnosis, | ActiGraph GT3X-BT | Physical activity | Wrist | 7 days | 4 days | 79.62 | 5.8 ± 0.5 |
| Verjans-Janssen, 2020 | Netherlands | Quasi-experimental study | 523 (M = 8.51 (1.06), 46 per cent male, Western = 297 (57.1%) Non-western = 223 (42.9%), no diagnosis, | ActiGraph GT3X + | Physical activity | Waist | 7 days | 2 days | 88.53 | NR |
| Chan, 2019 | Hong Kong | NR | 238 (M = 9.2 (1.6), 40.30 per cent male, no diagnosis | ActiGraph GT3X+ | Physical activity | NR | 7 days | 3 days | 80.25 | NR |
| St Laurent, 2022 | USA | Secondary analysis | 905 (M = 4.25 (0.80), 56.4 per cent male, White 183 (61.3) Black/African American 26 (8.7) Asian 13 (4.4) Native Hawaiian/Pacific Islander 2 (0.7)  Two or more racial groups 34 (11.4)  Other 21 (7.1)  Missing 19 (6.4)  Hispanic  Yes 79 (69.5) - No 207 (26.5), no diagnosis | Phillips Actiwatch Spectrum | Sleep | Wrist | 16 days | 3 days | 53.92 | 10 (3.6) |
| Wang, 2022 | China | NR | 142 (M = 13.27 (3.35), 67.74 per cent male, neurodevelopmental disorder, | ActiGraph wGT3-BT | Physical activity | Waist | 7 days | 2 days | 83.80 | NR |
| Yu, 2021 | Hong Kong | Cross-sectional study | Case: 88 (8 years 5 months, 67.12 per cent male, neurodevelopmental disorder)  Con: 100 (8 years 5 months, 48.49 per cent male, no diagnosis) | ActiGraph GT3X + | Physical activity | Waist | 7 days | 3 days | Case: 98.86  Con: 85.00 | NR |
| ten Velde, 2021 | Netherlands | Randomised controlled trial | 710 (M = 10 (1), 45 per cent male, no diagnosis) | ActiGraph GT3X | Physical activity | Waist | 7 days | 4 days | 70.85 | NR |
| Aguilar-Farias, 2020 | Chile | Cross-sectional study | 249 (M = 10.0 (0.82), 47.30 per cent male, no diagnosis) | ActiGraph GT3X + | Physical activity | Waist | 7 days | 4 days | 85.54 | NR |
| Clevenger, 2022 | United Kingdom | Secondary analysis | 488 (M = 13.0 (1.1), 53.28 per cent male, no diagnosis) | ActiGraph wGT3X-BT | Physical activity | Waist | 7 days | 1 day | 91.19 | 5.3 (2.0) |
| Kjellberg Olofsson, 2023 | Sweden | NR | 48 (M = 12.72 (3.48), 77.4 per cent male, Cardiovascular/respiratory) | Axivity AX3 | Physical activity | Waist | 7 days | 4 days | 85.42 | 6.9 (0.3) |
| Callaghan, 2021 | United Kingdom | Randomised controlled trial | Case: 82 (Cardiovascular/respiratory)  Con: 81 (no diagnosis)  Overall sample: (M = 8.4 (1.5) 61.30 per cent male | ActiGraph GT3X-BT | Physical activity | Waist | 7 days | 4 days | 95.71 | 6.53 (0.95) |
| Oakley, 2021 | Australia | Cross-sectional study | 682 (M = 6.2 (0.4), 50.6 per cent male, no diagnosis) | Activinsights GENEActiv | Physical activity | Wrist | 8 days | 4 days | 57.33 | NR |
| Palmer, 2018 | USA | NR | Case: 75 (M = 8.98 (1.56), Caucasian 54.7% (41) African American 4% (3), Asian 1.3% (1), Hispanic 13.3% (10), Other/Biracial 26.7% (20), other psychiatric disorder)  Con: 38 (M = 8.98 (1.56), Caucasian 60.5% (23) African American 5.3% (2), Asian 2.6% (1), Hispanic 18.4% (7), Other/Biracial 13.2% (5)  Overall sample: 48.7 per cent male, Caucasian 56.6% (64) African American 4.4% (5), Asian 1.8% (2), Hispanic 15% (17), Other/Biracial 22.1% (25), no diagnosis) | Ambulatory Monitoring Inc Micro Motionlogger Sleep Watches | Physical activity and sleep | Wrist | 7 days | 5 days | 96.46 | NR |
| Dahlgren, 2021 | Sweden | Cross-sectional study | 121 (M = 12.1 (1.5), 55.37 per cent male, no diagnosis) | SCRIIN Activity tracker | Physical activity | Waist | 7 days | 3 days | 63.64 | NR |
| Skjåkødegård, 2020 | Norway | Cross-sectional study | 170 (M = 12.4, 42.28 per cent male, no diagnosis) | Philips Actiwatch 2 | Physical activity and sleep | Wrist | 7 days | 4 days | 90.58 | 154 provided valid recordings for seven consecutive days and, 14 for 6 or 5 days |
| Harrex, 2018 | New Zealand | cross-sectional study | 465 (10.2 (0.6), 49 per cent male, no diagnosis) | ActiGraph GT3X+ | Physical activity and sleep | Wrist | 8 days | 3 days | 98.10 | Almost all (98%) participants included in these analyses provided 5 or more valid days. |
| Park, 2018 | Republic of Korea | NR | 49 (9−12 years, 100 per cent male, no diagnosis) | Omron Active Style Pro | Physical activity | Waist | 7 days | 4 days | 82.22 | NR |
| Van Kann, 2019 | Netherlands | NR | 127 (M = 9.18, 50.43 per cent male, no diagnosis) | ActiGraph GT3X + | Physical activity | Waist | 7 days | 3 days | 92.13 | 3.96 |
| Chen, 2023 | China | Cross-sectional study | 596 (M = 11.89 (2.09), 49.87 per cent male, no diagnosis) | ActiGraph wGT3X-BT | Physical activity and sleep | Waist | 7 days | 3 days | 68.46 | NR |
| Chong, 2021 | Australia | Longitudinal study | 135 (M = 11.7 (0.5), 43.7 per cent male, no diagnosis) | Activinsights GENEActiv | Physical activity and sleep | Wrist | 6 days | 3 days | 94.07 | NR |
| Talarico & Janssen, 2018 | Canada | Compositional data analysis | 458 (M = 11.7, 49.8 per cent male, White 88.2 per cent Non-white 11.8 per cent, no diagnosis) | Philips Actical | Physical activity and sleep | Waist | 7 days | 4 days | 94.80 | NR |
| Herbert, 2022 | Poland | NR | 565 (M = 5.4 (0.6), 48.09 per cent male, no diagnosis) | ActiGraph GT3X-BT | Physical activity | Waist | 7 days | 4 days | 96.81 | NR |
| Salin, 2019 | Finland | NR | 592 (M = 11.3 (0.3), 47.80 per cent male, no diagnosis) | Actigraph wGT3X-BT | Physical activity | Waist | 7 days | 3 days | 76.52 | 5.24 |
| Li, 2021 | USA | Longitudinal study | 103 (M = 6.46 (0.62), 50.60 per cent male, 51 Hispanic (43 were Hispanic White, six were Hispanic Black, and two were Hispanic mixed race) and 38 (non-Hispanic) White children, no diagnosis) | Philips Actiwatch 2 | Sleep | Wrist | 5 days | 5 days | 88.80 | NR |
| Lai, 2020 | China | Cross-sectional study | 637 (M = 9.17 (1.60), 51.87 per cent male, no diagnosis) | ActiGraph GT3X | Physical activity | Waist | 7 days | 4 days | 61.70 | NR |
| Willoughby, 2018 | USA | Cross-sectional study | 106 (M = 4.4 (0.7), 46.00 per cent male, Caucasian or non-Hispanic white = 46.4% African American or non-Hispanic black = 27.4% Hispanic = 8.3 Asian = 3.6% More than one race/ethnicity = 14.3%, no diagnosis) | Actigraph wGT3X-BT | Physical activity | Waist | 5 days | 3 days | 83.02 | 4.1 (0.8) |
| Wyszyńska, 2021 | Poland | Cross-sectional study | 707 (M = 5.55, 48.97 per cent male, no diagnosis) | Actigraph GT3X-BT | Physical activity and sleep | Waist | 7 days | 4 days | 99.15 | 6.7 |
| Xu, 2022 | China | Survey study | 134 (M = 12.70 (2.84), 64.65 per cent male, neurodevelopmental disorder) | ActiGraph GT3X | Physical activity and sleep | Wrist | 7 days | 3 days | 87.31 | NR |
| Yang, 2019 | China | NR | 120 (M = 13.1 (2.5), 50.00 per cent male, no diagnosis) | ActiGraph wGT3X-BT | Physical activity | Waist | 7 days | 5 days | 88.33 | NR |
| Yoong, 2019 | Australia | Randomised controlled trial | Int: 38 (M = 4.3 (0.5), 63.00 per cent male, no diagnosis)  Con: 38 (M = 4.5 (0.6), 53.00 per cent male, no diagnosis) | Actigraph GT3X+ | Physical activity | Waist | 7 days | 3 days | Int: 50.00  Con: 52.63 | NR |
| Zhang, 2020 | China | Within-subjects design | 81 (M = 5.25 (0.52), 48.78 per cent male, no diagnosis) | Actigraph GT3X | Physical activity | Waist | 9 days | 4 days | 77.78 | NR |
| So, 2021 | USA | NR | 55 (M = 9.11 (1.36), 42.27 per cent male, Caucasian (n=33) African American (n=17) biracial/other (n=3) Asian American (n=2) Children primarily identified as Non-Hispanic/Latino (n=38), no diagnosis) | Ambulatory Monitoring Inc Micro Motionlogger Sleep Watches | Sleep | Wrist | 5 days | 5 days | 89.00 | NR |
| Sprengeler, 2020 | Germany | Case-crossover study | 54 (M = 8.4 (0.7), 38.50 per cent male, no diagnosis) | PAL Technologie activPAL inclinometer | Physical activity | Leg | 10 days | 2 days | 96.30 | NR |
| Abdollahi, 2024 | Finland | Cross-sectional study | 864 (M= 4.76 (0.89), 51.00 per cent male, no diagnosis) | ActiGraph wGT3X-BT | Sleep | Waist | 7 days | 4 days | 88.67 | NR |
| Downing, 2021 | Australia | Longitudinal cohort study | 766 (M = 4.6 (0.7), 54.00 per cent male, no diagnosis) | ActiGraph GT1M | Physical activity | Waist | 8 days | 4 days | 98.96 | NR |
| Mücke, 2021 | Germany and Switzerland | Cross-sectional study | 91 (M = 11.16 (1.25), 57.14 per cent male, no diagnosis) | ActiGraph | Physical activity | Wrist | 7 days | 4 days | 87.91 | NR |
| Beunders, 2023 | Netherlands | Cross-sectional study | 952 (10 years old, 47.32 per cent male, Dutch 745 (83.4) non-Dutch 148 (16.6), no diagnosis) | Activinsights GENEActiv | Physical activity and sleep | Wrist | 9 days | 7 days | 93.91 | NR |
| McGarty, 2021 | United Kingdom | Pilot Study | 21 (M = 9.57 (1.21), 76.20 per cent male, neurodevelopmental disorder | ActiGraph wGT3X+ | Physical activity | Waist | 5 days | 4 days | 90.48 | NR |
| McMullen, 2019 | Canada | Cross-sectional study | 56 (Cardiac M = 10.2 (4.9) (3–17) Respiratory M = 9.5 (3.4) (4–16) Rheumatology M = 10.5 (3.7) (5–17)  Overall sample: 42.86 per cent male | Philips Actical 2.1 | Physical activity | Waist | 7 days | 3 days | 83.93 | 6.4 (1.2) |
| Mughal, 2020 | United Kingdom | Exploratory Study | 95 (All groups 6-12 years Autism 8.42 (1.81) FASD 9.60 (2.48) no diagnosis 8.12 (1.29)  Overall sample: 58.95 per cent male | CamNTech Actiwatch 8 | Sleep | Wrist | 7 days | 4 days | 89.47 | NR |
| Nakabazzi, 2020 | Uganda | Cross-sectional study | 309 (M = 10.95, 44.14 per cent male, no diagnosis) | ActiGraph GT3X+ | Physical activity | Waist | 7 days | 4 days | 89.00 | 6.3 (1.1) |
| Nathan, 2020 | Australia | Cluster-randomized controlled trial | 2148 (Int M = 7.96 (2.03)  (Con M = 8.05 (2.05)  Overall sample: 49.82 per cent male, no diagnosis) | ActiGraph GT3X+ | Physical activity | Wrist | 5 days | 3 days | 86.69 | NR |
| Patton, 2022 | USA | Pilot Study | 25 (M = 4.2 (1.7), 44.00 per cent male, Non-Hispanic White 24 (96%), endocrine diagnosis) | Philips ActiWatch 2 | Physical activity | Wrist | 7 days | 4 days | 88.00 | 6.4 (1.3) |
| Crotti, 2021 | United Kingdom | Cluster Randomized Controlled Trial | 344 (5-6 years, 45.00 per cent male, White British (%): LP: 68% NLP: 52% Cont: 50%,  Overall sample: no diagnosis | ActiGraph GT9X | Physical activity | Wrist | 7 days | 4 days | 76.16 | NR |
| Henriques-Neto, 2021 | Portugal | Cross-sectional study | 2696 (M = 13.4 (2.8), 46.67 per cent male, no diagnosis) | ActiGraph GT1M | Physical activity | Waist | 7 days | 3 days | 90.36 | NR |
| Kobel, 2020 | Germany | Cluster-randomised study | 231 (M = 7.0 (0.6), 45.89 per cent male, no diagnosis) | CamNtech Actiheart | Physical activity | Chest | 6 days | 3 days | 66.67 | NR |
| Yu, 2019 | Taiwan | Cross-sectional Study | 90 (M = 9.92 (1.56), 94.52 per cent male, neurodevelopmental disorder) | Actigraph wGT3X+ | Physical activity | Waist | 7 days | 4 days | 83.33 | NR |
| Joschtel, 2019 | Australia | Cross-sectional Study | 46 (M = 7.5 (2.6), 63.04 per cent male, Cardiovascular/respiratory diagnosis | ActiGraph GT3X+ | Physical activity | Waist | 7 days | 4 days | 78.26 | 5.9 (1.3) |
| Kwon, 2022 | USA | Cross-sectional Study | 352 (3-5 years old, 50.17 per cent male, no diagnosis) | ActiGraph GT3X+ | Physical activity | Wrist | 9 days | 1 day | 85.51 | NR |
| Manyanga, 2019 | Mozambique | Cross-sectional study | 683 (M = 10.1 (0.8), 47.10 per cent male, no diagnosis) | Actigraph GT3X+ | Physical activity and sleep | Waist | 7 days | 4 days | 76.65 | NR |
| Cremone, 2018 | USA | NR | 185 (M = 4.37 (0.79), 48.11 per cent male, 51.3% of children were White/Caucasian, 11.1% Black/African American, 1.7% Native American/Alaskan Native, 0.9% Asian Indian, 1.7% Chinese, 2.6% Vietnamese, 0.9% Other, Pacific Islander, 16.2% multiracial, and 2.6% identified as “other.”, no diagnosis) | Philips Respironics Spectrum 2 | Sleep | Wrist | 16 days | 3 days | 68.65 | 7.23 (1.75) |
| Higgins, 2021 | New Zealand | Cross-sectional study | 503 (9-11.5 years, 50.39 per cent male, no diagnosis) | ActiGraph GT3X+ | Sleep | Wrist | 8 days | 3 days | 90.54 | NR |
| Schroeder, 2020 | USA | Cross-sectional study | 114 (M = 9.4 (0.9), 49.12 per cent male, no diagnosis) | Actigraph GT3X+ | Physical activity | Waist | 7 days | 2 days | 95.61 | NR |
| Tsuda, 2020 | USA | NR | 72 (M = 4.38 (0.85), 54.17 per cent male, Caucasian n= 35, African American n= 28, Asian n= 5, Other n= 4, no diagnosis) | ActiGraph wGT3X-BT | Physical activity | Waist | 3 days | 3 days | 87.50 | NR |
| Wang, 2022 | China | Cross-sectional study | 471 (M = 5.07 (0.94), 50.66 per cent male, no diagnosis) | Actigraph GT3X+ | Physical activity | Waist | 7 days | 3 days | 64.55 | NR |
| Zask, 2023 | Australia | NR | Case: 101 (M = 9.8 (0.10)  Con: 89 (M = 9.6 (0.10)  Overall sample: 56.18per cent male, no diagnosis) | ActiGraph wGT3X-BT | Physical activity | Wrist | 7 days | 3 days | Case: 79.21  Con: 67.42 | NR |
| Wright, 2020 | NR | Cross-sectional study | 120 (M = 8.8 (1.9), 65.00 per cent male, no diagnosis) | ActiGraph wGT3X-BT | Physical activity | Waist | 7 days | 4 days | 95.00 | NR |
| Brazendale, 2018 | USA | Within-subjects study | 52 (M = 8.2, 43.00 per cent male, 100% African American, no diagnosis) | ActiGraph GT9X+ Link | Physical activity and sleep | Wrist | 9 days | 5 days | 57.69 | 8.5 |
| Riso, 2018 | Estonia | Cross-sectional study | 353 (M = 10.96 (0.72), 45.50 per cent male, no diagnosis) | ActiGraph GT3X | Physical activity | Waist | 7 days | 3 days | 40.23 | NR |
| Yamakita, 2019 | Japan | Cross-sectional study | 174 (M = 10.8 (0.4), 47.13 per cent male, Neuromotor/Muscular skeletal diagnosis) | Suzuken Lifecorder GS | Physical activity | Waist | 14 days | 4 days | 84.48 | NR |
| Leppänen, 2022 | Finland | Cross-sectional study | 864 (M = 4.7 (0.9), 52.73 per cent male, no diagnosis) | ActiGraph wGT3X-BT | Physical activity | Waist | 7 days | 4 days | 86.92 | 6.7 (0.57) |
| Tan, 2022 | Singapore | NR | 228 (10 years: 20 (41) 11 years: 28 (57) 12 years: 1 (2), 59.18 per cent male, no diagnosis) | Actigraph wGT3X-BT | Physical activity and sleep | Wrist | 7 days | 3 days | 68.92 | NR |
| McLellan, 2020 | United Kingdom | NR | 280 (M = 9.8 (1.1), 50.38 per cent male, no diagnosis) | ActiGraph GT3X+ | Physical activity | Wrist | 7 days | 3 days | 50.07 | NR |
| Winsor, 2023 | NR | Cross-sectional study | 43 (M = 9.47 (2.62), 61.11 per cent male, Neuromotor/Muscular skeletal diagnosis) | Philips Actiwatch 2 | Sleep | Wrist | 14 days | 7 days | 83.72 | NR |
| Lott, 2021 | USA | Cross-sectional study | Int: 70 (M = 8.7 (2.0), Neuromotor/Muscular skeletal diagnosis)  Cont: 10 (M = 9.2 (1.3), no diagnosis)  Overall sample: 100.00 per cent male | ActiGraph GT3X | Physical activity | Waist | 7 days | Int: 7 days  Con: 5 days | Int: 71.42  Con: 100.00 | NR |
| Kariippanon, 2022 | Multinational | Cross-sectional study | 1207 (M = 4.5 (0.5), 51.12 per cent male, no diagnosis) | PAL Technologies, activPAL inclinometer | Physical activity | Leg | 3 days | 1 day | 88.73 | 2.4 |
| Lu, 2022 | China | Cross-sectional study | 169 (M = 5.4 (0.9), 56.70 per cent male, no diagnosis) | ActiGraph GT3X | Physical activity | Waist | 7 days | 3 days | 79.29 | NR |
| Vyhlídal, 2022 | Czech Republic | Cross-sectional study | 28 (Median age 11.7, 46.15 per cent male, oncology diagnosis) | Actigraph wGT3X+ | Physical activity | Wrist | 20 days | 4 days | 92.86 | NR |
| da Costa, 2022 | Canada | NR | 192 (M = 9.0 (1.7), 47.80 per cent male, no diagnosis) | Philips Actical | Physical activity | Waist | 5 days | 4 days | 82.81 | NR |
| Robbins, 2020 | USA | Quasi-experimental design | Int: 39 (M = 11.3 (0.8), 68.40 per cent male, White = 18.4 Black = 60.5 Mixed racial or other = 21.1, no diagnosis)  Con: 45 (M = 11.9 (0.8), 32.60 per cent male, White = 25.6 Black = 53.5 Mixed racial or other =20.9, no diagnosis) | ActiGraph GT3X+ | Physical activity | Waist | 7 days | 3 days | Int: 48.72  Con: 68.89 | NR |
| Schwarzfischer, 2018 | Multinational | Randomised control trial | 661 (M = 6.1 (0.1), 44.10 per cent male, no diagnosis) | SenseWear Armband 2 | Physical activity | Upper arm | 3 days | 2 days | 63.09 | NR |
| Lambrechtse, 2021 | Switzerland | Feasibility study | 24 (Median 6 (IQR 5.00, 6.00), 50.00 per cent male, no diagnosis) | Withings® Go activity tracker | Physical activity | Wrist | 10 days | 10 days | 91.70 | NR |
| Sherry, 2019 | United Kingdom | Cross-sectional study | 137 (M = 9.8 (0.3), 50.63 per cent male, British south Asian 55  White British 18  Mixed ethnicity 6, no diagnosis) | PAL Technologie activPAL inclinometer | Physical activity | Leg | 7 days | 4 days | 57.66 | School day = 5.2 (1.2)  weekend = 1.9 (0.3) |
| Dumuid, 2021 | Australia | Cross-sectional study | 1874 (M = 12.0 (0.4), 51.00 per cent male, no diagnosis) | Activinsights GENEActiv | Physical activity | Wrist | 8 days | 4 days | 68.25 | NR |
| Abel, 2018 | USA | NR | 42 (M = 5.43 (2.26), 85.00 per cent male, Caucasian (88%), neurodevelopmental disorder) | Ambulatory Monitoring Inc, Micro Motionlogger Sleep Watches | Sleep | NR | 5 days | 3 days | 100.00 | NR |
| Kidokoro, 2019 | Japan | Quasi-experimental study | Int: 22 (50 per cent male)  Con: 21 (65 per cent male)  Overall sample: (M = 11.3 (0.5), no diagnosis) | ActiGraph wGT3X-BT | Physical activity | Waist | 5 days | 4 days | Int: 81.82  Con: 95.24 | NR |
| Kallio, 2020 | Finland | Longitudinal study | 970 (M = 12.5 (1.3), 47.63 per cent male, no diagnosis) | ActiGraph GT3X+ wGT3X+ | Physical activity | Waist | 7 days | 3 days | 79.48 | NR |
| Ranum, 2019 | Norway | Population study | 801 (M = 6.0 (0.2), 50.70 per cent male, 771 [96.5%] Norwegian, no diagnosis) | ActiGraph GT3X | Sleep | Waist | 7 days | NR | 85.77 | NR |
| Swartz, 2019 | USA | Cluster-randomised controlled trial | 99 (M = 10.2 (1.4), 56.57 per cent male, White: 69 Black/African American: 3 Asian: 8 Mixed Race: 7 Hispanic: 8, no diagnosis) | Actigraph GT3X+ wGT3X-BT | Physical activity | Waist | 5 days | 1 day | 100 | NR |
| Santiago-Rodríguez, 2022 | USA | Cross-sectional study | 56 (M = 9.2 (1.91), 52.17 per cent male, no diagnosis and neurodevelopmental disorder) | Actigraph GT3X+ GT1M | Physical activity | Waist | 7 days | 3 days | 41.07 | NR |
| Sánchez-Oliva, 2020, 2020 | Spain | Longitudinal study | 1120 (M = 11.72 (2.39), 50.50 per cent male, no diagnosis) | ActiGraph GT1M, GT3X GT3X+ | Physical activity | Waist | 7 days | 3 days | 98.13 | NR |
| Salmon, 2023 | Australia | Cluster randomised controlled trial | 481 (M = 8.4 (0.7), 43 per cent male, no diagnosis) | ActiGraph GT3X | Physical activity | Waist | NR | 3 days | 71.10 | NR |
| Gerber, 2019 | Switzerland | Cross-sectional study | Case: 15 (M = 13.7 (3.4), 40 per cent male, Neuromotor/Muscular skeletal diagnosis) Con: 14 (M = 13.6 (3.0), 42.86 per cent male, no diagnosis) | Gait Up, Renens, Switzerland  Physilog4 | Physical activity | Worn 1 on each thigh and shank and 1 on the trunk | NR | 3 days | Case: 73.33 con 85.71 | NR |
| Mitchell, 2018 | United Kingdom | Randomised controlled trial | 20 (M = 11.6 (2.5), 43.75 per cent male, endocrine diagnosis) | Actigraph GT3X+ | Physical activity | Waist | 7 days | 3 days | 80.00 | NR |
| Bloemen, 2019 | Netherlands | Observational study | 34 (M = 13.7 (3.2), 58.82 per cent male, Neuromotor/Muscular skeletal diagnosis) | 2M Engineering, Veldhoven, the Netherlands, VitaMove | Physical activity | For non-ambulatory participants: one recorder is placed on the sternum and a recorder is placed on each wrist. Participants who were both walking, and wheelchair-using wore two additional recorders, one on each thigh. | 3 days | 1 day | 97.06 | NR |
| Loram, 2024 | Australia | Cross-sectional study | 454 (M = 13.21 (2.37), 65.15 per cent male, no diagnosis, neurodevelopmental other psychiatric disorder) | ActiGraph wGT3X-BT | Sleep | Wrist | NR | 3 days | 82.16 | NR |
| Price, 2018 | United Kingdom | Randomised Controlled Trial | 886 (M = 9.7 (0.3), 47.74 per cent male, no diagnosis) | ActivInsights GENEActiv | Physical activity | Wrist | NR | 4 days | 97.50 | NR |
| Pate, 2019 | USA | Prospective cohort study | 1080 (M = 10.6 (0.5), 46.39 per cent male, White: 317, 38.3% African American: 291, 35.1% Hispanic: 79, 9.5% Other: 141, 17.0%, no diagnosis) | ActiGraph GT1M GT3X | Physical activity | NR | 7 days | 4 days | 91.85 | NR |
| Parry, 2019 | Australia | Repeated-measures crossover design study | 23 (100 per cent male, no diagnosis) | Actigraph GT9X Link | Physical activity | One attached to the thigh, and one attached to the waist | Thigh = start to the end of the school day  Waist = 7 days | 6 hours a day during school hours | 86.96 | NR |
| Nigg, 2021 | Germany | Cross-sectional study | 2734 (M = 12.52 (3.30), 47.05 per cent male, no diagnosis | ActiGraph GT3x + wGT3X-BT | Physical activity | Waist | 7 days | 5 days | 83 | NR |
| Naya, 2021 | USA | Cross-sectional study | 161 (M = 9.60 (0.88), 55.63 per cent male, White/Caucasian 22 (15.6%) Black/African American 17 (12.1%) Asian/Native Hawaiian/Other Pacific Islander 13 (9.2%) Hispanic 83 (58.9%) Other 6 (4.3%), no diagnosis | ActiGraph GT3X+ | Physical activity | Waist | 7 days | NR | 98.13 | NR |
| Mora-González, 2019 | Spain | Cross-sectional study | 100 (M = 10.1 (1.1), 58.00 per cent male, no diagnosis | Actigraph GT3X+ | Physical activity | Wrist and Waist simultaneously | 7 days | NR | 96 | NR |
| Molina-García, 2021 | Spain | Cross-sectional study | 83 (M = 8.78 (1.69), 48.19 per cent male, no diagnosis) | Actigraph GT3X+ | Physical activity | NR | NR | 4 days | 98.80 | NR |
| Miadich, 2019 | USA | Longitudinal Study | 381 (M = 8.45 (0.45), 50.40 per cent male, Black/African American 4.2% Hispanic/Latino 25.2% Non-Hispanic White/European American 56.4% Asian/Asian American 5.2% Native American 1.0% Native Hawaiian or Pacific Islander 1.6% Multiracial/ethnic or unknown 6.3%, no diagnosis) | Ambulatory Monitoring Motion Logger Micro Watch | Sleep | Wrist | 7 days | NR | 91.30 | 6.82 (0.66) |
| Jakubec, 2020 | Czech Republic | Cross-sectional study | 862 (M = 13.9 (2.8), 43 per cent male, no diagnosis) | Actigraph wGT3X-BT (children) and GT9X Link (adolescents) | Physical activity and sleep | Wrist | 7 days | 4 days | 95.92 | NR |
| Sturm, 2021 | Germany | Cluster randomised control trial | 484 (M = 11.61 (0.55) 0.00, per cent male, no diagnosis) | ActiGraph GT3X, GT9X, wGT3X-BT | Physical activity | NR | 7 days | 4 days | 77.27 | NR |
| Sinisterra, 2020 | NR | Pilot study | 13 (M = 4.7 (0.9), 63.10 per cent male, White non-Hispanic 31 67.3%, endocrine diagnosis) | ActiGraph wGT3X-BT | Sleep | NR | NR | 4 days | 84.62 | NR |
| Silva, 2018 | Portugal | Cluster non-randomised controlled trial | 49 (Int M = 11.8 (0.4), 54.5 per cent male Con M = 11.6 (0.5), 40.7 per cent male, Caucasian Int = 21 (95.5%)  Con = 26 (96.3%), no diagnosis) | PAL Technologies Limited, Glasgow, UK  ActivPAL™ micro inclinometer  Actigraph GT3X+ | Physical activity | ActivPAL = Leg Actigraph = Waist | ActiPAL = 7 days Actigraph 7 days | 4 days | 100 | NR |
| Pirnes, 2022 | Finland | NR | 970 (M = 12.5 (1.3), 47.50 per cent male, no diagnosis) | ActiGraph triaxial GT3X+and wGT3X+ | Physical activity | Waist | 7 days | 3 days | 79.07 | NR |
| Bachner, 2020 | Germany | NR | 545 (100 per cent male, no diagnosis) | ActiGraph models GT3X (n= 46) to wGT3X-BT (rest) | Physical activity | Waist | 7 days | 4 days | 77.98 | NR |
| Nyberg, 2020 | Sweden | Cross-sectional study | 3302 (M = 14.4 (2.6), 47 per cent male, no diagnosis) | ActiGraph GT3X GT3X+ | Physical activity | Waist | 7 days | 3 days | 73.26 | NR |
| Franceschi, 2022 | Italy | Prospective study | 47 (M = 12.65 (3.14), 46.80 per cent male, 83% (39/47) of the patients were of Caucasian origin, endocrine diagnosis) | Fitbit Alta Hr | Physical activity and sleep | Waist | 28 days | 6 days | 100 | FSL1 5.98±1.17 nights  FSL2 6.11±1.00 nights |
| Cabanas-Sánchez, 2018 | Spain | Cross-sectional study | 1578 (M = 12.1 (2.5), 49.87 per cent male, no diagnosis) | Actigraph GT1M, GT3X GT3X+ | Physical activity | Waist | 7 days | 3 days | 75.85 | NR |
| Armstrong, 2021 | USA | Longitudinal study | 240 (M = 7.00 (1.2), 46.10 per cent male, African American 64.6  White 28.9 Other 6.6, no diagnosis) | Fitbit Charge 2 | Physical activity and sleep | Wrist | 140 days | NR | 81.66 | NR |
| Bringolf-Isler, 2018 | Switzerland | Cross-sectional study | 2032 (6 – 16 years, 51.37 per cent male, no diagnosis) | ActiGraph GT1M GT3X | Physical activity | Waist | 7 days | 3 days | 66.88 | NR |
| Sprengeler, 2021 | Belgium, Cyprus, Estonia, Germany, Hungary, Italy, Spain, and Sweden | Prospective cohort study | 16229 (Boys M = 6.1 (1.8) Girls M = 6.3 (1.7), no diagnosis) | Actigraph GT1M, GT3X ActiTrainer | Physical activity | Waist | 3 days | 3 days | 51.94 | NR |
| De Meester, 2018 | USA | Cross-sectional study | 361 (M = 9.49 (1.24), 49.86 per cent male, non-Hispanic White (47.9%), Hispanic (25.8%) and African American (22.1%), no diagnosis) | ActiGraph GT3X+ | Physical activity | Waist | 5 days | NR | 90.34 | NR |
| Clemes, 2020 | United Kingdom | Pilot cluster RCT | Int = 86 (M = 9.3 (0.4), 55.8 per cent male  Con = 90 (M = 9.3 (0.5), 55.6 per cent male,  White British control 18 (20.0%) inter 45 (52.3%) South Asian control 59 (65.6%)inter 26 (30.2%) Other control 13 (14.4%) inter 15 (17.4%), no diagnosis) | PAL Technologies activPAL3 micro accelerometer  ActiGraph GT3X+ | Physical activity | activPAL3 = leg ActiGraph GT3X = waist simultaneously | activPAL3 = NR ActiGraph GT3X = 7 days | 1 day baseline and follow up | activPAL = 80.1 Actigraph = 94.3 | ActiPal (weekdays only)  Int = 3.5 (0.9) (n = 52)  Con = 3.7 (1.3) (n = 57)  ActiGraph (weekdays only)  Int 3.6 (1.3) (n=72)  Con = 3.8 (1.4) (n= 74) |
| Innerd, 2019 | United Kingdom | Non-randomised exploratory, controlled before-and-after design with a mixed methods approach. | 152 (Int M = 9.9 (0.7) Con M = 10.1 (0.7), 50 per cent male, no diagnosis) | ActiGraph GT3X | Physical activity | Waist | 7 days | 4 days | Int = 64 Con = 61 |  |
| Shoesmith, 2020 | Australia | Cross-sectional study | 3299 (M = 8.11 (0.71), 50.04 per cent male, no diagnosis) | ActiGraph GT3X + GT9X | Physical activity | Wrist | 5 days | 3 days | 86.60 |  |
| Buchan & Maylor, 2023 | United Kingdom | NR | 146 (M = 10.4 (0.6), 50 per cent male, no diagnosis) | Actigraph wGT3×+ PAL Technologies activPAL | Physical activity | Wrist and leg | 7 days | 1 day | Actigraph = 80.14  actiPAL 82.87 | 3.1 (1.5) |
| Strugnell, 2023 | Australia | Cluster randomised trial | 1826 (M = 10.7 (1.1), 52.80 per cent male, no diagnosis) | ActiGraph wGT3X-BT | Physical activity | Waist | 7 days | 3 days | 77.00 |  |
| Seljebotn, 2019 | Norway | Cluster-randomised controlled trial | Int: 228, 51.98 per cent male Con: 219, 49.32 per cent male  Overall:  9-10 years old, no diagnosis) | ActiGraph GT1M GT3X GT3X+ | Physical activity | Waist | 7 days | 2 days | Int: 92.94 Con: 93.97 |  |
| Sacheck, 2021 | USA | Randomised controlled trial | Int 1: 369 (M = 8.7 (0.7), 41.2 per cent male Int 2: 311(M = 8.7 (0.7), 44.7 per cent male Con: 299 (M = 8.7 (0.6), 46.8 per cent male, no diagnosis) | ActiGraph GT3X+ wGT3X-BT | Physical activity | Waist | 7 days | 3 days | 55.90 | Int 1: 4.5 (0.8) Int 2: 4.5 (0.8) Con: 4.7 (0.9) |
| Trickett, 2019 | NR | Case-Controlled Study | 22 (M = 9.43 (3.72), 40 per cent male, neurodevelopmental disorder and no diagnosis) | Philips Actiwatch 2 | Sleep | NDD: 15 leg and 5 wrist.  TD: all wrist. | 7 days | 4 days | 90.91 | NR |
| Reedman, 2019 | Australia | Randomised waitlist-controlled trial | 37 (Int M = 9.8 (1.5), 44.4 per cent male)  Waitlist M = 10. 2 (1.3), 52.6%per cent male)  Overall sample: Neuromotor/Muscular skeletal | ActiGraph GT3X | Physical activity | Waist | 7 days | 1 day | Int: 83.3 Waitlist: 73.7 | Baseline Int = 5.87 (2.29) Waitlist = 5.29 (1.77) |
| Duncan, 2023 | United Kingdom | NR | 146 (M = 10.4 (0.6), 50 per cent male, no diagnosis) | ActiGraph wGT3×+ activPAL Micro4 | Physical activity | Wrist | 7 days | 1 day | wGT3×: 80.14 activPAL: 82.88 | 3.1 (1.5) |
| Braaksma, 2022 | Netherlands | Mixed methods study | 20 (M = 10.0 (1.6), 80 per cent male, neurodevelopmental disorder) | ActiGraph wGT3x-BT Fitbit zip | Physical activity | NR | wGT3x-BT: 7 days Fitbit – NR | NR | wGT3x-BT = 37.5 Fitbit zip = NR | NR |
| Philbrook, 2022 | USA | Cross-sectional study | 51 (M = 4.47 (0.89), 53 per cent male,  White 80% 18% as biracial 2% as Black, no diagnosis) | ActiGraph wGT3X-BT actiwatch | Sleep | Wrist | 7 days | NR | 96.08 | 6.3 (1.3) |
| Thorpe, 2021 | United Kingdom | Feasibility Study | 46 (Median = 10.0 (6.2–15.1), 100 per cent male, haemophilia type A or B diagnosis) | ActiGraph GT3X | Physical activity | Waist | 7 days | NR | 89.13 | NR |
| Traube, 2020 | NR | Cross-sectional study | 56 (Age 0–2: 4 Age >2–5: 9 Age >5–13:26 Age >13–18: 17, 53.57 per cent male, oncology diagnosis) | Ambulatory Monitorin Micro Motionlogger | Sleep | Wrist or leg | 3 days | NR | 84.85 | NR |
| Crowe, 2021 | Australia | Cross-sectional study | 4,408 (5-12 years, 49.88 per cent male, no diagnosis) | ActiGraph GT3X+ | Physical activity | Waist | 1 hour | NR | 81.99 | NR |
| Woods, 2018 | USA | NR | 179 (9-11 years old, 52.51 per cent male, no diagnosis) | ActiGraph wGT3X+ | Physical activity | Waist | 12 and 25 minutes | NR | 100 | NR |
| Noonan, 2019 | United Kingdom | Cross-sectional study | 401 (M = 12.18, 52.12 per cent male, no diagnosis) | SenseWear Armband Mini | Physical activity | Upper arm | 3 days | NR | 88.03 | NR |
| Ovans, 2018 | USA | Pilot Study | 20 (M = 11.47 (3.33), 66.67 per cent male,  Caucasian: 13 (86%) African American/black: 1 (7%) Asian/Pacific Islander: 1 (7%), oncology diagnosis) | Fitbit Flex | Physical activity | Wrist | 84 days | NR | 65 | NR |
| Quirk, 2020 | United Kingdom | Mixed-methods study | 13 (M = 8.9 (1.5), 61.54 per cent male, endocrine diagnosis) | RunScribe | Physical activity | Wrist | 3 days | NR | 91.00 | NR |
| Roth, 2019 | Israel | Cross-sectional study | 59 (M = 11.28, 57.63 per cent male, neurodevelopmental disorder) | Ambulatory Monitoring | Sleep | Wrist | 7 days | NR | 91.53 | NR |
| Esbensen, 2018 | NR | Pilot study | 30 (M = 11.68 (2.73), 60 per cent male,  Caucasian (93%; 7% African American), neurodevelopmental disorder) | Ambulatory Monitoring Micro-mini Motionlogger Actigraph | Sleep | NR | 7 days | NR | NR | 6.4 nights (1.1) |
| Belcher, 2021 | USA | Multistage probability design | 18,596 (3-80 years, 49.5 per cent male, Male 6-11 yrs Non-Hispanic white 52.8 (3.1) Non-Hispanic Black 12.9 (1.5) Mexican American 17.1 (2.5) Other Hispanic 8.4 (1.6) Other Race/Multi-Racial 8.7 (1.2) Female 6-11 yrs Non-Hispanic white 51.9 (3.4) Non-Hispanic Black 13.7 (1.7) Mexican American 16.7 (2.3) Other Hispanic 7.3 (1.1) Other Race/Multi-Racial 10.4 (1.4), no diagnosis) | ActiGraph GT3X+ | Physical activity | Wrist | 7 days | 1 day | 79.08 | 6-11 yrs male = 4.2 (0.1) 6-11 yrs female = 4.9 (0.1) |
| Yu-Ling Chen, 2021 | United Kingdom | Pilot cluster randomised-control trial | 176 (M = 9.3 (0.5), 56 per cent male, South Asian heritage 85 (48%) White British 63 (36%), no diagnosis) | PAL Technologies activPAL micro accelerometer  ActiGraph GT3X+ | Physical activity | activPAL3 = Leg ActiGraph GT3X = Waist | 7 days | 1 day T baseline and follow-up | activPal = 61.36 Actigraph = 82.39 | NR |
| Bartholomew, 2018 | USA | Cluster randomised-control trial | 2716 (9-10 years, 45.90 per cent male, Hispanic overall 31.99% int 33.06% con 29.51%  African American overall 9.51% int 9.73% con 8.97% White overall 46.26% int 46.54% con 45.60%, no diagnosis) | Actigraph GT3X+ | Physical activity | Waist | 5 days | NR | 91.80 | NR |
| Burford, 2022 | USA | Pilot study | 295 (M = 9 (1.2), 50.60 per cent male, Hispanic or Latino = 24.1% Not Hispanic or Latino = 75.4, no diagnosis) | Actigraph GTX3X+ | Physical activity | Waist | 5 days | NR | 68.14 | NR |
| Giddens, 2022 | USA | Longitudinal study | 5,189 (9 – 13 years, 51 per cent male, 111 (2.6%) identified as Asian, 353 as Black (8.4%), 811 (19.2%) as Hispanic, 416 (9.8%) as Other, and 2,516 (60.0%) as White, no diagnosis) | Fitbit Charge HR | Sleep | NR | NR | 7 days | 81.08 | 15.49 (5.59) |
| Grant, 2020 | USA | Observational study | 37 (11-14 years, 41.6% per cent male, 100% American Indian, no diagnosis) | Philips Respironics Actical | Physical activity and sleep | Wrist | 7 days | NR | 97.3 | NR |
| Hartikainen, 2022 | Finland | NR | 206 (M = 9.3 (0.3) - 11.2 (0.3), 42-61 per cent male, no diagnosis) | UKK Terveyspalvelut Oy, Tampere, Finland RM42 accelerometer | Physical activity | Waist | 5 days | NR | 95.63 | NR |
| Lecarie, 2022 | USA | Longitudinal twin study | 530 (M = 8.41 (0.69), 48.30 per cent male, 66.3% white; 33.7% Hispanic, no diagnosis) | Ambulatory Monitoring Motion Logger Micro Watch | Sleep | Wrist | 7 days | NR | 91.60 | 6.81 (0.67) |
| Breitenstein, 2021 | USA | Longitudinal twin study | 608 (M = 8.52 (0.63), 49.20 per cent male, 56.6% non-Hispanic European American, 24.8% Hispanic/Latino, 3.6% Asian American, 4.0% African American, 2.6% Native American, 1.0% Native Hawaiian families, and 8.0% multiethnic or unknown ethnicity, no diagnosis) | Ambulatory Monitoring Micro Motionlogger | Sleep | Wrist | 8 days | NR | 87.30 | 6.83 (0.62) |
| Finkelstein, 2020 | Singapore | Randomised controlled trial | 316 (M = 9.0 (1.4), 54.8 per cent male, Chinese 238 (75.3) Malay 4 (1.3 Indian 62 (19.6) Other 12 (3.8), no diagnosis) | Fitbit Zip® ActiGraph GT3X+ or wGT3X-BT | Physical activity | Fitbit zip = NR  GT3X+ or wGT3X-BT = waist | Fitbit zip = 48 weeks  Actigraph = 7 days (baseline, 6 and 12 month follow up) | GT3X+ wGT3X-BT ActiGraph = 4 days | Child-based Incentive Arm Baseline = 100  Family-based Incentive Arm Baseline = 100 | NR |
| Merbler, 2018 | USA | Feasibility study | 13 (M = 9. 5, 0 per cent male, 100% caucasian, neurodevelopmental disorder) | Philips Respironics Actiwatch 2 | Sleep | Wrist (exp one participant <2 years who wore on ankle per guidance for wearing actigraphs in toddlers) | 7 days | NR | 100 | NR |
| Bergqvist-Norén, 2022 | Sweden | Longitudinal study | 172 (5 years (0.07), 48.10 per cent male, no diagnosis) | Actigraph GT3X | Physical activity | Wrist | 7 days | PA = 4 days Sleep = 5 days | 5 years = 41.28 6 years = 52.91 | 6.74 |
| Rast, 2022 | Switzerland | NR | 43 (M = Upper limb group = 11.9  Wheelchair 11.7 Walking = 11.9  Overall sample: 65.12 per cent male, Neuromotor/Muscular skeletal diagnosis) | ZurichMOVE sensor modules | Physical activity | Upper body = each wrist Wheelchair = additional sensors on the trunk and the thigh sensor on the spokes of their wheelchair. Walking = additional sensor on the ankle of their less-affected leg, and, if applicable,ifixated sensors on their walking aids | 7 days | NR | 86.05 | NR |
| Løndal, 2020 | Norway | Mixed methods study | 42 (Median 6.5 (1.25), 52.38 per cent male, no diagnosis) | ActiGraph GT3X | Physical activity | NR | < 1 day | NR | 100.00 | NR |
| Lokhandwala, 2021 | USA | Within-subjects design | 19 (M = 4.75 (0.83), 73.68 per cent male, 75% White 18.8% Black 6.3% more than one race 18.8% Hispanic, no diagnosis) | Philips Respironics Actiwatch Spectrum Plus watch | Sleep | Wrist | 16 days (baseline and follow-up) | 3 days | 84.21 | Baseline = 12.3 (3.6) Follow-up = 11.3 (4.5) |
| Lindhiem, 2022 | USA | Pilot Study | 30 (Case M = 9.6 (1.6), 53 per cent male, White = 11 (73.3) Black or African American 1 (6.7), More than one race 1 (6.7) Chose not to answer 2 (13.3), neurodevelopmental disorder)  Con M = 10.1 (1.8), 40 per cent male, White 14 (93.3) Black or African American 1 (6.7) More than one race 0 (0) Chose not to answer 0 (0), no diagnosis) | Apple watch | Physical activity | NR | 2 days ADHD arm wore the device for at least 1 day when medication wasn't taken (e.g. weekend) | NR | 93.33 | NR |
| Kahn, 2020 | Chile | Case-crossover pilot study | 10 (M = 5.87 (5.34), 50 per cent male, atopic dermatitis diagnosis) | Condor Instruments, ActTrust | Sleep | Wrist or ankle for children who did not tolerate wrist actigraphy | 14 days | NR | 100 | NR |
| Johnstone, 2019 | United Kingdom | Feasibility cluster randomised controlled trial | 137 (Int M = 7.1, 47 per cent male (0.3) Con M = 7.0 (0.3), 38 per cent male, no diagnosis) | ActiGraph GT3X | Physical activity | Waist | 5 days | 3 days | 86.13 | Baseline = 4.5 Follow-up = 4.3 |
| Huertas-Delgado, 2021 | Spain | Quasi-experimental design | 366 (M = 8.05 (0.25), 54 per cent male, no diagnosis) | Actigraph wGT3X-BT | Physical activity | Waist | <1 day | NR | 96.99 | NR |
| Heikkila, 2022 | Finland | case-control study | 177 (Case M = 3.6 (1.8), 64 per cent male, 49% were from Asia, 37% from Africa, 6% from South America, and 8% from Eastern Europe  Con M = 4.8 (1.3), 53 per cent male, no diagnosis) | Activinsights GeneActiv Original | Sleep | Wrist | 7 days (at 2 time points 1 year apart) | NR | Case = 84.62 Con = 77.8 | NR |
| Salway, 2019 | United Kingdom | Cross-sectional study | 1296 (M = 10.9 (0.4), 48 per cent male, no diagnosis) | ActiGraph wGT3X-BT | Physical activity | NR | 5 days | 1 day | 94.75 | NR |
| Barnett, 2018 | Australia | NR | 276 (9-11 years, 47.83 per cent male, 58.54 % = Asian 41.46 % = European, no diagnosis) | Actigraph wGT3X-BT | Physical activity | NR | 8 days | NR | NR | Asian = 5.89 (2.25)  European = 5.45 (2.32) |
| Bartelink, 2019 | Netherlands | Longitudinal study | 1403 (M = 7.5, 47.40 per cent male, 94.1% = Western ethnicity, no diagnosis) | Actigraph GT3X+ | Physical activity | Waist | 7 days | 3 days | 81.50 | NR |
| Borghese, 2018 | Canada | Cross-sectional study | 50 (Development group M = 11.5 (1.1), 48 per cent male, no diagnosis)  Test group = 11.6 (1.1), 52 per cent male | Actical accelerometer Actiwatch 2 accelerometer | Physical activity and sleep | Actical accelerometer = waist Actiwatch 2 accelerometer = wrist | Actical accelerometer - 7 days and 8 nights  Actiwatch 2 accelerometer 8 nights | NR | ≥ 5 nights = 100 ≥ 6 nights = 98 | NR |
| Larocque Chevalier, 2020 | USA | Mixed methods design | 10 (M = 9.64 (1.47), 20 per cent male, 90 % white, other psychiatric) | ActiGraph GT9X | Sleep | Wrist | 7 weeks | NR | 90 | NR |
| Clevenger, 2022 | USA | Cross-sectional study | 738 (M = 8.98 (0.07), 50.20 per cent male, Mexican American 24.0 (3.6) Non-Hispanic White 40.8 (4.2) Other Hispanic 9.6 (1.3) Non-Hispanic Black 14.8 (2.5) Other race or multiracial 10.7 (2.1), no diagnosis) | ActiGraph GT3X+ | Physical activity | Wrist | 1 week | NR | 46.07 | NR |
| Kruizinga, 2021 | Netherlands | NR | 175 (M = 9.1 (4.3), 45.70 per cent male, Caucasian 92%  Other/mixed 8%, no diagnosis) | Withings Steel HR smartwatc | Physical activity and sleep | NR | 21 days | NR | Day = 99.43 Night = 98.29 | NR |
| Kruizinga, 2022 | Netherlands | Prospective cohort study | Study group = 90, Controlled asthma 67 per cent male, Uncontrolled asthma = 67 per cent male  CF = 47 per cent male  Con = 128, 46 per cent male  Overall sample = (9.7 - 11.1 years), Cardiovascualr/respirator diagnosis | Withings Steel HR smartwatc | Physical activity | NR | 28 days | NR | NR | NR |
| Jaser, 2021 | USA | Pilot randomized controlled trial | 41 (M = 8.0 (1.5), 36 per cent male, White, non-Hispanic = 29 (74%) Non-white = 8 (20%) White, Hispanic = 1 (3%) Not reported = 1 (3%), endocrine diagnosis) | Philips Actiwatch Spectrum PlusTM Actigraph wGT3X-BTTM | Sleep | NR | 7 days | NR | 85 | NR |
| Gotte, 2018 | Germany | Quasi-experimental study | Int = 24 (M = 14.5 (3.9), 62 per cent male  Con = 19 (M = 15.4 (3.7), 47 per cent male, oncology diagnosis) | Fitbit One Fitbit Flex | Physical activity | Fitbit One - pocket Fitbit Flex - wrist | Int = 6–8 weeks during treatment (T1) and for 2-weeks within 3 months after cessation of acute cancer treatment (T2). Con = 2 weeks after acute cancer treatment (T2). | NR | NR | NR |
| Ha, 2022 | Australia | Pilot study | 30 (M = 10.2 (1.5), 56 per cent male, oncology diagnosis) | Activinsights GENEActiv accelerometer Fossil Group Misfit Ray | Physical activity | GENEactive = Wrist Misfit = NR | GENEactive = 7 days (baseline and after the intervention) Misfit = 12 weeks | 3 days  Misfit = NR | GENEactive = 57.89 | NR |
| Leppänen, 2019 | Spain | Cross‐sectional study | 103 (M = 10.1 (1.1), 62 per cent male, no diagnosis) | ActiGraph GT3X+ | Physical activity | Wrist and waist concurrently | 7 days | 3 days | NR | Wrist 6.9 (0.5)  Waist 6.8 (0.5) |
| Rhodes, 2019 | Canada | Two-arm randomized trial | 102 (M = 8.93 (2.08), 48 per cent male, no diagnosis) | Actigraph GT3X | Physical activity | Waist | 7 days | 4 days | NR | NR |
| Rodríguez-Rodríguez, 2020 | Chile | Quasi-experimental study | 164 (M = 9.6 (1.8), Int = 72.86 per cent male, con = 58.33 per cent male, no diagnosis) | Actigraph wGTX3BT | Physical activity | Waist | NR | 8 hours | Intervention = 87.5% Control = 100% | NR |
| Willeboordse, 2022 | Netherlands | Longitudinal quasi-experimental design | 2374 | NR | NR | NR | NR | NR | 60.8 | NR |
| Jago, 2020 | United Kingdom | Longitudinal study | Phase 1: 958 (6 years, 51 per cent male) Phase 2: 662 (9 years, 45 per cent male)  Phase 3: (11 years, 48 per cent male All 3 phases: 512, no diagnosis) | ActiGraph wGT3X-BT | Physical activity | Waist | 5 days | 2 days | 90 with valid weekday data and 83 for weekend data for at least one time point | NR |
| Dishman, 2019 | USA | Prospective cohort study | 187 (M = 11 (0.5), 42.25 per cent male, 46% non Hispanic black, 28% non-Hispanic white, 9.6% Hispanic/Latino, 3.7% Asian/Pacific Islander, 2.7% American Indian, and 10% multi-racial, no diagnosis) | Actigraph GT1M GT3X | Physical activity | NR | 7 days | 4 days | 80 | NR |
| Dunton, 2022 | USA | Longitudinal cohort study. | 202 (M = 10.09 (0.89), 45.70 per cent male, Hispanic or Latino (57.6%), no diagnosis) | Actigraph GT3X | Physical activity | Waist | 7 days | 1 day (at 2 times points) | 83.66 | 26.11 (8.74) |
| Caillaud, 2022 | Australia | Randomised controlled trial | Int = (57, 49.12 per cent male  Con = (26, 38.46 per cent male  Overall sample: 10.4 (0.5), no diagnosis) | GENEActiv Misfit Ray | Physical activity | GENEActiv = wrist Misfit Ray = wrist | GENEActiv = 3 days before and after the intervention Misfit Ray = 5 weeks during the intervention | GENEActiv = 3 days  Misfit Ray = NR | 92.75 | NR |
| Verloigne, 2018 | Belgium | Cluster-randomised controlled trial | 57 (M = 10.5 (0.3), 50 per cent male, no diagnosis) | PAL Technologies activPAL | Physical activity | Leg | 5 days | 2 days | 63.16 | NR |
| Dalene, 2018 | Norway | Cross-sectional study | PANCS1 = 1306 9 years and 993 15 years PANCS2 = 1421 9 years and 1106 15 years  Overall sample: 9.6 (0.4) years and 15.3 (0.6), 9 years = 50.6 per cent male 15 years = 49.0 per cent male, no diagnosis) | PANCS1 = CSA 7164 ActiGraph PANCS2 = GT1M and GT3X+ ActiGraph | Physical activity | Waist | PANCS1 = 4 days PANCS2 = 7 days | 2 days | 86.76 | NR |
| Pedersen, 2022 | Denmark | Cluster-randomised controlled trial | 89 families (Int M = 8.6 (2.7) con M = 9.5 (2.5)  Overall sample: 45.30 per cent male, no diagnosis) | Axivity AX3 | Physical activity | One waist and one leg | 7 days (baseline and follow up) | 4 days | 96.51 | NR |
| Padmapriya, 2021 | Singapore | Longitudinal Study | 761 (Time 1 = 5.5 years, 52.26 per cent male, time 2: 8 years, 51.58 per cent male, no diagnosis) | ActiGraph GT3X+ | Physical activity | Wrist | 7 days | 3 days | 58.08 | NR |
| Verswijveren, 2022 | Australia | Cluster randomised controlled trial | 591 (M = 8.8 (0.4), 45 per cent male, no diagnosis) | ActiGraph GT3X | Physical activity | Waist | 8 days | 3 days | 46.70 | NR |
| Gantelius, 2022 | Sweden | Observational study | 11 (4–13 years, 54.55 per cent male, Neuromotor/Muscular skeletal diagnosis) | ActiGraph GT1M | Physical activity | Both wrists, the waist and right ankle | 4 days | NR | 54.55 | NR |
| Fishbein, 2018 | USA | Case-control study | 40 (case M = 11.0 (3.2), 65 per cent male, African American 5 (25%) Asian 4 (20) Latino 4 (20) White 6 (30) Other 1 (5), atopic dermatitis)  Con M = 11.5 (3.3, 65 per cent male, African American 4 (20) Asian 3 (15) Latino 5 (25) White 7 (35 Other 1 (5), no diagnosis) | Philips Respironics Actiwatch Spectrum device | Sleep | Wrist | NR | NR | 95 | Case = 6.4 (1.0) Control = 6.4 **(**1.1) |
| Goldschmidt, 2020 | USA | Pilot study | 40 (M = 11.8 (1.9), 45 per cent male, Non-Hispanic, White 15.4% Non-Hispanic, Black 64.1% Non-Hispanic, Other 2.5% Hispanic (all races) 18.0%, no diagnosis) | Phillips Bend Actiwatch 2 Respironics | Sleep | Wrist | 14 days | NR | NR | 11.2 |
| Harbottle, 2018 | United Kingdom | Proof-of-concept study | 12 Median = 14 years (range: 8-17), 50 per cent male, Neuromotor/Muscular skeletal diagnosis) | Aparito device | Physical activity | NR | 3 months | NR | NR | NR |
| Mackintosh, 2019 | Australia | Acceptability and Usability Study | 25 parents 36 children (7-12 years, 50 per cent male, no diagnosis) | X-Doria International KidFit | Physical activity and sleep | Wrist | 4 weeks | NR | NR | NR |
| Brazendale, 2019 | USA | NR | 20 (M = 9 (1.6), 50 per cent male, 90% non-Hispanic white, neurodevelopmental disorder) | Fitbit Alta HR | Heart rate | Wrist | NR | NR | NR | NR |
| Tracy, 2021 | USA | Behaviour change intervention trial | 610 (M = 5.03 (1.14), 51.50 per cent male, Hispanic/Latino (n = 361, 90.3%), non-Hispanic black (n = 24, 6.0%), non-Hispanic white (n = 9, 2.3%), and non-Hispanic multiracial (n = 6, 1.5%), no diagnosis) | ActiGraph GT3X+ | Sleep | NR | 7 days | 3 days | NR | 8.32 (1.74) |
| Crooks, 2021. | Australia | NR | 7-12 years  only 9-12 years were invited to use the accelerometer, no diagnosis) | ActiGraph wGT3X-BT | Physical activity | Waist | 7 days | 3 days | NR | boys 5.1 (1.7) girls 5.3 (1.6) |
| Duncan, 2018 | New Zealand | NR | 103 (8 years old, 55.80 per cent male, no diagnosis) | Axivity AX3 | Physical activity | One lower back (iliacF crest)  One right thigh | 7 days | NR | NR | NR |
| Gunn, 2019 | USA | Longitudinal study | 165 (M = 11.8 years (1.16), 48 per cent male, 78.6% Black/African American 15.7% White 5.7% Biracial, no diagnosis) | SenseWear Pro3 Armband™ device | Sleep | Upper arm | 7 days | 4 days | NR | NR |
| Choi, 2018 | Japan | NR | 13 (M = 8.0 (0.3), 23.07 per cent male, no diagnosis) | Kenz Lifecoder EX; Suzuken Co. Ltd | Sleep | Waist | 7 days | NR | NR | NR |
| Faghy, 2021 | United Kingdom | NR | 11 (M = 8.9 (1.3), 50.34 per cent male, no diagnosis) | ActivInsights GENEActiv Original accelerometer | Physical activity | Wrist | 7 days | NR | 100 | NR |
| Brazendale, 2018 | USA | NR | 30 (M = 7.2 (2.1), 63 per cent male, 87% non-Hispanic white, no diagnosis) | Fitbit Charge HR ActiGraph GT9X Link | Sleep | Wrist | 2 days | NR | 100 | NR |
| McWhannell, 2019 | United Kingdom | Exploratory Study | 53 (7-8 years, 49.06 per cent male, no diagnosis) | Actigraph GTX3+ | Physical activity | Waist | 15-minute period for 3 days | NR | NR | NR |
| Aadland, 2022 | Norway | Cross-sectional study | 376 (M = 2015-2016 = 4.7 (0.9), 52 per cent male 2017 = 6.3 (0.9), 51 per cent male  2018 = 7.3 (0.9), 51 per cent male  2019 = 8.3 (0.9), 51per cent male, no diagnosis) | ActiGraph GT3X+ | Physical activity | Waist | 14 days | 4 days | At least 2 timepoints 78  (had to have valid data to be included in the analysis) | Boys: 3 yrs old 12.9 (1.5)  4 years old 12.7 (1.8)  5 years old 12.3 (2.1)  6 years olds 12.6 (3.0)  7 years old 12.4 (2.8)  8 years old 12.1 (2.6) 9 years old = 12.4 (2.1) Girls: 12.0 (2.3) 3 = 12.7 (1.7) 4 = 12.8 (1.9) 5 = 12.6 (2.2) 6 = 12.3 (2.6)  7 = 12.6 (3.0) 8 = 11.8 (2.4) 9 = 11.8 (2.4) |
| Xiu, 2020 | Sweden | Longitudinal Study | 181 (2-6 years old, High risk: 54.69 per cent male, Low risk: 39.53 per cent male, no diagnosis) | ActiGraph GT3X+ | Sleep | Wrist | 7 days | 4 days | 84.11% | NR |
| Eichinger, 2018 | Germany | Cross-sectional study | 1134 (M = Weekend Day = 4.82 (0.73), 50.1 per cent male Weekday afternoon = 4.77 (0.75), 49.7 per cent male, no diagnosis) | Actiheart | Physical activity | NR | 6 days | 5 days | . | NR |
| Schmutz, 2020 | Switzerland | Cross-sectional study | 555 (M = Baseline - 3.9 (0.7) Follow-up 4.9 (0.7)  Overall sample: 47 per cent male, no diagnosis) | Actigraph wGT3X-BT | Physical activity | Waist | NR | 3 days | 89% | NR |
