# Supplementary material for: Adherence to Actigraphic Devices in Elementary School–Aged Children: Systematic Review and Meta-Analysis
Source: J Med Internet Res. 2025 Nov 3;27:e79718. doi: 10.2196/79718 (PMC12582557; doi:10.2196/79718)
Supplement: Multimedia Appendix 6 [file jmir-v27-e79718-s006.docx]

**Multimedia appendix 6. References included in the systematic review and meta-analysis**

1. Migueles, J. H., Cadenas-Sanchez, C., Esteban-Cornejo, I., Mora-Gonzalez, J., Rodriguez-Ayllon, M., Solis-Urra, P., Erickson, K. I., Kramer, A. F., Hillman, C. H., Catena, A., & Ortega, F. B. (2021). Associations of sleep with gray matter volume and their implications for academic achievement, executive function and intelligence in children with overweight/obesity. *Pediatric Obesity*, *16*(2), e12707. https://doi.org/10.1111/ijpo.12707
2. Fairclough, S. J., Taylor, S., Rowlands, A. V., Boddy, L. M., & Noonan, R. J. (2019). Average acceleration and intensity gradient of primary school children and associations with indicators of health and well-being. *Journal of Sports Sciences*, *37*(18), 2159–2167. <https://doi.org/10.1080/02640414.2019.1624313>
3. Beltran-Valls, M. R., Janssen, X., Farooq, A., Adamson, A. J., Pearce, M. S., Reilly, J. K., Basterfield, L., & Reilly, J. J. (2019). Longitudinal changes in vigorous intensity physical activity from childhood to adolescence: Gateshead Millennium Study. *Journal of Science and Medicine in Sport*, *22*(4), 450–455. <https://doi.org/10.1016/j.jsams.2018.10.010>
4. Pearce, A., Hope, S., Griffiths, L., Cortina-Borja, M., Chittleborough, C., & Law, C. (2019). What if all children achieved WHO recommendations on physical activity? Estimating the impact on socioeconomic inequalities in childhood overweight in the UK Millennium Cohort Study. *International Journal of Epidemiology*, *48*(1), 134–147. https://doi.org/10.1093/ije/dyy267
5. Silva, D. A. S., Chaput, J.-P., Katzmarzyk, P. T., Fogelholm, M., Hu, G., Maher, C., Olds, T., Onywera, V., Sarmiento, O. L., Standage, M., Tudor-Locke, C., & Tremblay, M. S. (2018). Physical Education Classes, Physical Activity, and Sedentary Behavior in Children. *Medicine and Science in Sports and Exercise*, *50*(5), 995–1004. https://doi.org/10.1249/MSS.0000000000001524
6. Adank, A. M., Kann, D. H. H. V., Remmers, T., Kremers, S. P. J., & Vos, S. B. (2021). Longitudinal Perspectives on Children’s Physical Activity Patterns: “Do Physical Education–Related Factors Matter?” *Journal of Physical Activity and Health*, *18*(10), 1199–1206. <https://doi.org/10.1123/jpah.2020-0859>
7. Alder, M. L., Johnson, C. R., Zauszniewski, J. A., Malow, B. A., Burant, C. J., & Scahill, L. (2023). Feasibility of Actigraphy for Evaluating Sleep and Daytime Physical Activity in Children with Autism Spectrum Disorder. *Journal of Autism and Developmental Disorders*, *53*(9), 3670–3682. <https://doi.org/10.1007/s10803-022-05661-5>
8. Allen, C. P., Telford, R. M., Telford, R., D., & Olive, L. S. (2019). Sport, physical activity and physical education experiences: Associations with functional body image in children. *Psychology of Sport and Exercise*, *45*, 101572. <https://doi.org/10.1016/j.psychsport.2019.101572>
9. Anselma, M., Altenburg, T. M., Twisk, J. W. R., Wang, X., & Chinapaw, M. J. M. (2023). How to Evaluate the Effectiveness of Health Promotion Actions Developed Through Youth-Centered Participatory Action Research. *Health Education & Behavior: The Official Publication of the Society for Public Health Education*, *50*(2), 199–210. <https://doi.org/10.1177/10901981211046533>
10. Bagley, E. J., Fuller-Rowell, T. E., Saini, E. K., Philbrook, L. E., & El-Sheikh, M. (2018). Neighborhood Economic Deprivation and Social Fragmentation: Associations with Children’ Sleep. *Behavioral Sleep Medicine*, *16*(6), 542–552. https://doi.org/10.1080/15402002.2016.1253011
11. Bedell, D., Sevcik, T., Daly, J. M., & Levy, B. T. (2022). Food Intake Compared to Exercise Association with Obesity in Children Ages 3-6. *Journal of the American Board of Family Medicine: JABFM*, *35*(6), 1072–1080. <https://doi.org/10.3122/jabfm.2022.220159R1>
12. Beemer, L. R., Twardzik, E., Colabianchi, N., & Hasson, R. E. (2020). Patterning Of Physical Activity And Sedentary Behavior At And Away From School In Preadolescent Children: 3626 Board #8 May 30 9:00 AM - 11:00 AM. *Medicine & Science in Sports & Exercise*, *52*(7S), 991. https://doi.org/10.1249/01.mss.0000686336.41211.65
13. Bejarano, C. M., Carlson, J. A., Conway, T. L., Saelens, B. E., Glanz, K., Couch, S. C., Cain, K. L., & Sallis, J. F. (2021). Physical Activity, Sedentary Time, and Diet as Mediators of the Association Between TV Time and BMI in Youth. *American Journal of Health Promotion: AJHP*, *35*(5), 613–623. <https://doi.org/10.1177/0890117120984943>
14. Bekelman, T. A., Sauder, K. A., Rockette-Wagner, B., Glueck, D. H., & Dabelea, D. (2021). Sociodemographic Predictors of Adherence to National Diet and Physical Activity Guidelines at Age 5 Years: The Healthy Start Study. *American Journal of Health Promotion*, *35*(4), 514–524. <https://doi.org/10.1177/0890117120968654>
15. Berge, J. M., Cheatom, O., Fertig, A. R., Tate, A., Trofholz, A., Brito, J. N., & Shippee, N. (2021). Examining the Relationship Between Parental Stress and Girls’ and Boys’ Physical Activity Among Racially/Ethnically Diverse and Immigrant/Refugee Populations. *Pediatric Exercise Science*, *33*(3), 97–102. https://doi.org/10.1123/pes.2020-0190
16. Bolger, L. E., Bolger, L. A., O’Neill, C., Coughlan, E., O’Brien, W., Lacey, S., & Burns, C. (2018). Accuracy of Children’s Perceived Skill Competence and its Association With Physical Activity. *Journal of Physical Activity & Health*, 1–8. https://doi.org/10.1123/jpah.2017-0371
17. Brønd, J. C., Aadland, E., Andersen, L. B., Resaland, G. K., Andersen, S. A., & Arvidsson, D. (2019). The ActiGraph counts processing and the assessment of vigorous activity. *Clinical Physiology and Functional Imaging*, *39*(4), 276–283. https://doi.org/10.1111/cpf.12571
18. Brudy, L., Hock, J., Häcker, A.-L., Meyer, M., Oberhoffer, R., Hager, A., Ewert, P., & Müller, J. (2020). Children with Congenital Heart Disease Are Active but Need to Keep Moving: A Cross-Sectional Study Using Wrist-Worn Physical Activity Trackers. *The Journal of Pediatrics*, *217*, 13–19. https://doi.org/10.1016/j.jpeds.2019.09.077
19. Caserta, A., Reedman, S., Morgan, P., & Williams, C. M. (2022). Physical activity and quality of life in children with idiopathic toe walking: A cross sectional study. *BMC Pediatrics*, *22*(1), 544. https://doi.org/10.1186/s12887-022-03583-w
20. Cassim, R., Dharmage, S. C., Peters, R. L., Koplin, J. J., Allen, K. J., Tang, M. L. K., Lowe, A. J., Olds, T. S., Fraysse, F., Milanzi, E., Russell, M. A., & HealthNuts Investigators. (2021). Are young children with asthma more likely to be less physically active? *Pediatric Allergy and Immunology: Official Publication of the European Society of Pediatric Allergy and Immunology*, *32*(2), 288–294. https://doi.org/10.1111/pai.13383
21. Chen, B., Waters, C. N., Compier, T., Uijtdewilligen, L., Petrunoff, N. A., Lim, Y. W., van Dam, R., & Müller-Riemenschneider, F. (2020). Understanding physical activity and sedentary behaviour among preschool-aged children in Singapore: A mixed-methods approach. *BMJ Open*, *10*(4), e030606. https://doi.org/10.1136/bmjopen-2019-030606
22. Christian, D. L., Todd, C., Rance, J., Stratton, G., Mackintosh, K. A., Rapport, F., & Brophy, S. (2020). Involving the headteacher in the development of school-based health interventions: A mixed-methods outcome and process evaluation using the RE-AIM framework. *PloS One*, *15*(4), e0230745. https://doi.org/10.1371/journal.pone.0230745
23. Clark, A. F., Wilk, P., & Gilliland, J. A. (2019). Comparing Physical Activity Behavior of Children During School Between Balanced and Traditional School Day Schedules. *The Journal of School Health*, *89*(2), 129–135. https://doi.org/10.1111/josh.12722
24. Costa, B. G. G. da, Silva, K. S. da, Silveira, P. M. da, Berria, J., Machado, A. R., & Petroski, E. L. (2019). The effect of an intervention on physical activity of moderate-and-vigorous intensity, and sedentary behavior during adolescents’ time at school. *Revista Brasileira De Epidemiologia = Brazilian Journal of Epidemiology*, *22*, e190065. https://doi.org/10.1590/1980-549720190065
25. Cradock, A. L., Barrett, J. L., Taveras, E. M., Peabody, S., Flax, C. N., Giles, C. M., & Gortmaker, S. L. (2019). Effects of a before-school program on student physical activity levels. *Preventive Medicine Reports*, *15*, 100940. https://doi.org/10.1016/j.pmedr.2019.100940
26. Draper, C. E., Tomaz, S. A., Jones, R. A., Hinkley, T., Twine, R., Kahn, K., & Norris, S. A. (2019). Cross-sectional associations of physical activity and gross motor proficiency with adiposity in South African children of pre-school age. *Public Health Nutrition*, *22*(4), 614–623. https://doi.org/10.1017/S1368980018003579
27. Duck, A. A., Hall, K. C., Klamm, M., Temple, M., & Robinson, J. C. (2021). Physical activity and fitness: The feasibility and preliminary effectiveness of wearable activity tracker technology incorporating altruistic motivation in youth. *Journal for Specialists in Pediatric Nursing*, *26*(1), e12313. https://doi.org/10.1111/jspn.12313
28. Evenson, K. R., Arredondo, E. M., Carnethon, M. R., Delamater, A. M., Gallo, L. C., Isasi, C. R., Perreira, K. M., Foti, S. A., Van Horn, L., Vidot, D. C., & Sotres-Alvarez, D. (2019). Physical Activity and Sedentary Behavior among US Hispanic/Latino Youth: The SOL Youth Study. *Medicine and Science in Sports and Exercise*, *51*(5), 891–899. https://doi.org/10.1249/MSS.0000000000001871
29. Fang, C., Zhang, J., Zhou, T., Li, L., Lu, Y., Gao, Z., & Quan, M. (2020). Associations between Daily Step Counts and Physical Fitness in Preschool Children. *Journal of Clinical Medicine*, *9*(1), 163. https://doi.org/10.3390/jcm9010163
30. Fraysse, F., Grobler, A. C., Muller, J., Wake, M., & Olds, T. (2019). Physical activity and sedentary activity: Population epidemiology and concordance in Australian children aged 11–12 years and their parents. *BMJ Open*, *9*(Suppl 3). https://doi.org/10.1136/bmjopen-2018-023194
31. Gaser, D., Peters, C., Götte, M., Oberhoffer-Fritz, R., Feuchtinger, T., Schmid, I., von Luettichau, I., & Kesting, S. (2022). Analysis of self-reported activities of daily living, motor performance and physical activity among children and adolescents with cancer: Baseline data from a randomised controlled trial assessed shortly after diagnosis of leukaemia or non-Hodgkin lymphoma. *European Journal of Cancer Care*, *31*(2), e13559. <https://doi.org/10.1111/ecc.13559>
32. Gerber, M., Lang, C., Beckmann, J., Degen, J., du Randt, R., Gall, S., Long, K. Z., Müller, I., Nienaber, M., Steinmann, P., Pühse, U., Utzinger, J., Nqweniso, S., & Walter, C. (2021). Associations Between Household Socioeconomic Status, Car Ownership, Physical Activity, and Cardiorespiratory Fitness in South African Primary Schoolchildren Living in Marginalized Communities. *Journal of Physical Activity & Health*, *18*(8), 883–894. https://doi.org/10.1123/jpah.2020-0839
33. Hall, C. J. S., Eyre, E. L. J., Oxford, S. W., & Duncan, M. J. (2019). Does Perception of Motor Competence Mediate Associations between Motor Competence and Physical Activity in Early Years Children? *Sports (Basel, Switzerland)*, *7*(4), 77. https://doi.org/10.3390/sports7040077
34. Holzhausen, E. A., Hagen, E. W., LeCaire, T., Cadmus-Bertram, L., Malecki, K. C., & Peppard, P. E. (2020). A Comparison of Self- and Proxy-Reported Subjective Sleep Durations With Objective Actigraphy Measurements in a Survey of Wisconsin Children 6–17 Years of Age. *American Journal of Epidemiology*, *190*(5), 755–765. https://doi.org/10.1093/aje/kwaa254
35. Hulst, R. Y., Gorter, J. W., Obeid, J., Voorman, J. M., van Rijssen, I. M., Gerritsen, A., Visser‐Meily, J. M. A., Pillen, S., & Verschuren, O. (2023). Accelerometer‐measured physical activity, sedentary behavior, and sleep in children with cerebral palsy and their adherence to the 24‐hour activity guidelines. *Developmental Medicine and Child Neurology*, *65*(3), 393–405. https://doi.org/10.1111/dmcn.15338
36. Joensuu, L., Syväoja, H., Kallio, J., Kulmala, J., Kujala, U. M., & Tammelin, T. H. (2018). Objectively measured physical activity, body composition and physical fitness: Cross-sectional associations in 9- to 15-year-old children. *European Journal of Sport Science*, *18*(6), 882–892. https://doi.org/10.1080/17461391.2018.1457081
37. Kattelmann, K. K., Meendering, J. R., Hofer, E. J., Merfeld, C. M., Olfert, M. D., Hagedorn, R. L., Colby, S. E., Franzen-Castle, L., Moyer, J., Mathews, D. R., & White, A. A. (2019). The iCook 4-H Study: Report on Physical Activity and Sedentary Time in Youth Participating in a Multicomponent Program Promoting Family Cooking, Eating, and Playing Together. *Journal of Nutrition Education and Behavior*, *51*(3S), S30–S40. https://doi.org/10.1016/j.jneb.2018.09.002
38. Kippe, K., Marques, A., Martins, J., & Lagestad, P. A. (2022). Parents’ Inadequate Estimate of Their Children’s Objectively Physical Activity Level. *Children*, *9*(3), 392. https://doi.org/10.3390/children9030392
39. Knox, E., Glazebrook, C., Randell, T., Leighton, P., Guo, B., Greening, J., Davies, E. B., Amor, L., & Blake, H. (2019). SKIP (Supporting Kids with diabetes In Physical activity): Feasibility of a randomised controlled trial of a digital intervention for 9-12 year olds with type 1 diabetes mellitus. *BMC Public Health*, *19*(1), 371. https://doi.org/10.1186/s12889-019-6697-1
40. Li, R., Liang, X., Liu, F., Zhou, Z., Zhang, Z., Lu, Y., Wang, P., & Yang, B. (2021). Mediating Effect of Motor Competence on the Relationship between Physical Activity and Quality of Life in Children with Attention Deficit Hyperactivity Disorder. *BioMed Research International*, *2021*, 4814250. https://doi.org/10.1155/2021/4814250
41. Ludwig, K., & Rauch, W. A. (2018). Associations between physical activity, positive affect, and self-regulation during preschoolers’ everyday lives. *Mental Health and Physical Activity*, *15*, 63–70. https://doi.org/10.1016/j.mhpa.2018.07.002
42. Mazza, S., Bastuji, H., & Rey, A. E. (2020). Objective and Subjective Assessments of Sleep in Children: Comparison of Actigraphy, Sleep Diary Completed by Children and Parents’ Estimation. *Frontiers in Psychiatry*, *11*, 495. https://doi.org/10.3389/fpsyt.2020.00495
43. Mccrorie, P., Mitchell, R., & Ellaway, A. (2018). Comparison of two methods to assess physical activity prevalence in children: An observational study using a nationally representative sample of Scottish children aged 10–11 years. *BMJ Open*, *8*(1), Article 1. https://doi.org/10.1136/bmjopen-2017-018369
44. Riiser, K., Richardsen, K. R., Haugen, A. L. H., Lund, S., & Løndal, K. (2020). Active play in ASP –a matched-pair cluster-randomized trial investigating the effectiveness of an intervention in after-school programs for supporting children’s physical activity. *BMC Public Health*, *20*(1), 500. https://doi.org/10.1186/s12889-020-08645-1
45. Hartman, A. G., Terhorst, L., Little, N., & Bendixen, R. M. (2020). Uncovering sleep in young males with Duchenne muscular dystrophy. *European Journal of Paediatric Neurology: EJPN: Official Journal of the European Paediatric Neurology Society*, *26*, 20–28. https://doi.org/10.1016/j.ejpn.2020.02.012
46. Gråstén, A., & Yli-Piipari, S. (2019). The Patterns of Moderate to Vigorous Physical Activity and Physical Education Enjoyment Through a 2-Year School-Based Program. *The Journal of School Health*, *89*(2), 88–98. https://doi.org/10.1111/josh.12717
47. Gråstén, A., Huhtiniemi, M., Hakonen, H., & Jaakkola, T. (2021). Development of accelerometer-based light to vigorous physical activity in fitness profiles of school-aged children. *Scandinavian Journal of Medicine & Science in Sports*, *31*(12), 2343–2355. https://doi.org/10.1111/sms.14056
48. Wiersma, R., Lu, C., Hartman, E., & Corpeleijn, E. (2019). Physical activity around the clock: Objectively measured activity patterns in young children of the GECKO Drenthe cohort. *BMC Public Health*, *19*(1), 1647. https://doi.org/10.1186/s12889-019-7926-3
49. Williams, R. A., Dring, K. J., Morris, J. G., Sun, F.-H., & Cooper, S. B. (2022). Agreement and equivalence of estimated physical activity behaviours, using ENMO- and counts-based processing methods, for wrist-worn accelerometers in adolescents. *Journal of Sports Sciences*. https://www.tandfonline.com/doi/abs/10.1080/02640414.2023.2167254
50. Verjans-Janssen, S. R. B., Gerards, S. M. P. L., Kremers, S. P. J., Vos, S. B., Jansen, M. W. J., & Van Kann, D. H. H. (2020). Effects of the KEIGAAF intervention on the BMI z-score and energy balance-related behaviors of primary school-aged children. *The International Journal of Behavioral Nutrition and Physical Activity*, *17*(1), 105. https://doi.org/10.1186/s12966-020-01012-8
51. Chan, C. H. S., Ha, A. S. C., Ng, J. Y. Y., & Lubans, D. R. (2019). Associations between fundamental movement skill competence, physical activity and psycho-social determinants in Hong Kong Chinese children. *Journal of Sports Sciences*, *37*(2), 229–236. https://doi.org/10.1080/02640414.2018.1490055
52. St Laurent, C. W., Holmes, J. F., & Spencer, R. M. C. (2022). Temporal Associations between Actigraphy-Measured Daytime Movement Behaviors and Nap Sleep in Early Childhood. *International Journal of Environmental Research and Public Health*, *19*(22), 15308. https://doi.org/10.3390/ijerph192215308
53. Wang, T., Qian, Y., Zhong, T., & Qi, J. (2022). Associations between Fundamental Movement Skills and Moderate-to-Vigorous Intensity Physical Activity among Chinese Children and Adolescents with Intellectual Disability. *International Journal of Environmental Research and Public Health*, *19*(20), 13057. https://doi.org/10.3390/ijerph192013057
54. Yu, J. J., Capio, C. M., Abernethy, B., & Sit, C. H. P. (2021). Moderate-to-vigorous physical activity and sedentary behavior in children with and without developmental coordination disorder: Associations with fundamental movement skills. *Research in Developmental Disabilities*, *118*, 104070. https://doi.org/10.1016/j.ridd.2021.104070
55. Velde, G. T., Plasqui, G., Willeboordse, M., Winkens, B., & Vreugdenhil, A. (2021). Associations between physical activity, sedentary time and cardiovascular risk factors among Dutch children. *PloS One*, *16*(8), e0256448. https://doi.org/10.1371/journal.pone.0256448
56. Aguilar-Farias, N., Martino-Fuentealba, P., & Chandia-Poblete, D. (2020). Correlates of device-measured physical activity, sedentary behaviour and sleeping in children aged 9-11 years from Chile: ESPACIOS study (Factores asociados con actividad física, conducta sedentaria y sueño medidos con acelerómetros en niños de 9-11 años. *Retos*, *37*, 1–10. https://doi.org/10.47197/retos.v37i37.71142
57. Clevenger, K. A., Brønd, J. C., Mackintosh, K. A., Pfeiffer, K. A., Montoye, A. H. K., & McNarry, M. A. (2022). Impact of ActiGraph sampling rate on free-living physical activity measurement in youth. *Physiological Measurement*, *43*(10). https://doi.org/10.1088/1361-6579/ac944f
58. Kjellberg Olofsson, C., Skovdahl, P., Fridolfsson, J., Arvidsson, D., Börjesson, M., Sunnegårdh, J., & Buratti, S. (2023). Life satisfaction, health-related quality of life and physical activity after treatment for valvular aortic stenosis. *Cardiology in the Young*, *33*(3), 403–409. https://doi.org/10.1017/S1047951122000920
59. Callaghan, S., Morrison, M. L., McKeown, P. P., Tennyson, C., Sands, A. J., McCrossan, B., Grant, B., Craig, B. G., & Casey, F. A. (2021). Exercise prescription improves exercise tolerance in young children with CHD: A randomised clinical trial. *Open Heart*, *8*(1), e001599. https://doi.org/10.1136/openhrt-2021-001599
60. Oakley, J., Peters, R. L., Wake, M., Grobler, A. C., Kerr, J. A., Lycett, K., Cassim, R., Russell, M., Sun, C., Tang, M. L. K., Koplin, J. J., & Mavoa, S. (2021). Backyard benefits? A cross-sectional study of yard size and greenness and children’s physical activity and outdoor play. *BMC Public Health*, *21*(1), 1402. https://doi.org/10.1186/s12889-021-11475-4
61. Palmer, C. A., Clementi, M. A., Meers, J. M., & Alfano, C. A. (2018). Co-Sleeping among School-Aged Anxious and Non-Anxious Children: Associations with Sleep Variability and Timing. *Journal of Abnormal Child Psychology*, *46*(6), 1321–1332. https://doi.org/10.1007/s10802-017-0387-1
62. Dahlgren, A., Sjöblom, L., Eke, H., Bonn, S. E., & Trolle Lagerros, Y. (2021). Screen time and physical activity in children and adolescents aged 10-15 years. *PloS One*, *16*(7), e0254255. https://doi.org/10.1371/journal.pone.0254255
63. Skjåkødegård, H. F., Danielsen, Y. S., Frisk, B., Hystad, S. W., Roelants, M., Pallesen, S., Conlon, R. P. K., Wilfley, D. E., & Juliusson, P. B. (2021). Beyond sleep duration: Sleep timing as a risk factor for childhood obesity. *Pediatric Obesity*, *16*(1), e12698. https://doi.org/10.1111/ijpo.12698
64. Harrex, H. A. L., Skeaff, S. A., Black, K. E., Davison, B. K., Haszard, J. J., Meredith-Jones, K., Quigg, R., Saeedi, P., Stoner, L., Wong, J. E., & Skidmore, P. M. L. (2018). Sleep timing is associated with diet and physical activity levels in 9-11-year-old children from Dunedin, New Zealand: The PEDALS study. *Journal of Sleep Research*, *27*(4), e12634. https://doi.org/10.1111/jsr.12634
65. Park, J., Ishikawa-Takata, K., Lee, S., Kim, E., Lim, K., Kim, H., Lee, I.-S., & Tanaka, S. (2018). Comparison of daily physical activity parameters using objective methods between overweight and normal-weight children. *Journal of Sport and Health Science*, *7*(2), 210–217. https://doi.org/10.1016/j.jshs.2017.01.008
66. Van Kann, D. H. H., Adank, A. M., van Dijk, M. L., Remmers, T., & Vos, S. B. (2019). Disentangling Physical Activity and Sedentary Behavior Patterns in Children with Low Motor Competence. *International Journal of Environmental Research and Public Health*, *16*(20), 3804. https://doi.org/10.3390/ijerph16203804
67. Chen, H., Wang, L., Xin, F., Liang, G., & Zhou, Y. (2023). Associations between 24-h movement behaviours and BMI in Chinese primary- and middle- school students. *Journal of Exercise Science & Fitness*, *21*(2), 186–192. https://doi.org/10.1016/j.jesf.2023.01.002
68. Chong, K. H., Parrish, A.-M., Cliff, D. P., Dumuid, D., & Okely, A. D. (2021). Cross-Sectional and Longitudinal Associations between 24-Hour Movement Behaviours, Recreational Screen Use and Psychosocial Health Outcomes in Children: A Compositional Data Analysis Approach. *International Journal of Environmental Research and Public Health*, *18*(11), 5995. https://doi.org/10.3390/ijerph18115995
69. Talarico, R., & Janssen, I. (2018). Compositional associations of time spent in sleep, sedentary behavior and physical activity with obesity measures in children. *International Journal of Obesity (2005)*, *42*(8), 1508–1514. https://doi.org/10.1038/s41366-018-0053-x
70. Herbert, J., Matłosz, P., Martínez-Rodríguez, A., Przednowek, K., Asif, M., & Wyszyńska, J. (2022). Weekday and Weekend Physical Activity of Preschool Children in Relation to Selected Socioeconomic Indicators. *International Journal of Environmental Research and Public Health*, *19*(9), 4999. https://doi.org/10.3390/ijerph19094999
71. Salin, K., Huhtiniemi, M., Watt, A., Hakonen, H., & Jaakkola, T. (2019). Differences in the Physical Activity, Sedentary Time, and BMI of Finnish Grade 5 Students. *Journal of Physical Activity & Health*, *16*(9), 765–771. <https://doi.org/10.1123/jpah.2018-0622>
72. Li, L., Sheehan, C. M., Valiente, C., Eisenberg, N., Doane, L. D., Spinrad, T. L., Johns, S. K., Diaz, A., Berger, R. H., & Southworth, J. (2021). Similarities and differences between actigraphy and parent-reported sleep in a Hispanic and non-Hispanic White sample. *Sleep Medicine*, *83*, 160–167. https://doi.org/10.1016/j.sleep.2021.04.036
73. Lai, L., Cai, L., Tan, W., Zeng, X., Sun, F., Huang, W. Y., Wong, S. H. S., & Chen, Y. (2020). Adiposity Mediates the Association of Objectively Measured Physical Activity with Cardiorespiratory Fitness in Children. *Childhood Obesity (Print)*, *16*(8), 554–563. https://doi.org/10.1089/chi.2020.0009
74. Willoughby, M. T., Wylie, A. C., & Catellier, D. J. (2018). Testing the association between physical activity and executive function skills in early childhood. *Early Childhood Research Quarterly*, *44*, 82–89. https://doi.org/10.1016/j.ecresq.2018.03.004
75. Wyszyńska, J., Matłosz, P., Asif, M., Szybisty, A., Lenik, P., Dereń, K., Mazur, A., & Herbert, J. (2021). Association between objectively measured body composition, sleep parameters and physical activity in preschool children: A cross-sectional study. *BMJ Open*, *11*(1), e042669. https://doi.org/10.1136/bmjopen-2020-042669
76. Xu, W. H., & Qi, J. (2022). Meeting 24-hour movement guidelines: Their relationships with overweight and obesity among Chinese children with autism spectrum disorder. *Research in Autism Spectrum Disorders*, *99*, 102066. https://doi.org/10.1016/j.rasd.2022.102066
77. Yang, X., Jago, R., Zhai, Y., Yang, Z. Y., Wang, Y. Y., Si, X., Wang, J., Gao, J. F., Chen, J. R., Yu, Y. J., & Zhao, W. H. (2019). Validity and Reliability of Chinese Physical Activity Questionnaire for Children Aged 10-17 Years. *Biomedical and Environmental Sciences: BES*, *32*(9), 647–658. https://doi.org/10.3967/bes2019.084
78. Yoong, S. L., Grady, A., Stacey, F., Polimeni, M., Clayton, O., Jones, J., Nathan, N., Wyse, R., & Wolfenden, L. (2019). A pilot randomized controlled trial examining the impact of a sleep intervention targeting home routines on young children’s (3-6 years) physical activity. *Pediatric Obesity*, *14*(4), e12481. https://doi.org/10.1111/ijpo.12481
79. Zhang, B., Liu, Y., Zhao, M., Meng, X., Deng, Y., Zheng, X., Wang, X., Xiong, S., & Han, Y. (2020). Differential effects of acute physical activity on executive function in preschoolers with high and low habitual physical activity levels. *Mental Health and Physical Activity*, *18*, 100326. https://doi.org/10.1016/j.mhpa.2020.100326
80. So, C. J., Gallagher, M. W., Palmer, C. A., & Alfano, C. A. (2021). Prospective associations between pre-sleep electronics use and same-night sleep in healthy school-aged children. *Children’s Health Care: Journal of the Association for the Care of Children’s Health*, *50*(3), 293–310. https://doi.org/10.1080/02739615.2021.1890078
81. Sprengeler, O., Hebestreit, A., Gohres, H., Bucksch, J., & Buck, C. (2020). Effects of Installing Height-Adjustable Standing Desks on Daily and Domain-Specific Duration of Standing, Sitting, and Stepping in 3rd Grade Primary School Children. *Frontiers in Public Health*, *8*, 396. https://doi.org/10.3389/fpubh.2020.00396
82. Abdollahi, A. M., Li, X., Merikanto, I., Leppänen, M. H., Vepsäläinen, H., Lehto, R., Ray, C., Erkkola, M., & Roos, E. (2024). Comparison of actigraphy-measured and parent-reported sleep in association with weight status among preschool children. *Journal of Sleep Research*, *33*(1), e13960. https://doi.org/10.1111/jsr.13960
83. Downing, K. L., Hinkley, T., Timperio, A., Salmon, J., Carver, A., Cliff, D. P., Okely, A. D., & Hesketh, K. D. (2021). Volume and accumulation patterns of physical activity and sedentary time: Longitudinal changes and tracking from early to late childhood. *International Journal of Behavioral Nutrition and Physical Activity*, *18*(1), 39. https://doi.org/10.1186/s12966-021-01105-y
84. Mücke, M., Ludyga, S., Andrä, C., Gerber, M., & Herrmann, C. (2021). Associations between physical activity, basic motor competencies and automatic evaluations of exercise. *Journal of Sports Sciences*, *39*(16), 1903–1909. https://doi.org/10.1080/02640414.2021.1907902
85. Beunders, V. A. A., Koopman-Verhoeff, M. E., Vermeulen, M. J., Jansen, P. W., Luik, A. I., Derks, I. P. M., Reiss, I. K. M., Joosten, K. F. M., & Jaddoe, V. W. V. (2023). Sleep, 24-hour activity rhythms, and cardiometabolic risk factors in school-age children. *Journal of Clinical Sleep Medicine: JCSM: Official Publication of the American Academy of Sleep Medicine*, *19*(7), 1219–1229. https://doi.org/10.5664/jcsm.10544
86. McGarty, A., Jones, N., Rutherford, K., Westrop, S., Sutherland, L., Jahoda, A., & Melville, C. (2021). Feasibility of the Go2Play Active Play intervention for increasing physical and social development in children with intellectual disabilities. *Pilot and Feasibility Studies*, *7*(1), 43. https://doi.org/10.1186/s40814-021-00783-6
87. McMullen, J. A., McCrindle, B. W., Dell, S. D., Feldman, B. M., & Longmuir, P. E. (2019). Understanding parent perceptions of healthy physical activity for their child with a chronic medical condition: A cross-sectional study. *Paediatrics & Child Health*, *24*(3), e135–e141. https://doi.org/10.1093/pch/pxy122
88. Mughal, R., Hill, C. M., Joyce, A., & Dimitriou, D. (2020). Sleep and Cognition in Children with Fetal Alcohol Spectrum Disorders (FASD) and Children with Autism Spectrum Disorders (ASD). *Brain Sciences*, *10*(11), 863. https://doi.org/10.3390/brainsci10110863
89. Nakabazzi, B., Wachira, L.-J. M., Oyeyemi, A. L., Ssenyonga, R., & Onywera, V. O. (2020). Prevalence and socio-demographic correlates of accelerometer measured physical activity levels of school-going children in Kampala city, Uganda. *PloS One*, *15*(7), e0235211. https://doi.org/10.1371/journal.pone.0235211
90. Nathan, N. K., Sutherland, R. L., Hope, K., McCarthy, N. J., Pettett, M., Elton, B., Jackson, R., Trost, S. G., Lecathelinais, C., Reilly, K., Wiggers, J. H., Hall, A., Gillham, K., Herrmann, V., & Wolfenden, L. (2020). Implementation of a School Physical Activity Policy Improves Student Physical Activity Levels: Outcomes of a Cluster-Randomized Controlled Trial. *Journal of Physical Activity & Health*, *17*(10), 1009–1018. https://doi.org/10.1123/jpah.2019-0595
91. Patton, S. R., Monzon, A. D., Noser, A. E., & Clements, M. A. (2022). Physical Activity, Glycemic Variability, and Parental Hypoglycemia Fear in Preschoolers With Type 1 Diabetes. *Pediatric Exercise Science*, *34*(3), 135–140. https://doi.org/10.1123/pes.2021-0046
92. Crotti, M., Rudd, J. R., Roberts, S., Boddy, L. M., Fitton Davies, K., O’Callaghan, L., Utesch, T., & Foweather, L. (2021). Effect of Linear and Nonlinear Pedagogy Physical Education Interventions on Children’s Physical Activity: A Cluster Randomized Controlled Trial (SAMPLE-PE). *Children*, *8*(1), Article 1. https://doi.org/10.3390/children8010049
93. Henriques-Neto, D., B Júdice, P., Peralta, M., & B Sardinha, L. (2021). Fitness, physical activity, or sedentary patterns? Integrated analysis with obesity surrogates in a large youth sample. *American Journal of Human Biology: The Official Journal of the Human Biology Council*, *33*(5), e23522. https://doi.org/10.1002/ajhb.23522
94. Kobel, S., Dreyhaupt, J., Wartha, O., Kettner, S., Hoffmann, B., & Steinacker, J. M. (2020). Intervention Effects of the Health Promotion Programme “Join the Healthy Boat” on Objectively Assessed Sedentary Time in Primary School Children in Germany. *International Journal of Environmental Research and Public Health*, *17*(23), 9029. https://doi.org/10.3390/ijerph17239029
95. Yu, C.-L., Chueh, T.-Y., Hsieh, S.-S., Tsai, Y.-J., Hung, C.-L., Huang, C.-J., Wu, C.-T., & Hung, T.-M. (2019). Motor competence moderates relationship between moderate to vigorous physical activity and resting EEG in children with ADHD. *Mental Health and Physical Activity*, *17*, 100302. https://doi.org/10.1016/j.mhpa.2019.100302
96. Joschtel, B., Gomersall, S. R., Tweedy, S., Petsky, H., Chang, A. B., & Trost, S. G. (2019). Objectively measured physical activity and sedentary behaviour in children with bronchiectasis: A cross-sectional study. *BMC Pulmonary Medicine*, *19*(1), 7. https://doi.org/10.1186/s12890-018-0772-8
97. Kwon, S., O’Brien, M. K., Welch, S. B., & Honegger, K. (2022). Physical Activity among U.S. Preschool-Aged Children: Application of Machine Learning Physical Activity Classification to the 2012 National Health and Nutrition Examination Survey National Youth Fitness Survey. *Children (Basel, Switzerland)*, *9*(10), 1433. https://doi.org/10.3390/children9101433
98. Manyanga, T., Barnes, J. D., Chaput, J.-P., Katzmarzyk, P. T., Prista, A., & Tremblay, M. S. (2019). Prevalence and correlates of adherence to movement guidelines among urban and rural children in Mozambique: A cross-sectional study. *The International Journal of Behavioral Nutrition and Physical Activity*, *16*(1), 94. https://doi.org/10.1186/s12966-019-0861-y
99. Cremone, A., de Jong, D. M., Kurdziel, L. B. F., Desrochers, P., Sayer, A., LeBourgeois, M. K., Spencer, R. M. C., & McDermott, J. M. (2018). Sleep Tight, Act Right: Negative Affect, Sleep and Behavior Problems During Early Childhood. *Child Development*, *89*(2), e42–e59. <https://doi.org/10.1111/cdev.12717>
100. Higgins, S., Stoner, L., Black, K., Wong, J. E., Quigg, R., Meredith-Jones, K., & Skidmore, P. M. (2021). Social jetlag is associated with obesity-related outcomes in 9-11-year-old children, independent of other sleep characteristics. *Sleep Medicine*, *84*, 294–302. https://doi.org/10.1016/j.sleep.2021.06.014
101. Schroeder, K., Kubik, M. Y., Lee, J., Sirard, J. R., & Fulkerson, J. A. (2020). Self-Efficacy, Not Peer or Parent Support, Is Associated With More Physical Activity and Less Sedentary Time Among 8- to 12-Year-Old Youth With Elevated Body Mass Index. *Journal of Physical Activity & Health*, *17*(1), 74–79. https://doi.org/10.1123/jpah.2019-0108
102. Tsuda, E., Goodway, J. D., Famelia, R., & Brian, A. (2020). Relationship Between Fundamental Motor Skill Competence, Perceived Physical Competence and Free-Play Physical Activity in Children. *Research Quarterly for Exercise and Sport*, *91*(1), 55–63. https://doi.org/10.1080/02701367.2019.1646851
103. Wang, Y., He, G., Ma, K., Li, D., & Wang, C. (2022). Preschool Children’s Physical Activity and Community Environment: A Cross-Sectional Study of Two Cities in China. *International Journal of Environmental Research and Public Health*, *19*(22), 14797. https://doi.org/10.3390/ijerph192214797
104. Zask, A., Pattinson, M., Ashton, D., Ahmadi, M., Trost, S., Irvine, S., Stafford, L., Delbaere, K., & Adams, J. (2023). The effects of active classroom breaks on moderate to vigorous physical activity, behaviour and performance in a Northern NSW primary school: A quasi-experimental study. *Health Promotion Journal of Australia: Official Journal of Australian Association of Health Promotion Professionals*, *34*(4), 799–808. https://doi.org/10.1002/hpja.688
105. Wright, K. E., Furzer, B. J., Licari, M. K., Dimmock, J. A., & Jackson, B. (2020). The effect of parental logistic support on physical activity in children with, or at risk of, movement difficulties. *Journal of Science and Medicine in Sport*, *23*(4), 372–376. https://doi.org/10.1016/j.jsams.2019.10.005
106. Brazendale, K., Beets, M. W., Turner-McGrievy, G. M., Kaczynski, A. T., Pate, R. R., & Weaver, R. G. (2018). Children’s Obesogenic Behaviors During Summer Versus School: A Within-Person Comparison. *The Journal of School Health*, *88*(12), 886–892. https://doi.org/10.1111/josh.12699
107. Riso, E.-M., Kull, M., Mooses, K., & Jürimäe, J. (2018). Physical activity, sedentary time and sleep duration: Associations with body composition in 10–12-year-old Estonian schoolchildren. *BMC Public Health*, *18*(1), 496. https://doi.org/10.1186/s12889-018-5406-9
108. Yamakita, M., Ando, D., Akiyama, Y., Sato, M., Suzuki, K., & Yamagata, Z. (2019). Association of objectively measured physical activity and sedentary behavior with bone stiffness in peripubertal children. *Journal of Bone and Mineral Metabolism*, *37*(6), 1095–1103. https://doi.org/10.1007/s00774-019-01021-z
109. Leppänen, M. H., Migueles, J. H., Abdollahi, A. M., Engberg, E., Ortega, F. B., & Roos, E. (2022). Comparing estimates of physical activity in children across different cut-points and the associations with weight status. *Scandinavian Journal of Medicine & Science in Sports*, *32*(6), 971–983. https://doi.org/10.1111/sms.14147
110. Tan, S. Y. X., Chia, A., Tai, B. C., Natarajan, P., Goh, C. M. J. L., Shek, L. P., Saw, S. M., Chong, M. F.-F., & Müller-Riemenschneider, F. (2022). A Web-Based, Time-Use App To Assess Children’s Movement Behaviors: Validation Study of My E-Diary for Activities and Lifestyle (MEDAL). *JMIR Pediatrics and Parenting*, *5*(2), e33312. https://doi.org/10.2196/33312
111. McLellan, G., Arthur, R., Donnelly, S., & Buchan, D. S. (2020). Segmented sedentary time and physical activity patterns throughout the week from wrist-worn ActiGraph GT3X+ accelerometers among children 7-12 years old. *Journal of Sport and Health Science*, *9*(2), 179–188. https://doi.org/10.1016/j.jshs.2019.02.005
112. Winsor, A. A., Richards, C., Seri, S., Liew, A., & Bagshaw, A. P. (2023). The contribution of sleep and co-occurring neurodevelopmental conditions to quality of life in children with epilepsy. *Epilepsy Research*, *194*, 107188. https://doi.org/10.1016/j.eplepsyres.2023.107188
113. Lott, D. J., Taivassalo, T., Senesac, C. R., Willcocks, R. J., Harrington, A. M., Zilke, K., Cunkle, H., Powers, C., Finanger, E. L., Rooney, W. D., Tennekoon, G. I., & Vandenborne, K. (2021). Walking activity in a large cohort of boys with Duchenne muscular dystrophy. *Muscle & Nerve*, *63*(2), 192–198. https://doi.org/10.1002/mus.27119
114. Kariippanon, K. E., Chong, K. H., Janssen, X., Tomaz, S. A., Ribeiro, E. H., Munambah, N., ... & D OKELY, A. N. T. H. O. N. Y. (2022). Levels and correlates of objectively measured sedentary behavior in young children: SUNRISE study results from 19 countries. *Medicine and science in sports and exercise*, *54*(7), 1123.
115. Lu, C., Shen, T., Huang, G., & Corpeleijn, E. (2022). Environmental correlates of sedentary behaviors and physical activity in Chinese preschool children: A cross-sectional study. *Journal of Sport and Health Science*, *11*(5), 620–629. https://doi.org/10.1016/j.jshs.2020.02.010
116. Vyhlídal, T., Dygrýn, J., Pelclová, J., & Chmelík, F. (2022). Movement behaviours in paediatric cancer survivors during recovery and school weeks. *Frontiers in Oncology*, *12*. https://doi.org/10.3389/fonc.2022.971805
117. da Costa, B. G. G., Bruner, B., Scharoun Benson, S., Raymer, G., & Law, B. (2022). Canadian Children’s Physical Activity and Sedentary Behaviors During Time-Segments of the School Day. *American Journal of Health Education*, *53*(4), 197–206. https://doi.org/10.1080/19325037.2022.2071781
118. Robbins, L. B., Ling, J., Clevenger, K., Voskuil, V. R., Wasilevich, E., Kerver, J. M., Kaciroti, N., & Pfeiffer, K. A. (2020). A School- and Home-Based Intervention to Improve Adolescents’ Physical Activity and Healthy Eating: A Pilot Study. *The Journal of School Nursing: The Official Publication of the National Association of School Nurses*, *36*(2), 121–134. https://doi.org/10.1177/1059840518791290
119. Schwarzfischer, P., Gruszfeld, D., Socha, P., Luque, V., Closa-Monasterolo, R., Rousseaux, D., Moretti, M., Mariani, B., Verduci, E., Koletzko, B., & Grote, V. (2018). Longitudinal analysis of physical activity, sedentary behaviour and anthropometric measures from ages 6 to 11 years. *International Journal of Behavioral Nutrition and Physical Activity*, *15*(1), 126. https://doi.org/10.1186/s12966-018-0756-3
120. Lambrechtse, P., Ziesenitz, V. C., Atkinson, A., Bos, E. J., Welzel, T., Gilgen, Y., Gürtler, N., Heuscher, S., Cohen, A. F., & van den Anker, J. N. (2021). Monitoring the recovery time of children after tonsillectomy using commercial activity trackers. *European Journal of Pediatrics*, *180*(2), 527–533. https://doi.org/10.1007/s00431-020-03900-4
121. Sherry, A. P., Pearson, N., Ridgers, N. D., Barber, S. E., Bingham, D. D., Nagy, L. C., & Clemes, S. A. (2019). activPAL-measured sitting levels and patterns in 9-10 years old children from a UK city. *Journal of Public Health (Oxford, England)*, *41*(4), 757–764. https://doi.org/10.1093/pubmed/fdy181
122. Dumuid, D., Wake, M., Burgner, D., Tremblay, M. S., Okely, A. D., Edwards, B., Dwyer, T., & Olds, T. (2021). Balancing time use for children’s fitness and adiposity: Evidence to inform 24-hour guidelines for sleep, sedentary time and physical activity. *PloS One*, *16*(1), e0245501. https://doi.org/10.1371/journal.pone.0245501
123. Abel, E. A., Schwichtenberg, A. J., Brodhead, M. T., & Christ, S. L. (2018). Sleep and Challenging Behaviors in the Context of Intensive Behavioral Intervention for Children with Autism. *Journal of Autism and Developmental Disorders*, *48*(11), 3871–3884. https://doi.org/10.1007/s10803-018-3648-0
124. Kidokoro, T., Shimizu, Y., Edamoto, K., & Annear, M. (2019). Classroom Standing Desks and Time-Series Variation in Sedentary Behavior and Physical Activity among Primary School Children. *International Journal of Environmental Research and Public Health*, *16*(11), 1892. https://doi.org/10.3390/ijerph16111892
125. Kallio, J., Hakonen, H., Syväoja, H., Kulmala, J., Kankaanpää, A., Ekelund, U., & Tammelin, T. (2020). Changes in physical activity and sedentary time during adolescence: Gender differences during weekdays and weekend days. *Scandinavian Journal of Medicine & Science in Sports*, *30*(7), 1265–1275. https://doi.org/10.1111/sms.13668
126. Ranum, B. M., Wichstrøm, L., Pallesen, S., Falch-Madsen, J., Halse, M., & Steinsbekk, S. (2019). Association Between Objectively Measured Sleep Duration and Symptoms of Psychiatric Disorders in Middle Childhood. *JAMA Network Open*, *2*(12), e1918281. https://doi.org/10.1001/jamanetworkopen.2019.18281
127. Swartz, A. M., Tokarek, N. R., Lisdahl, K., Maeda, H., Strath, S. J., & Cho, C. C. (2019). Do Stand-Biased Desks in the Classroom Change School-Time Activity and Sedentary Behavior? *International Journal of Environmental Research and Public Health*, *16*(6), 933. https://doi.org/10.3390/ijerph16060933
128. Santiago-Rodríguez, M. E., Ramer, J. D., Marquez, D. X., Frazier, S. L., Davis, C. L., & Bustamante, E. E. (2022). Device-Based Movement Behaviors, Executive Function, and Academic Skills among African American Children with ADHD and Disruptive Behavior Disorders. *International Journal of Environmental Research and Public Health*, *19*(7), 4032. https://doi.org/10.3390/ijerph19074032
129. Sánchez-Oliva, D., Esteban-Cornejo, I., Padilla-Moledo, C., Pérez-Bey, A., Veiga, Ó. L., Cabanas-Sánchez, V., & Castro-Piñero, J. (2020). Associations between physical activity and sedentary time profiles transitions and changes in well-being in youth: The UP&DOWN longitudinal study. *Psychology of Sport and Exercise*, *47*, 101558. https://doi.org/10.1016/j.psychsport.2019.101558
130. Salmon, J., Arundell, L., Cerin, E., Ridgers, N. D., Hesketh, K. D., Daly, R. M., Dunstan, D., Brown, H., Gatta, J. D., Gatta, P. D., Chinapaw, M. J. M., Shepphard, L., Moodie, M., Hume, C., Brown, V., Ball, K., & Crawford, D. (2023). Transform-Us! cluster RCT: 18-month and 30-month effects on children’s physical activity, sedentary time and cardiometabolic risk markers. *British Journal of Sports Medicine*, *57*(5), 311–319. https://doi.org/10.1136/bjsports-2022-105825
131. Gerber, C. N., Carcreff, L., Paraschiv-Ionescu, A., Armand, S., & Newman, C. J. (2021). Reliability of single-day walking performance and physical activity measures using inertial sensors in children with cerebral palsy. *Annals of Physical and Rehabilitation Medicine*, *64*(3), 101250. https://doi.org/10.1016/j.rehab.2019.02.003
132. Mitchell, F., Wilkie, L., Robertson, K., Reilly, J. J., & Kirk, A. (2018). Feasibility and pilot study of an intervention to support active lifestyles in youth with type 1 diabetes: The ActivPals study. *Pediatric Diabetes*, *19*(3), 443–449. https://doi.org/10.1111/pedi.12615
133. Bloemen, M. A. T., van den Berg-Emons, R. J. G., Tuijt, M., Nooijen, C. F. J., Takken, T., Backx, F. J. G., Vos, M., & de Groot, J. F. (2019). Physical activity in wheelchair-using youth with spina bifida: An observational study. *Journal of NeuroEngineering and Rehabilitation*, *16*(1), 9. https://doi.org/10.1186/s12984-018-0464-x
134. Loram, G., Silk, T., Ling, M., & Sciberras, E. (2024). Examining the associations between attention-deficit/hyperactivity disorder, sleep problems, and other mental health conditions in adolescents. *Journal of Sleep Research*, *33*(2), e13830. https://doi.org/10.1111/jsr.13830
135. Price, L., Wyatt, K., Lloyd, J., Abraham, C., Creanor, S., Dean, S., & Hillsdon, M. (2018). Children’s Compliance With Wrist-Worn Accelerometry Within a Cluster-Randomized Controlled Trial: Findings From the Healthy Lifestyles Programme. *Pediatric Exercise Science*, *30*(2), 281–287. https://doi.org/10.1123/pes.2017-0179
136. Pate, R. R., Dowda, M., Dishman, R. K., Colabianchi, N., Saunders, R. P., & McIver, K. L. (2019). Change in Children’s Physical Activity: Predictors in the Transition From Elementary to Middle School. *American Journal of Preventive Medicine*, *56*(3), e65–e73. https://doi.org/10.1016/j.amepre.2018.10.012
137. Parry, S., Ir de Oliveira, B., McVeigh, J. A., Ee, J., Jacques, A., & Straker, L. (2019). Standing Desks in a Grade 4 Classroom over the Full School Year. *International Journal of Environmental Research and Public Health*, *16*(19), 3590. https://doi.org/10.3390/ijerph16193590
138. Nigg, C., Niessner, C., Nigg, C. R., Oriwol, D., Schmidt, S. C. E., & Woll, A. (2021). Relating outdoor play to sedentary behavior and physical activity in youth—Results from a cohort study. *BMC Public Health*, *21*(1), 1716. https://doi.org/10.1186/s12889-021-11754-0
139. Naya, C. H., Zink, J., Huh, J., Dunton, G. F., & Belcher, B. R. (2021). Examining the same-day relationship between morning cortisol after awakening, perceived stress in the morning, and physical activity in youth. *Stress (Amsterdam, Netherlands)*, *24*(3), 338–347. https://doi.org/10.1080/10253890.2020.1804852
140. Mora-Gonzalez, J., Esteban-Cornejo, I., Cadenas-Sanchez, C., Migueles, J. H., Molina-Garcia, P., Rodriguez-Ayllon, M., Henriksson, P., Pontifex, M. B., Catena, A., & Ortega, F. B. (2019). Physical Fitness, Physical Activity, and the Executive Function in Children with Overweight and Obesity. *The Journal of Pediatrics*, *208*, 50-56.e1. https://doi.org/10.1016/j.jpeds.2018.12.028
141. Molina-García, J., Menescardi, C., Estevan, I., & Queralt, A. (2021). Associations between Park and Playground Availability and Proximity and Children’s Physical Activity and Body Mass Index: The BEACH Study. *International Journal of Environmental Research and Public Health*, *19*(1), 250. https://doi.org/10.3390/ijerph19010250
142. Miadich, S. A., Doane, L. D., Davis, M. C., & Lemery-Chalfant, K. (2019). Early Parental Positive Personality and Stress: Longitudinal Associations with Children’s Sleep. *British Journal of Health Psychology*, *24*(3), 629–650. https://doi.org/10.1111/bjhp.12372
143. Jakubec, L., Gába, A., Dygrýn, J., Rubín, L., Šimůnek, A., & Sigmund, E. (2020). Is adherence to the 24-hour movement guidelines associated with a reduced risk of adiposity among children and adolescents? *BMC Public Health*, *20*, 1119. https://doi.org/10.1186/s12889-020-09213-3
144. Sturm, D. J., Bachner, J., Renninger, D., Haug, S., & Demetriou, Y. (2021). A cluster randomized trial to evaluate need-supportive teaching in physical education on physical activity of sixth-grade girls: A mixed method study. *Psychology of Sport and Exercise*, *54*, 101902. https://doi.org/10.1016/j.psychsport.2021.101902
145. Sinisterra, M., Hamburger, S., Tully, C., Hamburger, E., Jaser, S., & Streisand, R. (2020). Young Children with Type 1 Diabetes: Sleep, Health-Related Quality of Life, and Continuous Glucose Monitor Use. *Diabetes Technology & Therapeutics*, *22*(8), 639–642. https://doi.org/10.1089/dia.2019.0437
146. Silva, D. R., Minderico, C. S., Pinto, F., Collings, P. J., Cyrino, E. S., & Sardinha, L. B. (2018). Impact of a classroom standing desk intervention on daily objectively measured sedentary behavior and physical activity in youth. *Journal of Science and Medicine in Sport*, *21*(9), 919–924. https://doi.org/10.1016/j.jsams.2018.01.007
147. Bachner, J., Sturm, D. J., & Demetriou, Y. (2020). Accelerometer-Measured Physical Activity and Sedentary Behavior Levels and Patterns in Female Sixth Graders: The CReActivity Project. *International Journal of Environmental Research and Public Health*, *18*(1), 32. https://doi.org/10.3390/ijerph18010032
148. Nyberg, G., Kjellenberg, K., Fröberg, A., & Lindroos, A. K. (2020). A national survey showed low levels of physical activity in a representative sample of Swedish adolescents. *Acta Paediatrica (Oslo, Norway: 1992)*, *109*(11), 2342–2353. https://doi.org/10.1111/apa.15251
149. Franceschi, R., Scotton, C., Leonardi, L., Cauvin, V., Maines, E., Angriman, M., Pertile, R., Valent, F., Soffiati, M., & Faraguna, U. (2022). Impact of intermittently scanned continuous glucose monitoring with alarms on sleep and metabolic outcomes in children and adolescents with type 1 diabetes. *Acta Diabetologica*, *59*(7), 911–919. https://doi.org/10.1007/s00592-022-01882-3
150. Cabanas-Sánchez, V., Martínez-Gómez, D., Izquierdo-Gómez, R., Segura-Jiménez, V., Castro-Piñero, J., & Veiga, O. L. (2018). Association between Clustering of Lifestyle Behaviors and Health-Related Physical Fitness in Youth: The UP&DOWN Study. *The Journal of Pediatrics*, *199*, 41-48.e1. https://doi.org/10.1016/j.jpeds.2018.03.075
151. Armstrong, B., Beets, M. W., Starrett, A., Brazendale, K., Turner-McGrievy, G., Saelens, B. E., Pate, R. R., Youngstedt, S. D., Maydeu-Olivares, A., & Weaver, R. G. (2021). Dynamics of sleep, sedentary behavior, and moderate-to-vigorous physical activity on school versus nonschool days. *Sleep*, *44*(2), zsaa174. https://doi.org/10.1093/sleep/zsaa174
152. Bringolf-Isler, B., de Hoogh, K., Schindler, C., Kayser, B., Suggs, L. S., Dössegger, A., Probst-Hensch, N., & SOPHYA Study Group. (2018). Sedentary Behaviour in Swiss Children and Adolescents: Disentangling Associations with the Perceived and Objectively Measured Environment. *International Journal of Environmental Research and Public Health*, *15*(5), 918. https://doi.org/10.3390/ijerph15050918
153. Sprengeler, O., Pohlabeln, H., Bammann, K., Buck, C., Lauria, F., Verbestel, V., Eiben, G., Konstabel, K., Molnár, D., Moreno, L. A., Pitsiladis, Y., Page, A., Reisch, L., Tornaritis, M., & Ahrens, W. (2021). Trajectories of objectively measured physical activity and childhood overweight: Longitudinal analysis of the IDEFICS/I.Family cohort. *International Journal of Behavioral Nutrition and Physical Activity*, *18*(1), 103. https://doi.org/10.1186/s12966-021-01171-2
154. De Meester, A., Stodden, D., Goodway, J., True, L., Brian, A., Ferkel, R., & Haerens, L. (2018). Identifying a motor proficiency barrier for meeting physical activity guidelines in children. *Journal of Science and Medicine in Sport*, *21*(1), 58–62. https://doi.org/10.1016/j.jsams.2017.05.007
155. Clemes, S. A., Bingham, D. D., Pearson, N., Chen, Y.-L., Edwardson, C. L., McEachan, R. R. C., Tolfrey, K., Cale, L., Richardson, G., Fray, M., Altunkaya, J., Bandelow, S., Jaicim, N. B., Salmon, J., Dunstan, D. W., & Barber, S. E. (2020). Stand Out in Class: Restructuring the classroom environment to reduce sitting time – findings from a pilot cluster randomised controlled trial. *International Journal of Behavioral Nutrition and Physical Activity*, *17*(1), 55. https://doi.org/10.1186/s12966-020-00958-z
156. Innerd, A. L., Azevedo, L. B., & Batterham, A. M. (2019). The effect of a curriculum-based physical activity intervention on accelerometer-assessed physical activity in schoolchildren: A non-randomised mixed methods controlled before-and-after study. *PloS One*, *14*(12), e0225997. https://doi.org/10.1371/journal.pone.0225997
157. Shoesmith, A., Hall, A., Hope, K., Sutherland, R., Hodder, R. K., Trost, S. G., Lecathelinais, C., Lane, C., McCarthy, N., & Nathan, N. (2020). Associations between in-school-hours physical activity and child health-related quality of life: A cross-sectional study in a sample of Australian primary school children. *Preventive Medicine Reports*, *20*, 101179. https://doi.org/10.1016/j.pmedr.2020.101179
158. Buchan, D. S., & Maylor, B. D. (2023). Comparison of physical activity metrics from two research-grade accelerometers worn on the non-dominant wrist and thigh in children. *Journal of Sports Sciences*, *41*(1), 80–88. https://doi.org/10.1080/02640414.2023.2197726
159. Strugnell, C., Crooks, N., Gaskin, C. J., Becker, D., Orellana, L., Bolton, K. A., Fraser, P., Brown, A. D., Le, H., Bell, C., & Allender, S. (2023). Four-Year Accelerometry Outcomes from a Cluster Randomized Whole of Systems Trial of Prevention Strategies for Childhood Obesity. *Childhood Obesity (Print)*, *19*(5), 332–340. https://doi.org/10.1089/chi.2022.0076
160. Seljebotn, P. H., Skage, I., Riskedal, A., Olsen, M., Kvalø, S. E., & Dyrstad, S. M. (2019). Physically active academic lessons and effect on physical activity and aerobic fitness. The Active School study: A cluster randomized controlled trial. *Preventive Medicine Reports*, *13*, 183–188. https://doi.org/10.1016/j.pmedr.2018.12.009
161. Sacheck, J. M., Wright, C. M., Amin, S. A., Anzman-Frasca, S., Chomitz, V. M., Chui, K. K., Duquesnay, P. J., Nelson, M. E., & Economos, C. D. (2021). The Fueling Learning Through Exercise Study Cluster RCT: Impact on Children’s Moderate-to-Vigorous Physical Activity. *American Journal of Preventive Medicine*, *60*(6), e239–e249. https://doi.org/10.1016/j.amepre.2021.01.002
162. Trickett, J., Oliver, C., Heald, M., Denyer, H., Surtees, A., Clarkson, E., Gringras, P., & Richards, C. (2019). Multi-Method Assessment of Sleep in Children With Angelman Syndrome: A Case-Controlled Study. *Frontiers in Psychiatry*, *10*, 874. https://doi.org/10.3389/fpsyt.2019.00874
163. Reedman, S. E., Boyd, R. N., Trost, S. G., Elliott, C., & Sakzewski, L. (2019). Efficacy of Participation-Focused Therapy on Performance of Physical Activity Participation Goals and Habitual Physical Activity in Children With Cerebral Palsy: A Randomized Controlled Trial. *Archives of Physical Medicine and Rehabilitation*, *100*(4), 676–686. https://doi.org/10.1016/j.apmr.2018.11.012
164. Braaksma, P., Stuive, I., Jelsma, D., Van der Sluis, C. K., Dekker, R., & Schoemaker, M. M. (2022). Effectiveness and feasibility of We12BFit!: Improving physical fitness and lifestyle physical activity in children with developmental coordination disorder in a paediatric rehabilitation setting-a small sample field study. *BMJ Open*, *12*(4), e044626. https://doi.org/10.1136/bmjopen-2020-044626
165. Philbrook, L. E., Aguilar, K., Bohan, A. R., Daza, K. M., & Harris, S. L. (2022). Bedtime parenting practices and sensitivity are associated with young children’s sleep. *Journal of Family Psychology: JFP: Journal of the Division of Family Psychology of the American Psychological Association (Division 43)*, *36*(8), 1473–1479. https://doi.org/10.1037/fam0001027
166. Thorpe, N., Harniess, P., Main, E., Hubert, N., Rand, S., Stephensen, D., Liesner, R., & Bladen, M. (2021). Feasibility, safety and acceptability of select outcome measures in a physiotherapy study protocol for boys with haemophilia. *Pilot and Feasibility Studies*, *7*(1), 105. https://doi.org/10.1186/s40814-021-00831-1
167. Traube, C., Rosenberg, L., Thau, F., Gerber, L. M., Mauer, E. A., Seghini, T., Gulati, N., Taylor, D., Silver, G., & Kudchadkar, S. R. (2020). Sleep in Hospitalized Children With Cancer: A Cross-Sectional Study. *Hospital Pediatrics*, *10*(11), 969–976. https://doi.org/10.1542/hpeds.2020-0101
168. Crowe, R. K., Probst, Y. C., Stanley, R. M., Ryan, S. T., Weaver, R. G., Beets, M. W., Norman, J. A., Furber, S. E., Vuong, C., Hammersley, M. L., Wardle, K., Franco, L., Davies, M., Innes-Hughes, C., & Okely, A. D. (2021). Physical activity in out of school hours care: An observational study. *International Journal of Behavioral Nutrition and Physical Activity*, *18*(1), 127. https://doi.org/10.1186/s12966-021-01197-6
169. Woods, A. M., McLoughlin, G. M., Kern, B. D., & Graber, K. C. (2018). What’s Physical Activity Got to Do With It? Social Trends in Less Active Students at Recess. *The Journal of School Health*, *88*(7), 500–507. https://doi.org/10.1111/josh.12637
170. Noonan, R. J., Christian, D., Boddy, L. M., Saint-Maurice, P. F., Welk, G. J., Hibbing, P. R., & Fairclough, S. J. (2019). Accelerometer and self-reported measures of sedentary behaviour and associations with adiposity in UK youth. *Journal of Sports Sciences*, *37*(16), 1919–1925. https://doi.org/10.1080/02640414.2019.1605649
171. Ovans, J. A., Hooke, M. C., Bendel, A. E., & Tanner, L. R. (2018). Physical Therapist Coaching to Improve Physical Activity in Children With Brain Tumors: A Pilot Study. *Pediatric Physical Therapy: The Official Publication of the Section on Pediatrics of the American Physical Therapy Association*, *30*(4), 310–317. https://doi.org/10.1097/PEP.0000000000000531
172. Quirk, H., Heller, B., & Wright, N. (2020). Feasibility and Acceptability of Physical Activity Monitoring as an Educational Tool in Management of Pediatric Type 1 Diabetes. *Canadian Journal of Diabetes*, *44*(8), 688–696. https://doi.org/10.1016/j.jcjd.2020.06.013
173. Roth, N., Lev-Wiesel, R., & Shochat, T. (2019). “How do you sleep?” sleep in self-figure drawings of young adolescents in residential care facilities—An exploratory study. *Sleep Medicine*, *60*, 116–122. https://doi.org/10.1016/j.sleep.2019.01.028
174. Esbensen, A. J., Hoffman, E. K., Beebe, D. W., Byars, K. C., & Epstein, J. (2018). Links between sleep and daytime behaviour problems in children with Down syndrome. *Journal of Intellectual Disability Research: JIDR*, *62*(2), 115–125. https://doi.org/10.1111/jir.12463
175. Belcher, B. R., Wolff-Hughes, D. L., Dooley, E. E., Staudenmayer, J., Berrigan, D., Eberhardt, M. S., & Troiano, R. P. (2021). US Population-referenced Percentiles for Wrist-Worn Accelerometer-derived Activity. *Medicine and Science in Sports and Exercise*, *53*(11), 2455–2464. https://doi.org/10.1249/MSS.0000000000002726
176. Bartholomew, J. B., Jowers, E. M., Roberts, G., Fall, A.-M., Errisuriz, V. L., & Vaughn, S. (2018). Active Learning Increases Children’s Physical Activity across Demographic Subgroups. *Translational Journal of the American College of Sports Medicine*, *3*(1), 1–9. https://doi.org/10.1249/TJX.0000000000000051
177. Burford, K., Gillespie, K., Jowers, E. M., & Bartholomew, J. B. (2022). Children’s Enjoyment, Perceived Competency, and Vigorous Physical Activity During High-Intensity Interval Training in Physical Education. *Research Quarterly for Exercise and Sport*, *93*(4), 835–844. <https://doi.org/10.1080/02701367.2021.1925207>
178. Giddens, N. T., Juneau, P., Manza, P., Wiers, C. E., & Volkow, N. D. (2022). Disparities in sleep duration among American children: Effects of race and ethnicity, income, age, and sex. *Proceedings of the National Academy of Sciences of the United States of America*, *119*(30), e2120009119. https://doi.org/10.1073/pnas.2120009119
179. Grant, V. M., Tomayko, E. J., & Kingfisher, R. D. (2020). Sleep and Physical Activity Patterns in Urban American Indian Children. *American Journal of Health Behavior*, *44*(1), 67–75. https://doi.org/10.5993/AJHB.44.1.7
180. Hartikainen, J., Haapala, E. A., Poikkeus, A.-M., Sääkslahti, A., Laukkanen, A., Gao, Y., & Finni, T. (2023). Classroom-based physical activity and teachers’ instructions on students’ movement in conventional classrooms and open learning spaces. *Learning Environments Research*, *26*(1), 177–198. https://doi.org/10.1007/s10984-022-09411-3
181. Lecarie, E. K., Doane, L. D., Clifford, S., & Lemery-Chalfant, K. (2022). The onset of pubertal development and actigraphy-assessed sleep during middle childhood: Racial, gender, and genetic effects. *Sleep Health*, *8*(2), 208–215. https://doi.org/10.1016/j.sleh.2021.12.006
182. Breitenstein, R. S., Doane, L. D., & Lemery-Chalfant, K. (2021). Children’s objective sleep assessed with wrist-based accelerometers: Strong heritability of objective quantity and quality unique from parent-reported sleep. *Sleep*, *44*(1), zsaa142. https://doi.org/10.1093/sleep/zsaa142
183. Finkelstein, E. A., Lim, R. S. M., Ward, D. S., & Evenson, K. R. (2020). Leveraging family dynamics to increase the effectiveness of incentives for physical activity: The FIT-FAM randomized controlled trial. *International Journal of Behavioral Nutrition and Physical Activity*, *17*(1), 113. https://doi.org/10.1186/s12966-020-01018-2
184. Merbler, A. M., Byiers, B. J., Garcia, J. J., Feyma, T. J., & Symons, F. J. (2018). The feasibility of using actigraphy to characterize sleep in Rett syndrome. *Journal of Neurodevelopmental Disorders*, *10*(1), 8. https://doi.org/10.1186/s11689-018-9227-z
185. Bergqvist-Norén, L., Hagman, E., Xiu, L., Marcus, C., & Hagströmer, M. (2022). Physical activity in early childhood: A five-year longitudinal analysis of patterns and correlates. *International Journal of Behavioral Nutrition and Physical Activity*, *19*(1), 47. https://doi.org/10.1186/s12966-022-01289-x
186. Rast, F. M., Herren, S., & Labruyère, R. (2022). Acceptability of wearable inertial sensors, completeness of data, and day-to-day variability of everyday life motor activities in children and adolescents with neuromotor impairments. *Frontiers in Rehabilitation Sciences*, *3*. https://doi.org/10.3389/fresc.2022.923328
187. Løndal, K., Haugen, A. L. H., Lund, S., & Riiser, K. (2020). Physical activity of first graders in Norwegian after-school programs: A relevant contribution to the development of motor competencies and learning of movements? Investigated utilizing a mixed methods approach. *PLoS ONE*, *15*(4), e0232486. https://doi.org/10.1371/journal.pone.0232486
188. Lokhandwala, S., Holmes, J. F., Mason, G. M., St Laurent, C. W., Delvey, C., Hanron, O., Andre, C., Rodheim, K., Kaur, S., & Spencer, R. M. C. (2021). Sleep and Coping in Early Childhood During the COVID-19 Pandemic. *Frontiers in Pediatrics*, *9*, 716608. https://doi.org/10.3389/fped.2021.716608
189. Lindhiem, O., Goel, M., Shaaban, S., Mak, K. J., Chikersal, P., Feldman, J., & Harris, J. L. (2022). Objective Measurement of Hyperactivity Using Mobile Sensing and Machine Learning: Pilot Study. *JMIR Formative Research*, *6*(4), e35803. https://doi.org/10.2196/35803
190. Kahn, D., Iturriaga, C., Bertran, K., Fernandez, I., Perez-Mateluna, G., Borzutzky, A., & Brockmann, P. E. (2020). Sleep quality in children with atopic dermatitis during flares and after treatment. *Sleep Science*, *13*(2), 172–175. https://doi.org/10.5935/1984-0063.20190139
191. Johnstone, A., Hughes, A. R., Bonnar, L., Booth, J. N., & Reilly, J. J. (2019). An active play intervention to improve physical activity and fundamental movement skills in children of low socio-economic status: Feasibility cluster randomised controlled trial. *Pilot and Feasibility Studies*, *5*(1), 45. https://doi.org/10.1186/s40814-019-0427-4
192. Huertas-Delgado, F. J., Segura-Jiménez, V., Ávila-García, M., Cardon, G., & Tercedor, P. (2021). Physical activity levels during physical education in Spanish children. *Health Education Journal*. https://doi.org/10.1177/0017896920988743
193. Heikkilä, A.-R., Lapinleimu, H., Virtanen, I., Rönnlund, H., Raaska, H., & Elovainio, M. (2022). Changes in objectively measured sleep among internationally adopted children in 1-year follow-up during the first years in new families. *Frontiers in Pediatrics*, *10*, 948010. https://doi.org/10.3389/fped.2022.948010
194. Salway, R., Emm-Collison, L., Sebire, S. J., Thompson, J. L., Lawlor, D. A., & Jago, R. (2019). The association of school-related active travel and active after-school clubs with children’s physical activity: A cross-sectional study in 11-year-old UK children. *International Journal of Behavioral Nutrition and Physical Activity*, *16*(1), 72. https://doi.org/10.1186/s12966-019-0832-3
195. Barnett, L. M., Telford, R. M., Strugnell, C., Rudd, J., Olive, L. S., & Telford, R. D. (2019). Impact of cultural background on fundamental movement skill and its correlates. *Journal of Sports Sciences*, *37*(5), 492–499. https://doi.org/10.1080/02640414.2018.1508399
196. Bartelink, N. H. M., van Assema, P., Kremers, S. P. J., Savelberg, H. H. C. M., Oosterhoff, M., Willeboordse, M., van Schayck, O. C. P., Winkens, B., & Jansen, M. W. J. (2019). One- and Two-Year Effects of the Healthy Primary School of the Future on Children’s Dietary and Physical Activity Behaviours: A Quasi-Experimental Study. *Nutrients*, *11*(3), 689. https://doi.org/10.3390/nu11030689
197. Borghese, M., Lin, Y., Chaput, J., & Janssen, I. (2018). Estimating sleep efficiency in 10- to- 13-year-olds using a waist-worn accelerometer. *Sleep Health*, *4*(1), 110–115. https://doi.org/10.1016/j.sleh.2017.09.006
198. Chevalier, L. L. (2020). *Evaluation of a treatment of sleep-related problems in children with anxiety using a multiple baseline design*. Boston University.
199. Clevenger, K. A., Belcher, B. R., & Berrigan, D. (2022). Associations between Amount of Recess, Physical Activity, and Cardiometabolic Traits in U.S. Children. *Translational Journal of the American College of Sports Medicine*, *7*(3), e000202. https://doi.org/10.1249/tjx.0000000000000202
200. Kruizinga, M. D., Heide, N. van der, Moll, A., Zhuparris, A., Yavuz, Y., Kam, M. L. de, Stuurman, F. E., Cohen, A. F., & Driessen, G. J. A. (2021). Towards remote monitoring in pediatric care and clinical trials-Tolerability, repeatability and reference values of candidate digital endpoints derived from physical activity, heart rate and sleep in healthy children. *PloS One*, *16*(1), e0244877. https://doi.org/10.1371/journal.pone.0244877
201. Kruizinga, M. D., Essers, E., Stuurman, F. E., Yavuz, Y., de Kam, M. L., Zhuparris, A., Janssens, H. M., Groothuis, I., Sprij, A. J., Nuijsink, M., Cohen, A. F., & Driessen, G. J. A. (2022). Clinical validation of digital biomarkers for paediatric patients with asthma and cystic fibrosis: Potential for clinical trials and clinical care. *The European Respiratory Journal*, *59*(6), 2100208. https://doi.org/10.1183/13993003.00208-2021
202. Jaser, S. S., Bergner, E. M., Hamburger, E. R., Bhatia, S., Lyttle, M., Bell, G. E., Slaughter, J. C., Malow, B. A., & Simmons, J. H. (2021). Pilot Trial of a Sleep-Promoting Intervention for Children With Type 1 Diabetes. *Journal of Pediatric Psychology*, *46*(3), 304–313. <https://doi.org/10.1093/jpepsy/jsaa105>
203. Götte, M., Kesting, S. V., Gerss, J., Rosenbaum, D., & Boos, J. (2018). Feasibility and effects of a home-based intervention using activity trackers on achievement of individual goals, quality of life and motor performance in patients with paediatric cancer. *BMJ Open Sport & Exercise Medicine*, *4*(1), e000322. https://doi.org/10.1136/bmjsem-2017-000322
204. Ha, L., Wakefield, C. E., Mizrahi, D., Diaz, C., Cohn, R. J., Signorelli, C., Yacef, K., & Simar, D. (2022). A Digital Educational Intervention With Wearable Activity Trackers to Support Health Behaviors Among Childhood Cancer Survivors: Pilot Feasibility and Acceptability Study. *JMIR Cancer*, *8*(3), e38367. https://doi.org/10.2196/38367
205. Leppänen, M. H., Migueles, J. H., Cadenas-Sanchez, C., Henriksson, P., Mora-Gonzalez, J., Henriksson, H., Labayen, I., Löf, M., Esteban-Cornejo, I., & Ortega, F. B. (2020). Hip and wrist accelerometers showed consistent associations with fitness and fatness in children aged 8-12 years. *Acta Paediatrica (Oslo, Norway: 1992)*, *109*(5), 995–1003. https://doi.org/10.1111/apa.15043
206. Rhodes, R. E., Blanchard, C. M., Quinlan, A., Naylor, P.-J., & Warburton, D. E. R. (2019). Family Physical Activity Planning and Child Physical Activity Outcomes: A Randomized Trial. *American Journal of Preventive Medicine*, *57*(2), 135–144. https://doi.org/10.1016/j.amepre.2019.03.007
207. Rodríguez-Rodríguez, F., Cristi-Montero, C., & Castro-Piñero, J. (2020). Physical Activity Levels of Chilean Children in a National School Intervention Programme. A Quasi-Experimental Study. *International Journal of Environmental Research and Public Health*, *17*(12), 4529. https://doi.org/10.3390/ijerph17124529
208. Willeboordse, M., Bartelink, N. H. M., van Assema, P., Kremers, S. P. J., Savelberg, H. H. C. M., Hahnraths, M. T. H., Vonk, L., Oosterhoff, M., van Schayck, C. P., Winkens, B., & Jansen, M. W. J. (2022). Battling the obesity epidemic with a school-based intervention: Long-term effects of a quasi-experimental study. *PloS One*, *17*(9), e0272291. <https://doi.org/10.1371/journal.pone.0272291>
209. Jago, R., Salway, R., Emm-Collison, L., Sebire, S. J., Thompson, J. L., & Lawlor, D. A. (2020). Association of BMI category with change in children’s physical activity between ages 6 and 11 years: A longitudinal study. *International Journal of Obesity (2005)*, *44*(1), 104–113. https://doi.org/10.1038/s41366-019-0459-0
210. Dishman, R. K., McIver, K. L., Dowda, M., Saunders, R. P., & Pate, R. R. (2019). Self-Efficacy, Beliefs and Goals: Moderation of Declining Physical Activity during Adolescence. *Health Psychology : Official Journal of the Division of Health Psychology, American Psychological Association*, *38*(6), 483–493. https://doi.org/10.1037/hea0000734
211. Dunton, G. F., Wang, W.-L., Intille, S. S., Dzubur, E., Ponnada, A., & Hedeker, D. (2022). How acute affect dynamics impact longitudinal changes in physical activity among children. *Journal of Behavioral Medicine*, *45*(3), 451–460. https://doi.org/10.1007/s10865-022-00282-w
212. Caillaud, C., Ledger, S., Diaz, C., Clerc, G., Galy, O., & Yacef, K. (2022). iEngage: A digital health education program designed to enhance physical activity in young adolescents. *PLOS ONE*, *17*(10), e0274644. https://doi.org/10.1371/journal.pone.0274644
213. Verloigne, M., Ridgers, N. D., De Bourdeaudhuij, I., & Cardon, G. (2018). Effect and process evaluation of implementing standing desks in primary and secondary schools in Belgium: A cluster-randomised controlled trial. *International Journal of Behavioral Nutrition and Physical Activity*, *15*(1), 94. https://doi.org/10.1186/s12966-018-0726-9
214. Dalene, K. E., Anderssen, S. A., Andersen, L. B., Steene-Johannessen, J., Ekelund, U., Hansen, B. H., & Kolle, E. (2018). Cross-sectional and prospective associations between sleep, screen time, active school travel, sports/exercise participation and physical activity in children and adolescents. *BMC Public Health*, *18*(1), 705. https://doi.org/10.1186/s12889-018-5610-7
215. Pedersen, J., Rasmussen, M. G. B., Sørensen, S. O., Mortensen, S. R., Olesen, L. G., Brønd, J. C., Brage, S., Kristensen, P. L., & Grøntved, A. (2022). Effects of Limiting Recreational Screen Media Use on Physical Activity and Sleep in Families With Children: A Cluster Randomized Clinical Trial. *JAMA Pediatrics*, *176*(8), 741–749. https://doi.org/10.1001/jamapediatrics.2022.1519
216. Padmapriya, N., Chen, B., Goh, C. M. J. L., Shek, L. P. C., Chong, Y. S., Tan, K. H., Chan, S.-Y., Yap, F., Godfrey, K. M., Lee, Y. S., Eriksson, J. G., Bernard, J. Y., & Müller-Riemenschneider, F. (2021). 24-hour movement behaviour profiles and their transition in children aged 5.5 and 8 years – findings from a prospective cohort study. *International Journal of Behavioral Nutrition and Physical Activity*, *18*(1), 145. https://doi.org/10.1186/s12966-021-01210-y
217. Verswijveren, S. J. J. M., Ridgers, N. D., Martín-Fernández, J. A., Chastin, S., Cerin, E., Chinapaw, M. J. M., Arundell, L., Dunstan, D. W., Hume, C., Brown, H., Della Gatta, J., & Salmon, J. (2022). Intervention effects on children’s movement behaviour accumulation as a result of the Transform-Us! School- and home-based cluster randomised controlled trial. *International Journal of Behavioral Nutrition and Physical Activity*, *19*(1), 76. https://doi.org/10.1186/s12966-022-01314-z
218. Gantelius, S., Vikerfors, S., Edqvist, J. J., von Walden, F., Hagströmer, M., & Pontén, E. (2023). Time-matched accelerometers on limbs and waist in children with CP give new insights into real-life activities after botulinum toxin treatment: A proof of concept study. *Journal of Pediatric Rehabilitation Medicine*, *16*(1), 125–137. https://doi.org/10.3233/PRM-210112
219. Fishbein, A. B., Mueller, K., Kruse, L., Boor, P., Sheldon, S., Zee, P., & Paller, A. S. (2018). Sleep disturbance in children with moderate/severe atopic dermatitis: A case-control study. *Journal of the American Academy of Dermatology*, *78*(2), 336–341. https://doi.org/10.1016/j.jaad.2017.08.043
220. Goldschmidt, A. B., Evans, E. W., Saletin, J. M., O’Sullivan, K., Koren, D., Engel, S. G., & Haedt-Matt, A. (2020). Naturalistic, multimethod exploratory study of sleep duration and quality as predictors of dysregulated eating in youth with overweight and obesity. *Appetite*, *146*, 104521. https://doi.org/10.1016/j.appet.2019.104521
221. Harbottle, V., Bennett, J., Duong, C., Foster, H., & McErlane, F. (2018). 303 Feasibility of wearable technologies in children and young people with juvenile idiopathic arthritis. *Rheumatology*, *57*(suppl_3), key075.527. https://doi.org/10.1093/rheumatology/key075.527
222. Mackintosh, K. A., Chappel, S. E., Salmon, J., Timperio, A., Ball, K., Brown, H., Macfarlane, S., & Ridgers, N. D. (2019). Parental Perspectives of a Wearable Activity Tracker for Children Younger Than 13 Years: Acceptability and Usability Study. *JMIR mHealth and uHealth*, *7*(11), e13858. https://doi.org/10.2196/13858
223. Brazendale, K., Beets, M. W., Weaver, R. G., Perry, M. W., Tyler, E. B., Hunt, E. T., Decker, L., & Chaput, J.-P. (2019). Comparing measures of free-living sleep in school-aged children. *Sleep Medicine*, *60*, 197–201. https://doi.org/10.1016/j.sleep.2019.04.006
224. Tracy, J. D., Donnelly, T., Sommer, E. C., Heerman, W. J., Barkin, S. L., & Buchowski, M. S. (2021). Identifying bedrest using waist-worn triaxial accelerometers in preschool children. *PLOS ONE*, *16*(1), e0246055. https://doi.org/10.1371/journal.pone.0246055
225. Crooks, N., Alston, L., Nichols, M., Bolton, K. A., Allender, S., Fraser, P., Le, H., Bliss, J., Rennie, C., Orellana, L., & Strugnell, C. (2021). Association between the school physical activity environment, measured and self-reported student physical activity and active transport behaviours in Victoria, Australia. *International Journal of Behavioral Nutrition and Physical Activity*, *18*(1), 79. https://doi.org/10.1186/s12966-021-01151-6
226. Duncan, S., Stewart, T., Mackay, L., Neville, J., Narayanan, A., Walker, C., Berry, S., & Morton, S. (2018). Wear-Time Compliance with a Dual-Accelerometer System for Capturing 24-h Behavioural Profiles in Children and Adults. *International Journal of Environmental Research and Public Health*, *15*(7), 1296. https://doi.org/10.3390/ijerph15071296
227. Gunn, H. E., O’Rourke, F., Dahl, R. E., Goldstein, T. R., Rofey, D. L., Forbes, E. E., & Shaw, D. S. (2019). Young adolescent sleep is associated with parental monitoring. *Sleep Health*, *5*(1), 58–63. https://doi.org/10.1016/j.sleh.2018.09.001
228. Choi, Y., Sadamune, R., Nakamura, Y., Suita, M., Miyakawa, S., & Maeda, S. (2018). The effect of sleep on motor skill learning in young badminton players aged 6–9 years. *Sleep and Biological Rhythms*, *16*(1), 141–147. https://doi.org/10.1007/s41105-017-0136-4
229. Faghy, M. A., Armstrong-Booth, K. E., Staples, V., Duncan, M. J., & Roscoe, C. M. P. (2021). Multi-Component Physical Activity Interventions in the UK Must Consider Determinants of Activity to Increase Effectiveness. *Journal of Functional Morphology and Kinesiology*, *6*(3), 56. https://doi.org/10.3390/jfmk6030056
230. Brazendale, K., Decker, L., T HUNT, E. T. H. A. N., Perry, M. W., Brazendale, A. B., Weaver, R. G., & Beets, M. W. (2019). Validity and wearability of consumer-based fitness trackers in free-living children. *International journal of exercise science*, *12*(5), 471.
231. McWhannell, N., Triggs, C., & Moss, S. (2019). Perceptions and measurement of playtime physical activity in English primary school children: The influence of socioeconomic status. *European Physical Education Review*, *25*(2), 438–455. https://doi.org/10.1177/1356336X17743048
232. Aadland, E., Okely, A. D., & Nilsen, A. K. O. (2022). Trajectories of physical activity and sedentary time in Norwegian children aged 3–9 years: A 5-year longitudinal study. *International Journal of Behavioral Nutrition and Physical Activity*, *19*(1), 67. https://doi.org/10.1186/s12966-022-01286-0
233. Xiu, L., Ekstedt, M., Hagströmer, M., Bruni, O., Bergqvist-Norén, L., & Marcus, C. (2020). Sleep and Adiposity in Children From 2 to 6 Years of Age. *Pediatrics*, *145*(3), e20191420. https://doi.org/10.1542/peds.2019-1420
234. Eichinger, M., Schneider, S., & De Bock, F. (2018). Subjectively and Objectively Assessed Behavioral, Social, and Physical Environmental Correlates of Sedentary Behavior in Preschoolers. *The Journal of Pediatrics*, *199*, 71-78.e3. https://doi.org/10.1016/j.jpeds.2018.04.011
235. Schmutz, E. A., Leeger-Aschmann, C. S., Kakebeeke, T. H., Zysset, A. E., Messerli-Bürgy, N., Stülb, K., Arhab, A., Meyer, A. H., Munsch, S., Puder, J. J., Jenni, O. G., & Kriemler, S. (2020). Motor Competence and Physical Activity in Early Childhood: Stability and Relationship. *Frontiers in Public Health*, *8*, 39. https://doi.org/10.3389/fpubh.2020.00039
