# Supplementary material for: Adherence to Actigraphic Devices in Elementary School–Aged Children: Systematic Review and Meta-Analysis
Source: J Med Internet Res. 2025 Nov 3;27:e79718. doi: 10.2196/79718 (PMC12582557; doi:10.2196/79718)
Supplement: Multimedia Appendix 7 [file jmir-v27-e79718-s007.docx]

| **Multimedia appendix 7. Overview of actigraphic device usage and characteristics in the included studies** | | | | | |
| --- | --- | --- | --- | --- | --- |
| **Brand** | **Frequency of use (participants)** | **Models** | **Primary purpose (%)** | **Protocol duration (short/long, range)** | **Device type** |
| Condor Instruments | 1 (n = 10) | ActTrust | 100% Sleep | 100% Long (14 days) | Research-grade |
| 2M Engineering | 1 (n = 34) | VitaMove | 100% PA | 100% Short (3 days) | Research-grade |
| Actigraph | ^a^152 (n = 12,2056) | GT3X+, GT9X Link, GT3X, GT1M, wGT3X-BT | 83.55% PA, 8.55% Sleep, 7.90% Both | 84.21% Short, 10.52% Long, 5.26% NR (<1 day – 49 days) | Research-grade |
| Activinsights | ^b^13 (n = 7,563) | GENEActiv | 69.23% PA, 7.69% Sleep, 23.08% Both | 53.85% Short, 38.46% Long, 7.69% NR (3 – 9 days) | Research-grade |
| Ambulatory monitoring Inc. | 9 (n = 1,874) | Micro Motionlogger | 88.89% Sleep, 11.11% Both | 88.89% Short, 11.11% Long (3-8 days) | Research-grade |
| Aparito | 1 (n = 12) | Aparito device | 100% PA | 100% Long (91 days) | Commercial |
| Apple | 1 (n = 30) | NR | NR | 100% Short (2 days) | Commercial |
| Axivity | 3 (n = 240) | AX3 | 100% PA | 100% Short (7 days) | Research-grade |
| CamNtech | 3 (n = 1,460) | Actiheart, MotionWatch 8 | 66.67% PA, 33.33% Sleep | 100% Short (6-7 days) | Research-grade |
| Fitbit | ^c^9 (n = 5,319) | Charge HR, Alta HR, Charge 2, Zip, Flex, One | 44.44% PA, 22.22% Sleep, 22.22% Both, 11.11% HR | 22.22% Short, 44.44% Long, 33.33% NR (2 – 140 days) | Commercial |
| Gait Up | 1 (n = 29) | Physilog4 | 100% PA | NR | Commercial |
| Garmin | 2 (n = 295) | Vívofit Jr., Vívofit Jr. 2 | 100% PA | 100% Short (5-7 days) | Commercial |
| Motionlogger | 1 (n = 210) | Octagonal Basic | 100% Sleep | 100% Short (7 days) | Research-grade |
| Movisens | 1 (n = 41) | Move 3 | 100% PA | 100% Short (7 days) | Research-grade |
| Omron | 1 (n = 49) | Active Style Pro | 100% PA | 100% Short (7 days) | Research-grade |
| PAL Technologies | ^d^7 (n = 1,623) | activPAL, activPAL, Micro4 | 100% PA | 71.43% Short, 14.29% Long, 14.29% NR (3-10 days) | Research-grade |
| Philips Respironics | ^e^20 (n = 4,237) | Actical, Actical 2.1, Actiwatch 2, Actiwatch Spectrum, Spectrum 2, Spectrum Plus | 23.81% PA, 52.38% Sleep, 23.81% Both | 61.90% Short; 33.33% Long; 4.76% NR (5-16 days) | Research-grade |
| Polar Active | 1 (n = 49) | NR | 100% PA | 100% Short (7 days) | Commercial |
| RunScribe | 1 (n = 13) | NR | 100% PA | 100% Short (3 days) | Commercial |
| SenseWear | 3 (n = 1227) | Armband 2, Armband Mini, Armband Pro3 | 66.67% PA, 33.33% Sleep | 100% Short (3-7 days) | Commercial |
| SCRIIN | 1 (n = 121) | NR | 100% PA | 100% Short (7 days) | Commercial |
| Suzuken | 2 (n = 187) | Lifecorder GS, Lifecorder | 50% PA, 50% Sleep | 50% Short, 50% Long (7-14 days) | Research-grade |
| UKK Terveyspalvelut Oy | 1 (n = 206) | RM42 | 100% PA | 100% Short (5 days) | Research-grade |
| Withings | 3 (n = 417) | Go activity tracker, Steel HR | 66.67% PA, 33.33% Both | 100% Long (10-28 days) | Commercial |
| ZurichMOVE | 1 (n = 43) | ZurichMOVE sensor modules | 100% PA | 100% Short (7 days) | Research-grade |
| X-Doria International | 1 (n = 36) | KidFit | 100% Both | 100% Long (28 days) | Commercial |
| ^Device and study characteristics are reported per individual device rather than per study. Instances are counted separately for studies that used multiple devices, specifically: aActigraph, 146 sole devices, 6 uses concurrent with another device (n = 462), bActiveinsights, sole device 11, 2 uses concurrent with another device (113), cFitbit, 7 sole devices, 2 uses concurrent with another device (n= 50), dPAL technologies, 4 sole devices, 3 uses concurrent with another device (n = 371), ePhillips Respironics, 19 sole devices, 1 uses concurrent with another device (n=41). PA = Physical activity, Both = Physical activity and Sleep.^ | | | | | |
