# Supplementary material for: Adherence to Actigraphic Devices in Elementary School–Aged Children: Systematic Review and Meta-Analysis
Source: J Med Internet Res. 2025 Nov 3;27:e79718. doi: 10.2196/79718 (PMC12582557; doi:10.2196/79718)
Supplement: Multimedia Appendix 8 [file jmir-v27-e79718-s008.docx]

**Multimedia appendix 8. Protocol wear time distribution across health diagnosis categories**

**
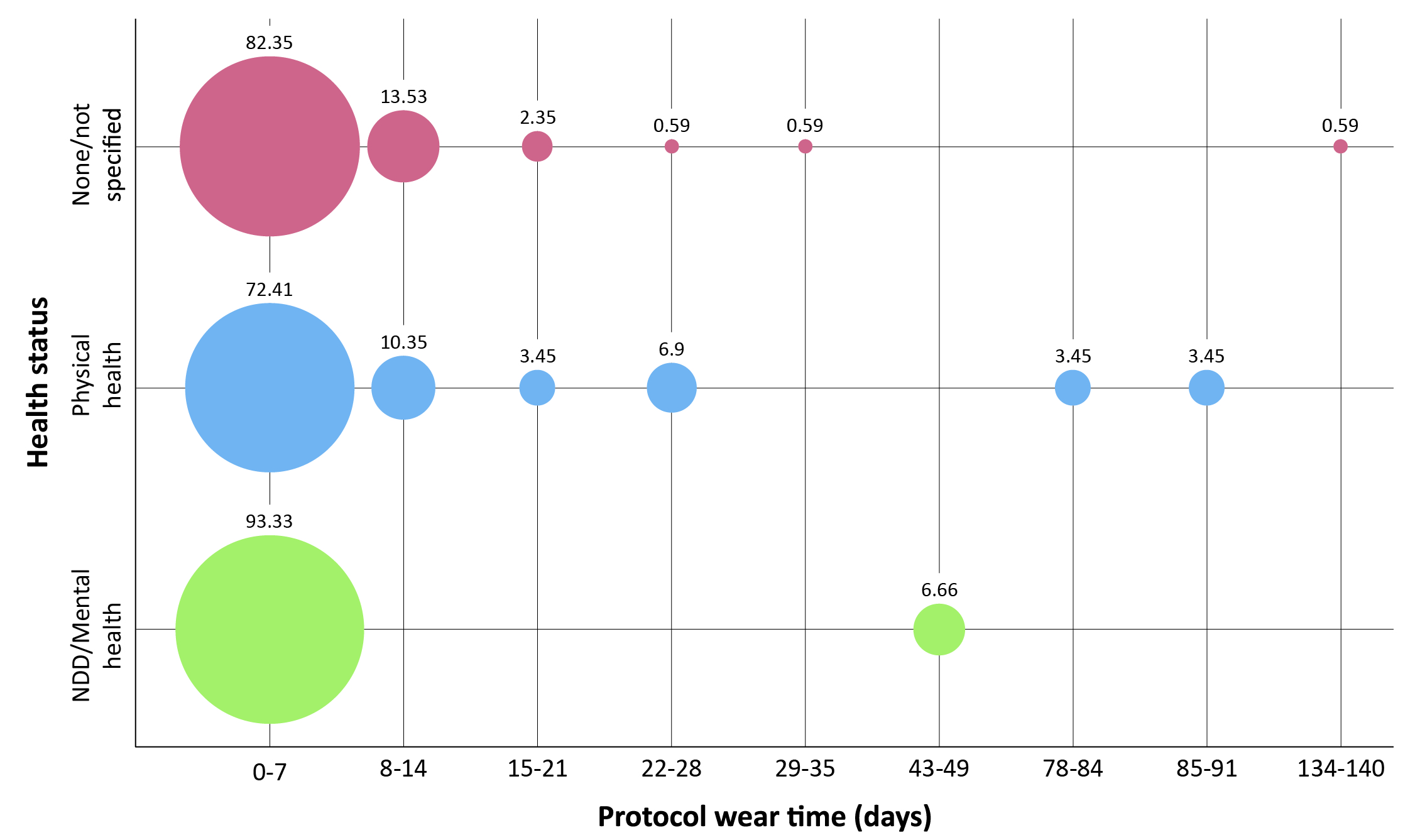
**

**Legend:** interval 0-7 represents short wear time and all other categories represent long wear time. Numbers above each bubble indicate percentages. Abbreviation: Neurodevelopmental Disorder, NDD.
