# Supplementary material for: Adherence to Actigraphic Devices in Elementary School–Aged Children: Systematic Review and Meta-Analysis
Source: J Med Internet Res. 2025 Nov 3;27:e79718. doi: 10.2196/79718 (PMC12582557; doi:10.2196/79718)
Supplement: Multimedia Appendix 9 [file jmir-v27-e79718-s009.docx]

| **Multimedia appendix 9. Quality assessment scores of included studies** | | | | | | | | | |
| --- | --- | --- | --- | --- | --- | --- | --- | --- | --- |
|  | **Criterion** | | | | | | | | **Total** |
| **Source** | **1** | **2** | **3** | **4** | **5** | **6** | **7** | **8** | **(0-8)** |
| Migueles, 2021 | 1 | 1 | 0 | 1 | 1 | 0 | 1 | 0 | 5 |
| Fairclough, 2019 | 1 | 1 | 1 | 1 | 1 | 0 | 0 | 1 | 6 |
| Beltran-Valls, 2019 | 1 | 1 | 1 | 1 | 1 | 0 | 0 | 0 | 5 |
| Pearce, 2018 | 1 | 0 | 0 | 1 | 1 | 0 | 0 | 0 | 3 |
| Silva, 2018 | 1 | 1 | 1 | 1 | 1 | 0 | 0 | 0 | 5 |
| Adank, 2021 | 1 | 1 | 1 | 1 | 1 | 1 | 0 | 0 | 6 |
| Alder, 2023 | 1 | 1 | 1 | 1 | 1 | 1 | 0 | 1 | 7 |
| Allen, 2019 | 1 | 1 | 1 | 1 | 1 | 0 | 0 | 0 | 5 |
| Anselma, 2023 | 1 | 1 | 1 | 1 | 1 | 0 | 0 | 0 | 5 |
| Bagley, 2018 | 1 | 1 | 1 | 1 | 1 | 0 | 0 | 1 | 6 |
| Bedell, 2022 | 1 | 0 | 1 | 1 | 1 | 1 | 0 | 1 | 6 |
| Beemer, 2020 | 1 | 1 | 1 | 1 | 1 | 0 | 0 | 1 | 6 |
| Bejarano, 2021 | 1 | 1 | 1 | 1 | 1 | 1 | 0 | 0 | 6 |
| Bekelman, 2021 | 1 | 1 | 1 | 1 | 1 | 0 | 0 | 1 | 6 |
| Berge, 2021 | 1 | 1 | 1 | 1 | 1 | 1 | 0 | 1 | 7 |
| Bolger, 2019 | 1 | 1 | 1 | 1 | 1 | 0 | 0 | 0 | 5 |
| Brønd, 2019 | 1 | 1 | 1 | 1 | 1 | 1 | 0 | 0 | 6 |
| Brudy, 2020 | 1 | 1 | 1 | 1 | 1 | 1 | 0 | 1 | 7 |
| Caserta, 2022 | 1 | 1 | 1 | 1 | 1 | 1 | 0 | 1 | 7 |
| Cassim, 2021 | 1 | 1 | 1 | 1 | 1 | 0 | 1 | 1 | 7 |
| Chen, 2020 | 1 | 1 | 1 | 1 | 1 | 0 | 0 | 1 | 6 |
| Christian, 2020 | 1 | 1 | 1 | 1 | 1 | 0 | 0 | 1 | 6 |
| Clark, 2019 | 1 | 1 | 1 | 1 | 1 | 0 | 0 | 1 | 6 |
| Costa, 2019 | 1 | 1 | 1 | 1 | 1 | 1 | 0 | 0 | 6 |
| Cradock, 2019 | 1 | 1 | 1 | 1 | 1 | 1 | 0 | 1 | 7 |
| Draper, 2019 | 1 | 1 | 1 | 1 | 1 | 0 | 0 | 0 | 5 |
| Duck, 2021 | 1 | 1 | 1 | 1 | 1 | 0 | 0 | 1 | 6 |
| Evenson, 2019 | 1 | 1 | 1 | 1 | 1 | 1 | 0 | 0 | 6 |
| Fang, 2020 | 1 | 1 | 1 | 1 | 1 | 0 | 0 | 1 | 6 |
| Fraysse, 2019 | 1 | 1 | 1 | 1 | 1 | 1 | 1 | 0 | 7 |
| Gaser, 2022 | 1 | 1 | 1 | 1 | 1 | 0 | 0 | 0 | 5 |
| Gerber, 2021 | 1 | 1 | 1 | 1 | 1 | 0 | 0 | 1 | 6 |
| Hall, 2019 | 1 | 1 | 1 | 1 | 1 | 1 | 0 | 0 | 6 |
| Holzhausen, 2020 | 1 | 1 | 1 | 1 | 1 | 0 | 1 | 1 | 7 |
| Hulst, 2023 | 1 | 1 | 1 | 1 | 1 | 0 | 1 | 1 | 7 |
| Joensuu, 2018 | 1 | 1 | 1 | 1 | 1 | 0 | 0 | 1 | 6 |
| Kattelmann, 2019 | 1 | 1 | 0 | 1 | 1 | 1 | 0 | 0 | 5 |
| Kippe, 2022 | 1 | 1 | 1 | 1 | 1 | 0 | 0 | 0 | 5 |
| Knox, 2019 | 1 | 0 | 0 | 1 | 1 | 1 | 0 | 0 | 4 |
| Li, 2021 | 1 | 1 | 1 | 1 | 1 | 1 | 0 | 0 | 6 |
| Ludwig & Rauch, 2018 | 1 | 1 | 1 | 1 | 1 | 1 | 0 | 0 | 6 |
| Mazza, 2020 | 1 | 1 | 1 | 1 | 1 | 0 | 1 | 1 | 7 |
| McCrorie, 2018 | 1 | 0 | 1 | 1 | 1 | 0 | 0 | 0 | 4 |
| Riiser, 2020 | 1 | 1 | 1 | 1 | 1 | 1 | 0 | 1 | 7 |
| Hartman, 2020 | 1 | 1 | 1 | 1 | 1 | 0 | 0 | 1 | 6 |
| Gråstén & Yli-Piipari, 2019 | 1 | 1 | 0 | 1 | 1 | 0 | 0 | 0 | 4 |
| Gråstén, 2021 | 1 | 1 | 1 | 1 | 1 | 0 | 0 | 0 | 5 |
| Wiersma, 2019 | 1 | 1 | 1 | 1 | 1 | 0 | 0 | 1 | 6 |
| Williams, 2022 | 1 | 1 | 1 | 1 | 1 | 1 | 0 | 1 | 7 |
| Verjans-Janssen, 2020 | 1 | 1 | 1 | 1 | 1 | 0 | 0 | 0 | 5 |
| Chan, 2019 | 1 | 0 | 1 | 1 | 1 | 0 | 0 | 0 | 4 |
| St Laurent, 2022 | 1 | 1 | 1 | 1 | 1 | 0 | 1 | 1 | 7 |
| Wang, 2022 | 1 | 1 | 1 | 1 | 1 | 1 | 0 | 0 | 6 |
| Yu, 2021 | 1 | 1 | 1 | 1 | 1 | 0 | 1 | 1 | 7 |
| Velde, 2021 | 1 | 1 | 1 | 1 | 1 | 0 | 0 | 0 | 5 |
| Aguilar-Farias, 2020 | 1 | 1 | 1 | 1 | 1 | 0 | 0 | 1 | 6 |
| Clevenger, 2022 | 1 | 1 | 1 | 1 | 1 | 0 | 0 | 0 | 5 |
| Kjellberg Olofsson, 2023 | 1 | 1 | 1 | 1 | 1 | 1 | 0 | 0 | 6 |
| Callaghan, 2021 | 1 | 1 | 0 | 1 | 1 | 0 | 0 | 0 | 4 |
| Oakley, 2021 | 1 | 1 | 1 | 1 | 1 | 0 | 1 | 0 | 6 |
| Palmer, 2018 | 1 | 1 | 0 | 1 | 1 | 0 | 1 | 1 | 6 |
| Dahlgren, 2021 | 1 | 1 | 1 | 1 | 1 | 0 | 0 | 0 | 5 |
| Skjåkødegård, 2020 | 1 | 1 | 1 | 1 | 1 | 0 | 1 | 1 | 7 |
| Harrex, 2018 | 1 | 1 | 1 | 1 | 1 | 0 | 0 | 1 | 6 |
| Park, 2018 | 1 | 1 | 1 | 1 | 1 | 0 | 0 | 0 | 5 |
| Van Kann, 2019 | 1 | 1 | 1 | 1 | 1 | 0 | 0 | 0 | 5 |
| Chen, 2023 | 1 | 1 | 1 | 1 | 1 | 0 | 0 | 1 | 6 |
| Chong, 2021 | 1 | 1 | 1 | 1 | 1 | 0 | 0 | 0 | 5 |
| Talarico & Janssen, 2018 | 1 | 1 | 1 | 1 | 1 | 1 | 1 | 0 | 7 |
| Herbert, 2022 | 1 | 1 | 1 | 1 | 1 | 0 | 0 | 0 | 5 |
| Salin, 2019 | 1 | 1 | 1 | 1 | 1 | 1 | 0 | 0 | 6 |
| Li, 2021 | 1 | 1 | 1 | 1 | 1 | 1 | 1 | 1 | 8 |
| Lai, 2020 | 1 | 1 | 1 | 1 | 1 | 0 | 0 | 0 | 5 |
| Willoughby, 2018 | 1 | 1 | 1 | 1 | 1 | 1 | 0 | 1 | 7 |
| Wyszyńska, 2021 | 1 | 1 | 1 | 1 | 1 | 0 | 0 | 1 | 6 |
| Xu & Qi, 2022 | 1 | 1 | 1 | 1 | 1 | 1 | 0 | 1 | 7 |
| Yang, 2019 | 1 | 1 | 1 | 1 | 1 | 1 | 0 | 1 | 7 |
| Yoong, 2019 | 1 | 1 | 1 | 1 | 1 | 1 | 0 | 1 | 7 |
| Zhang, 2020 | 1 | 1 | 1 | 1 | 1 | 0 | 0 | 1 | 6 |
| So, 2021 | 1 | 1 | 1 | 1 | 1 | 0 | 0 | 1 | 6 |
| Sprengeler, 2020 | 1 | 1 | 1 | 1 | 1 | 1 | 0 | 0 | 6 |
| Abdollahi, 2024 | 1 | 1 | 1 | 1 | 1 | 0 | 1 | 1 | 7 |
| Downing, 2021 | 1 | 1 | 1 | 1 | 1 | 1 | 0 | 0 | 6 |
| Mücke, 2021 | 1 | 1 | 1 | 1 | 1 | 0 | 0 | 1 | 6 |
| Beunders, 2023 | 1 | 1 | 1 | 1 | 1 | 0 | 1 | 1 | 7 |
| McGarty, 2021 | 1 | 1 | 1 | 1 | 1 | 0 | 0 | 1 | 6 |
| McMullen, 2019 | 1 | 1 | 1 | 1 | 1 | 0 | 0 | 1 | 6 |
| Mughal, 2020 | 1 | 1 | 1 | 1 | 1 | 0 | 1 | 1 | 7 |
| Nakabazzi, 2020 | 1 | 1 | 1 | 1 | 1 | 0 | 0 | 1 | 6 |
| Nathan, 2020 | 1 | 1 | 1 | 1 | 1 | 1 | 0 | 0 | 6 |
| Patton, 2022 | 1 | 1 | 1 | 1 | 1 | 1 | 0 | 0 | 6 |
| Crotti, 2021 | 1 | 1 | 1 | 1 | 1 | 1 | 1 | 1 | 8 |
| Henriques-Neto, 2021 | 1 | 1 | 1 | 1 | 1 | 0 | 0 | 0 | 5 |
| Kobel, 2020 | 1 | 1 | 1 | 1 | 1 | 0 | 0 | 0 | 5 |
| Yu, 2019 | 1 | 1 | 1 | 1 | 1 | 0 | 0 | 1 | 6 |
| Joschtel, 2019 | 1 | 1 | 1 | 1 | 1 | 0 | 1 | 0 | 6 |
| Kwon, 2022 | 1 | 1 | 1 | 1 | 1 | 1 | 0 | 0 | 6 |
| Manyanga, 2019 | 1 | 1 | 1 | 1 | 1 | 0 | 0 | 0 | 5 |
| Cremone, 2018 | 1 | 1 | 0 | 1 | 1 | 0 | 0 | 0 | 4 |
| Higgins, 2021 | 1 | 1 | 1 | 1 | 1 | 0 | 0 | 1 | 6 |
| Schroeder, 2020 | 1 | 1 | 1 | 1 | 1 | 0 | 0 | 1 | 6 |
| Tsuda, 2020 | 1 | 1 | 1 | 1 | 1 | 0 | 0 | 1 | 6 |
| Wang, 2022 | 1 | 1 | 1 | 1 | 1 | 0 | 0 | 1 | 6 |
| Zask, 2023 | 1 | 1 | 1 | 1 | 1 | 0 | 0 | 0 | 5 |
| Wright, 2020 | 1 | 1 | 1 | 1 | 1 | 0 | 1 | 1 | 7 |
| Brazendale, 2018 | 1 | 1 | 1 | 1 | 1 | 1 | 1 | 1 | 8 |
| Riso, 2018 | 1 | 1 | 1 | 1 | 1 | 1 | 0 | 0 | 6 |
| Yamakita, 2019 | 1 | 1 | 1 | 1 | 1 | 0 | 0 | 1 | 6 |
| Leppänen, 2022 | 1 | 1 | 1 | 1 | 1 | 0 | 0 | 0 | 5 |
| Tan, 2022 | 1 | 1 | 1 | 1 | 1 | 0 | 0 | 1 | 6 |
| McLellan, 2020 | 1 | 1 | 1 | 1 | 1 | 1 | 0 | 1 | 7 |
| Winsor, 2023 | 1 | 1 | 1 | 1 | 1 | 0 | 0 | 1 | 6 |
| Lott, 2021 | 1 | 1 | 1 | 1 | 1 | 1 | 0 | 0 | 6 |
| Kariippanon, 2022 | 1 | 1 | 1 | 1 | 1 | 0 | 0 | 0 | 5 |
| Lu, 2022 | 1 | 1 | 1 | 1 | 1 | 0 | 0 | 0 | 5 |
| Vyhlídal, 2022 | 1 | 1 | 1 | 1 | 1 | 0 | 0 | 0 | 5 |
| da Costa, 2022 | 1 | 1 | 1 | 1 | 1 | 1 | 0 | 0 | 6 |
| Robbins, 2020 | 1 | 1 | 0 | 1 | 1 | 0 | 0 | 0 | 4 |
| Schwarzfischer, 2018 | 1 | 1 | 1 | 1 | 1 | 0 | 0 | 0 | 5 |
| Lambrechtse, 2021 | 1 | 1 | 0 | 1 | 1 | 0 | 0 | 0 | 4 |
| Sherry, 2019 | 1 | 1 | 0 | 1 | 1 | 0 | 0 | 0 | 4 |
| Dumuid, 2021 | 1 | 1 | 1 | 1 | 1 | 1 | 1 | 1 | 8 |
| Abel, 2018 | 1 | 0 | 1 | 1 | 1 | 0 | 1 | 1 | 6 |
| Kidokoro, 2019 | 1 | 1 | 1 | 1 | 1 | 0 | 0 | 0 | 5 |
| Kallio, 2020 | 1 | 1 | 1 | 1 | 1 | 0 | 0 | 0 | 5 |
| Ranum, 2019 | 1 | 1 | 1 | 1 | 0 | 0 | 0 | 1 | 5 |
| Swartz, 2019 | 1 | 1 | 1 | 1 | 1 | 0 | 1 | 1 | 7 |
| Santiago-Rodríguez, 2022 | 1 | 1 | 1 | 1 | 1 | 0 | 0 | 0 | 5 |
| Sánchez-Oliva, 2020 | 1 | 1 | 1 | 1 | 1 | 0 | 0 | 0 | 5 |
| Salmon, 2023 | 1 | 1 | 1 | 0 | 1 | 0 | 0 | 0 | 4 |
| Gerber, 2019 | 1 | 1 | 1 | 0 | 1 | 0 | 0 | 1 | 5 |
| Mitchell, 2018 | 1 | 1 | 1 | 1 | 1 | 0 | 0 | 0 | 5 |
| Bloemen, 2019 | 1 | 1 | 1 | 1 | 1 | 1 | 0 | 0 | 6 |
| Loram, 2024 | 1 | 1 | 1 | 0 | 1 | 0 | 0 | 0 | 4 |
| Price, 2018 | 1 | 1 | 0 | 0 | 1 | 1 | 0 | 1 | 5 |
| Pate, 2019 | 1 | 0 | 1 | 1 | 1 | 0 | 0 | 0 | 4 |
| Parry, 2019 | 1 | 1 | 1 | 1 | 0 | 0 | 0 | 0 | 4 |
| Nigg, 2021 | 1 | 1 | 1 | 1 | 1 | 1 | 0 | 0 | 6 |
| Naya, 2021 | 1 | 1 | 1 | 1 | 0 | 0 | 0 | 1 | 5 |
| Mora-González, 2019 | 1 | 1 | 1 | 1 | 0 | 0 | 0 | 1 | 5 |
| Molina-García, 2021 | 1 | 0 | 0 | 0 | 1 | 0 | 0 | 1 | 3 |
| Miadich, 2019 | 1 | 1 | 1 | 1 | 0 | 1 | 1 | 0 | 6 |
| Jakubec, 2020 | 1 | 1 | 1 | 1 | 1 | 1 | 1 | 1 | 8 |
| Sturm, 2021 | 1 | 0 | 1 | 1 | 1 | 1 | 0 | 1 | 6 |
| Sinisterra, 2020 | 1 | 0 | 0 | 0 | 1 | 0 | 0 | 0 | 2 |
| Silva, 2018 | 1 | 1 | 1 | 1 | 1 | 0 | 0 | 0 | 5 |
| Bachner, 2020 | 1 | 1 | 1 | 1 | 1 | 1 | 0 | 1 | 7 |
| Nyberg, 2020 | 1 | 1 | 1 | 1 | 1 | 1 | 0 | 0 | 6 |
| Franceschi, 2022 | 1 | 1 | 1 | 1 | 1 | 0 | 1 | 1 | 7 |
| Cabanas-Sánchez, 2018 | 1 | 1 | 1 | 1 | 1 | 0 | 0 | 1 | 6 |
| Armstrong, 2021 | 1 | 1 | 1 | 1 | 0 | 0 | 0 | 0 | 4 |
| Bringolf-Isler, 2018 | 1 | 1 | 1 | 1 | 1 | 1 | 0 | 1 | 7 |
| Sprengeler, 2021 | 1 | 1 | 1 | 1 | 1 | 0 | 1 | 0 | 6 |
| De Meester, 2018 | 1 | 1 | 1 | 1 | 0 | 0 | 0 | 1 | 5 |
| Clemes, 2020 | 1 | 1 | 1 | 1 | 1 | 0 | 1 | 0 | 6 |
| Innerd, 2019 | 1 | 1 | 1 | 1 | 1 | 0 | 1 | 1 | 7 |
| Shoesmith, 2020 | 1 | 1 | 1 | 1 | 1 | 0 | 0 | 0 | 5 |
| Buchan & Maylor, 2023 | 1 | 1 | 1 | 1 | 1 | 1 | 0 | 1 | 7 |
| Strugnell, 2023 | 1 | 1 | 1 | 1 | 1 | 1 | 0 | 0 | 6 |
| Seljebotn, 2019 | 1 | 1 | 1 | 1 | 1 | 0 | 0 | 0 | 5 |
| Sacheck, 2021 | 1 | 1 | 1 | 1 | 1 | 0 | 1 | 0 | 6 |
| Trickett, 2019 | 1 | 1 | 1 | 1 | 1 | 1 | 1 | 1 | 8 |
| Reedman, 2019 | 1 | 1 | 1 | 1 | 1 | 0 | 1 | 1 | 7 |
| Braaksma, 2022 | 1 | 0 | 0 | 1 | 0 | 0 | 1 | 1 | 4 |
| Philbrook, 2022 | 1 | 1 | 1 | 1 | 0 | 0 | 1 | 1 | 6 |
| Thorpe, 2021 | 1 | 1 | 1 | 1 | 0 | 0 | 0 | 1 | 5 |
| Traube, 2020 | 1 | 1 | 0 | 1 | 0 | 0 | 0 | 0 | 3 |
| Crowe, 2021 | 1 | 1 | 0 | 1 | 0 | 1 | 0 | 0 | 4 |
| Woods, 2018 | 1 | 1 | 1 | 1 | 0 | 1 | 0 | 1 | 6 |
| Noonan, 2019 | 1 | 1 | 0 | 1 | 0 | 0 | 0 | 1 | 4 |
| Ovans, 2018 | 1 | 1 | 0 | 1 | 0 | 0 | 0 | 1 | 4 |
| Quirk, 2020 | 1 | 1 | 0 | 1 | 0 | 0 | 0 | 1 | 4 |
| Roth, 2019 | 0 | 1 | 0 | 1 | 0 | 0 | 0 | 1 | 3 |
| Esbensen, 2018. | 1 | 0 | 1 | 1 | 0 | 0 | 1 | 0 | 4 |
| Belcher, 2021 | 1 | 1 | 1 | 1 | 1 | 1 | 0 | 0 | 6 |
| Bartholomew, 2018 | 1 | 1 | 1 | 1 | 0 | 1 | 0 | 1 | 6 |
| Burford, 2022 | 1 | 1 | 0 | 1 | 0 | 1 | 0 | 1 | 5 |
| Giddens, 2022 | 1 | 0 | 0 | 0 | 1 | 0 | 0 | 1 | 3 |
| Grant, 2020 | 1 | 1 | 1 | 1 | 0 | 1 | 0 | 1 | 6 |
| Hartikainen, 2022 | 1 | 1 | 0 | 1 | 0 | 1 | 1 | 1 | 6 |
| Lecarie, 2022 | 1 | 1 | 1 | 1 | 0 | 1 | 1 | 1 | 7 |
| Breitenstein, 2021 | 1 | 1 | 1 | 1 | 0 | 0 | 1 | 1 | 6 |
| Finkelstein, 2020 | 1 | 1 | 1 | 1 | 1 | 0 | 0 | 1 | 6 |
| Merbler, 2018 | 1 | 1 | 1 | 1 | 0 | 1 | 1 | 1 | 7 |
| Bergqvist-Norén, 2022 | 1 | 1 | 1 | 1 | 1 | 0 | 0 | 1 | 6 |
| Rast, 2022 | 1 | 1 | 1 | 1 | 0 | 0 | 1 | 1 | 6 |
| Løndal, 2020 | 1 | 0 | 1 | 0 | 0 | 1 | 0 | 1 | 4 |
| Lokhandwala, 2021 | 1 | 1 | 1 | 1 | 1 | 1 | 1 | 1 | 8 |
| Lindhiem, 2022 | 1 | 0 | 1 | 1 | 0 | 0 | 1 | 1 | 5 |
| Kahn, 2020 | 1 | 1 | 0 | 1 | 0 | 1 | 0 | 0 | 4 |
| Johnstone, 2019 | 1 | 1 | 1 | 1 | 1 | 1 | 1 | 0 | 7 |
| Huertas-Delgado, 2021 | 1 | 1 | 1 | 1 | 0 | 0 | 0 | 1 | 5 |
| Heikkila, 2022 | 1 | 1 | 1 | 1 | 0 | 0 | 1 | 1 | 6 |
| Salway, 2019 | 1 | 0 | 1 | 1 | 1 | 0 | 0 | 0 | 4 |
| Barnett, 2018 | 1 | 0 | 1 | 1 | 0 | 0 | 0 | 0 | 3 |
| Bartelink, 2019 | 1 | 1 | 1 | 1 | 1 | 0 | 0 | 0 | 5 |
| Borghese, 2018 | 1 | 1 | 1 | 1 | 0 | 0 | 1 | 1 | 6 |
| Larocque Chevalier, 2020 | 1 | 1 | 0 | 1 | 0 | 0 | 0 | 1 | 4 |
| Clevenger, 2022 | 1 | 1 | 0 | 1 | 0 | 0 | 0 | 0 | 3 |
| Kruizinga, 2021 | 1 | 0 | 0 | 1 | 0 | 1 | 0 | 1 | 4 |
| Kruizinga, 2022 | 1 | 0 | 1 | 1 | 0 | 0 | 0 | 0 | 3 |
| Jaser, 2021 | 1 | 0 | 1 | 1 | 0 | 0 | 1 | 1 | 5 |
| Gotte, 2018 | 1 | 1 | 0 | 1 | 0 | 0 | 0 | 1 | 4 |
| Ha, 2022 | 1 | 1 | 1 | 1 | 1 | 1 | 0 | 1 | 7 |
| Leppänen, 2019 | 1 | 1 | 1 | 1 | 1 | 0 | 0 | 0 | 5 |
| Rhodes, 2019 | 1 | 1 | 1 | 1 | 1 | 1 | 1 | 1 | 8 |
| Rodríguez-Rodríguez, 2020 | 1 | 1 | 1 | 0 | 0 | 0 | 0 | 1 | 4 |
| Willeboordse, 2022 | 0 | 0 | 0 | 0 | 0 | 0 | 0 | 0 | 0 |
| Jago, 2020 | 1 | 1 | 1 | 1 | 1 | 0 | 0 | 0 | 5 |
| Dishman, 2019 | 1 | 0 | 1 | 1 | 1 | 1 | 0 | 1 | 6 |
| Dunton, 2022 | 1 | 1 | 1 | 1 | 1 | 0 | 0 | 1 | 6 |
| Caillaud, 2022 | 1 | 1 | 1 | 1 | 0 | 0 | 0 | 1 | 5 |
| Verloigne, 2018 | 1 | 1 | 1 | 1 | 1 | 0 | 0 | 0 | 5 |
| Dalene, 2018 | 1 | 1 | 1 | 1 | 1 | 0 | 0 | 0 | 5 |
| Pedersen, 2022 | 1 | 1 | 1 | 1 | 1 | 0 | 0 | 0 | 5 |
| Padmapriya, 2021 | 1 | 1 | 1 | 1 | 1 | 0 | 0 | 0 | 5 |
| Verswijveren, 2022 | 1 | 1 | 1 | 1 | 1 | 0 | 0 | 0 | 5 |
| Gantelius, 2022 | 1 | 1 | 1 | 1 | 0 | 0 | 1 | 1 | 6 |
| Fishbein, 2018 | 1 | 1 | 0 | 0 | 0 | 0 | 0 | 0 | 2 |
| Goldschmidt, 2020 | 1 | 1 | 1 | 1 | 0 | 0 | 1 | 0 | 5 |
| Harbottle, 2018 | 1 | 0 | 1 | 1 | 0 | 0 | 0 | 0 | 3 |
| Mackintosh, 2019 | 1 | 1 | 0 | 1 | 0 | 1 | 0 | 0 | 4 |
| Brazendale, 2019 | 1 | 1 | 0 | 0 | 0 | 1 | 0 | 0 | 3 |
| Tracy, 2021 | 1 | 0 | 1 | 1 | 1 | 0 | 1 | 0 | 5 |
| Crooks, 2021. | 1 | 1 | 1 | 1 | 1 | 0 | 0 | 0 | 5 |
| Duncan, 2018 | 1 | 1 | 1 | 1 | 0 | 1 | 0 | 0 | 5 |
| Gunn, 2019 | 1 | 1 | 0 | 1 | 1 | 1 | 0 | 0 | 5 |
| Choi, 2018 | 1 | 1 | 1 | 1 | 0 | 0 | 1 | 1 | 6 |
| Faghy, 2021 | 1 | 1 | 1 | 1 | 0 | 0 | 0 | 0 | 4 |
| Brazendale, 2018 | 1 | 1 | 1 | 1 | 0 | 1 | 1 | 0 | 6 |
| McWhannell, 2019 | 1 | 1 | 1 | 1 | 0 | 1 | 0 | 0 | 5 |
| Aadland, 2022 | 1 | 1 | 1 | 1 | 1 | 0 | 0 | 0 | 5 |
| Xiu, 2020 | 1 | 1 | 1 | 1 | 1 | 0 | 0 | 0 | 5 |
| Eichinger, 2018 | 1 | 0 | 1 | 1 | 1 | 0 | 0 | 0 | 4 |
| Schmutz, 2020 | 1 | 1 | 1 | 0 | 1 | 0 | 0 | 0 | 4 |
| N (%) of studies scoring 1 | 233 (99.15) | 210 (89.36) | 201 (85.53) | 222 (94.47) | 182 (77.45) | 75 (31.92) | 52 (22.13) | 116 (49.36) | 8 (3.40) |
