# Supplementary material for: Adherence to Actigraphic Devices in Elementary School–Aged Children: Systematic Review and Meta-Analysis
Source: J Med Internet Res. 2025 Nov 3;27:e79718. doi: 10.2196/79718 (PMC12582557; doi:10.2196/79718)
Supplement: Multimedia Appendix 10 [file jmir-v27-e79718-s010.docx]

**Multimedia appendix 10. Individual and pooled adherence prevalence estimates**


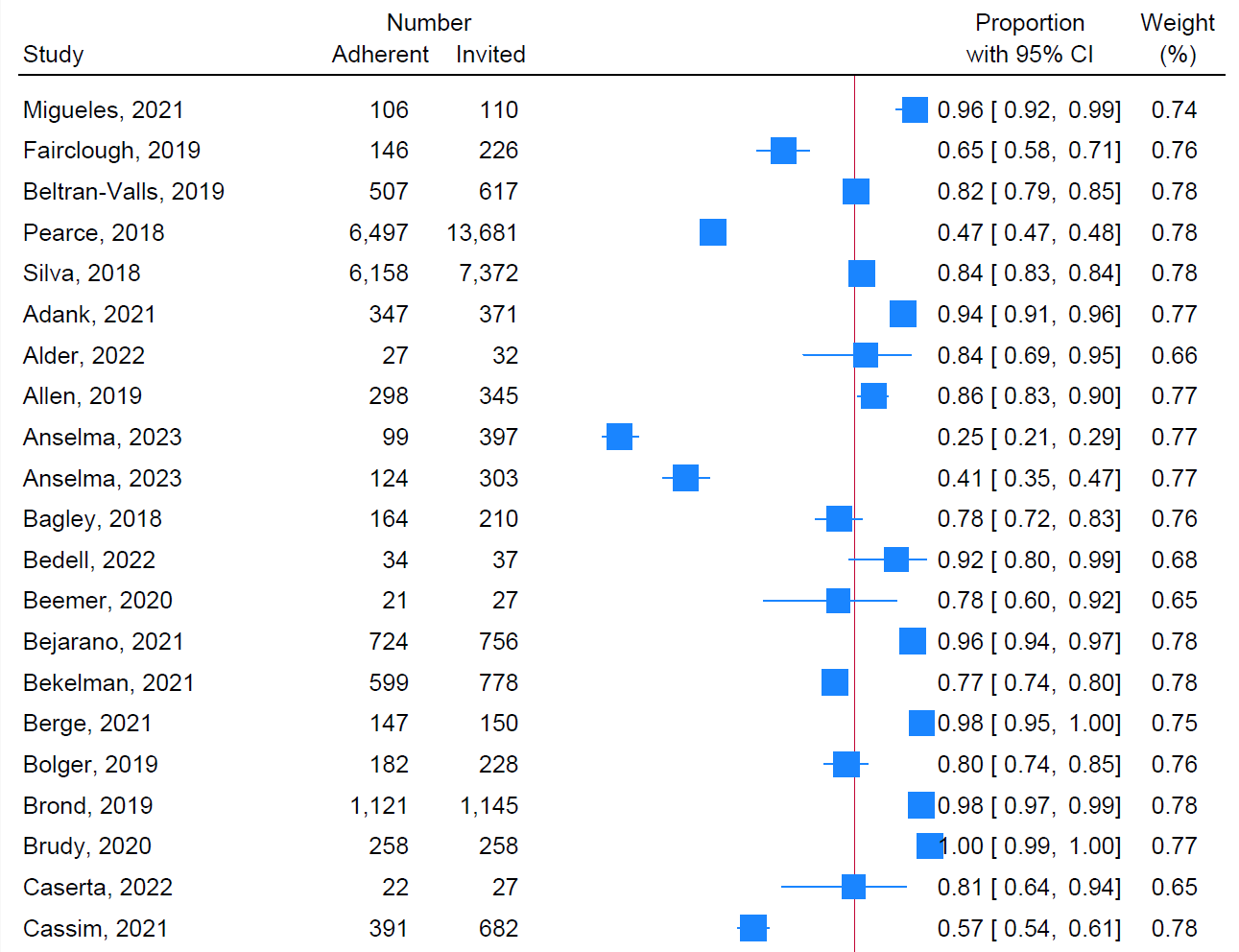

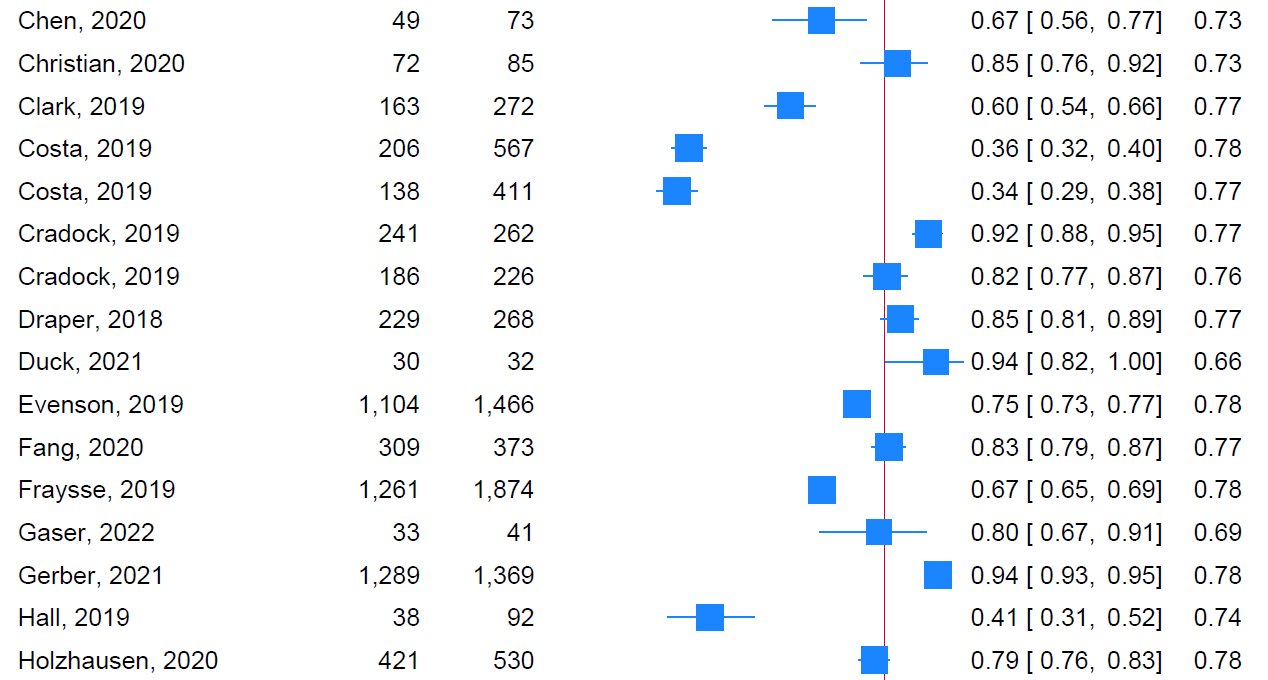

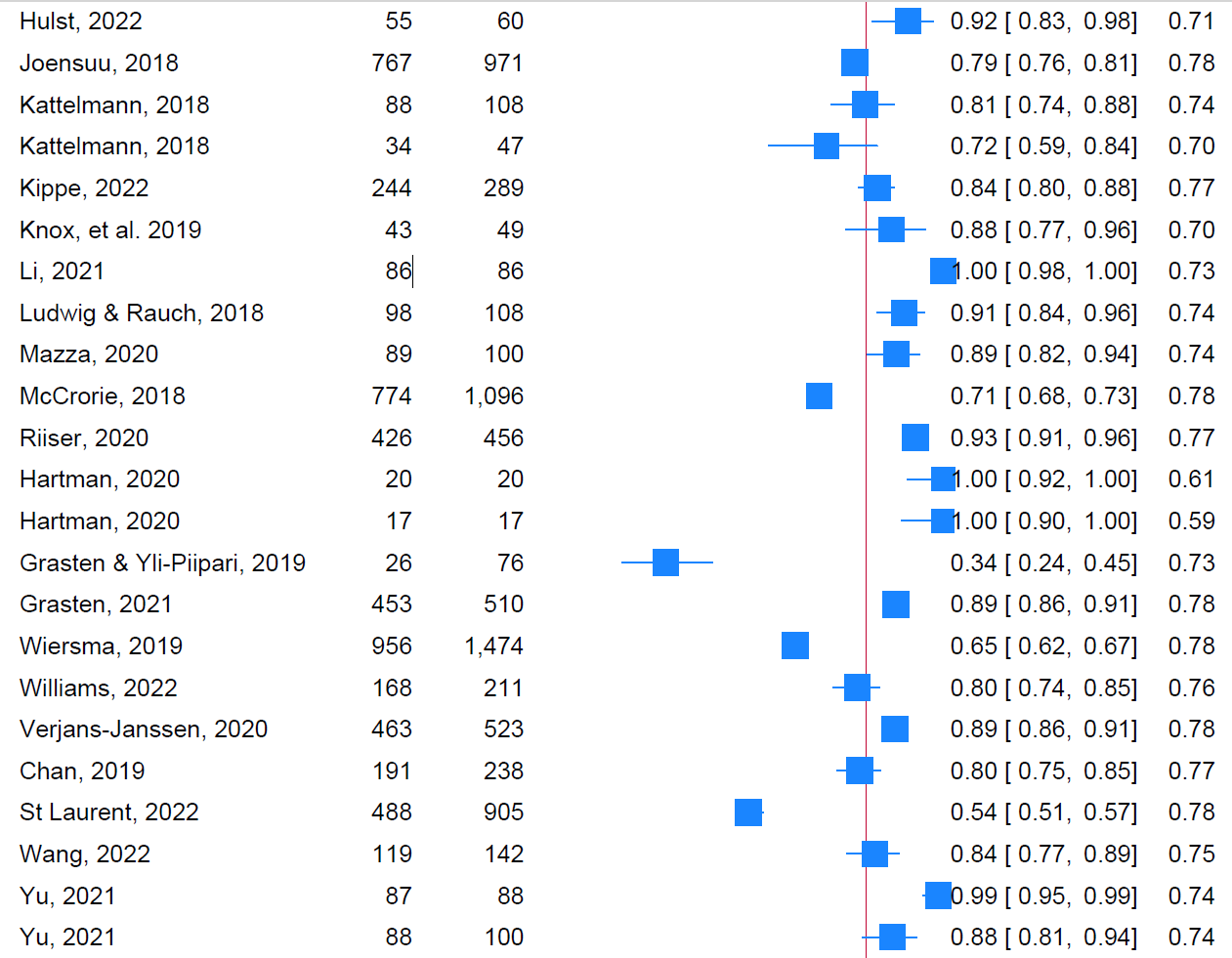

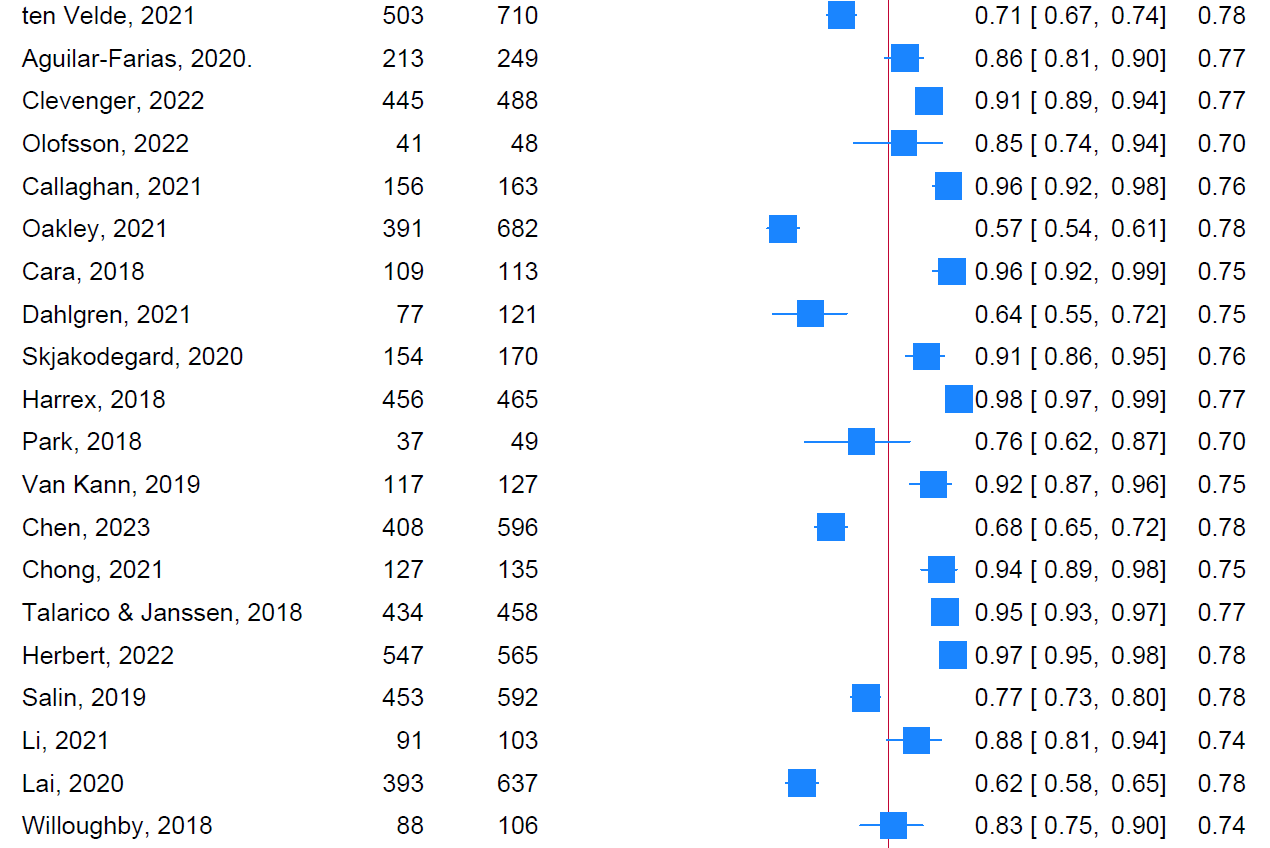

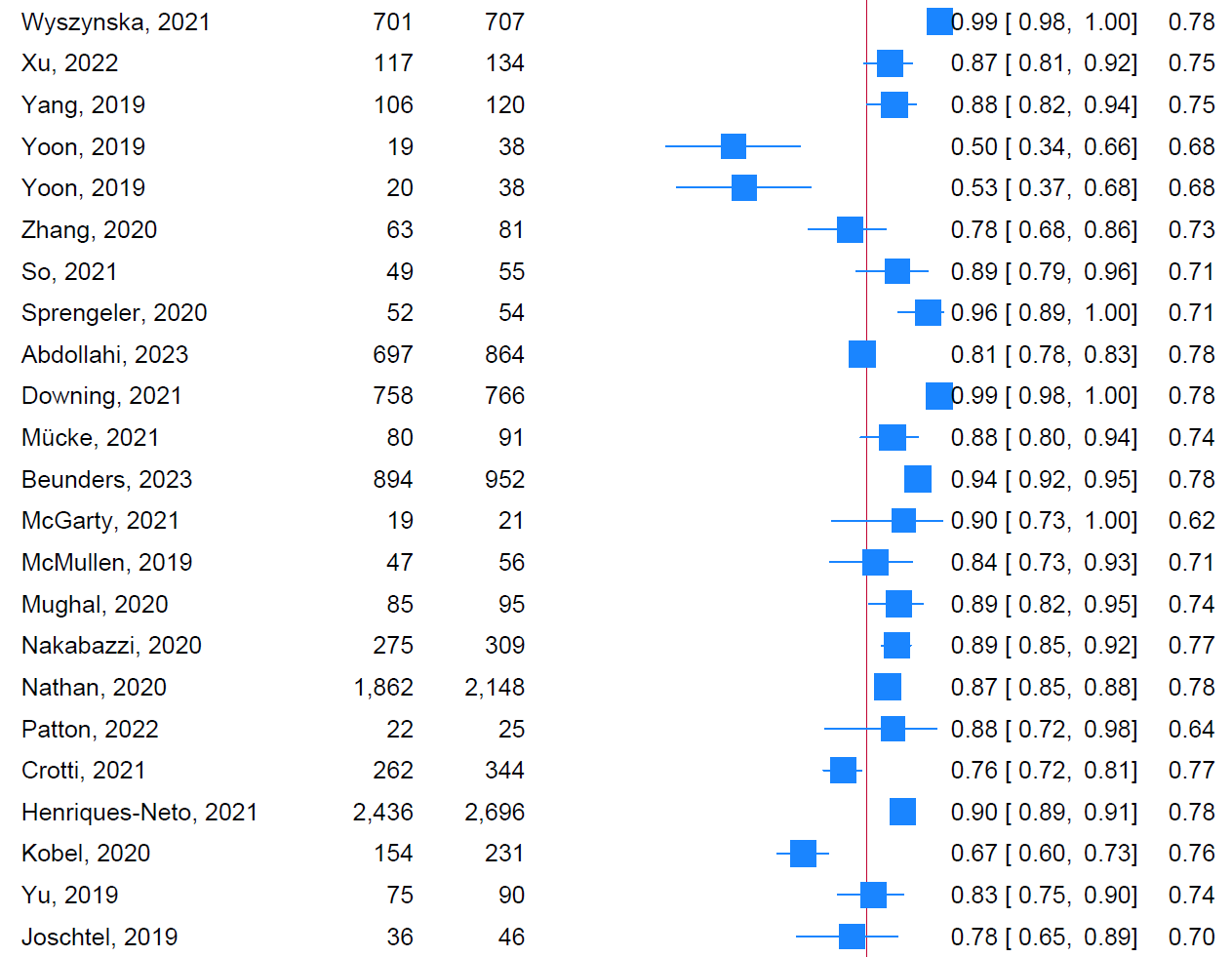

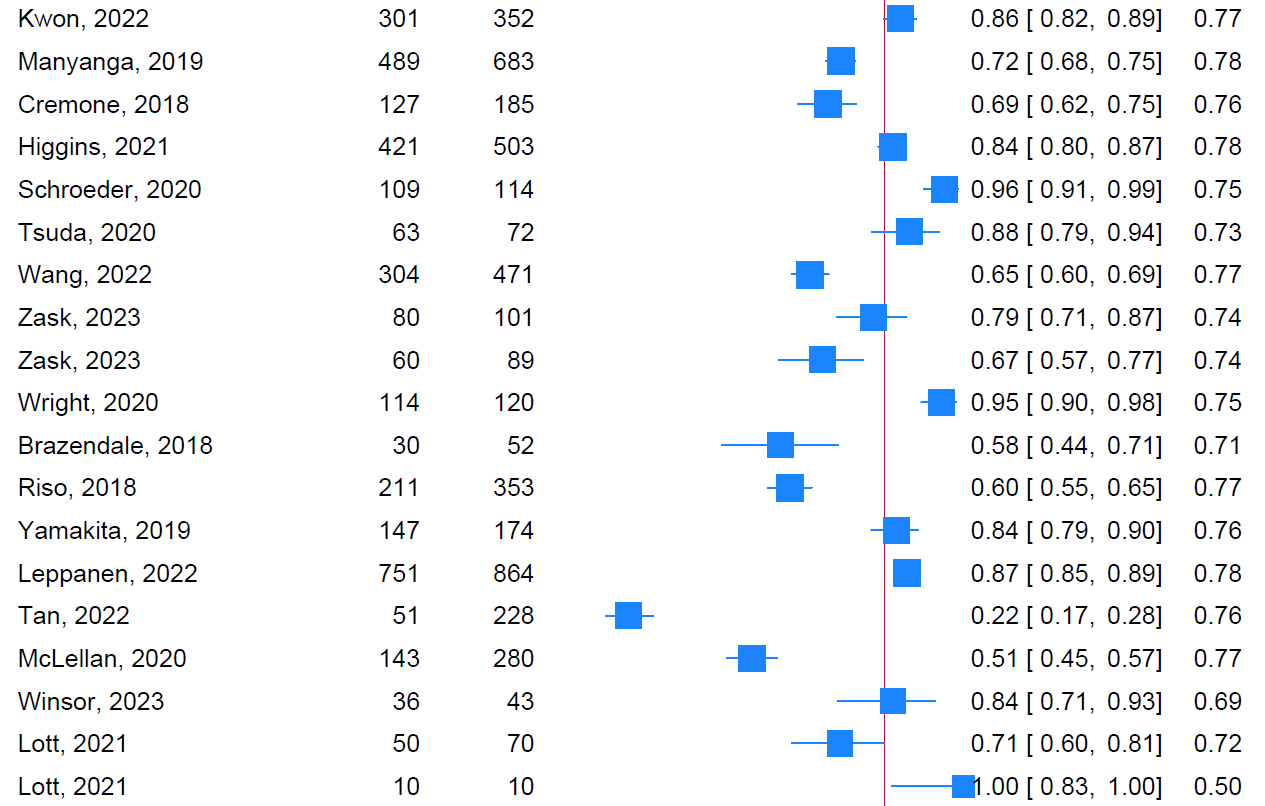


**
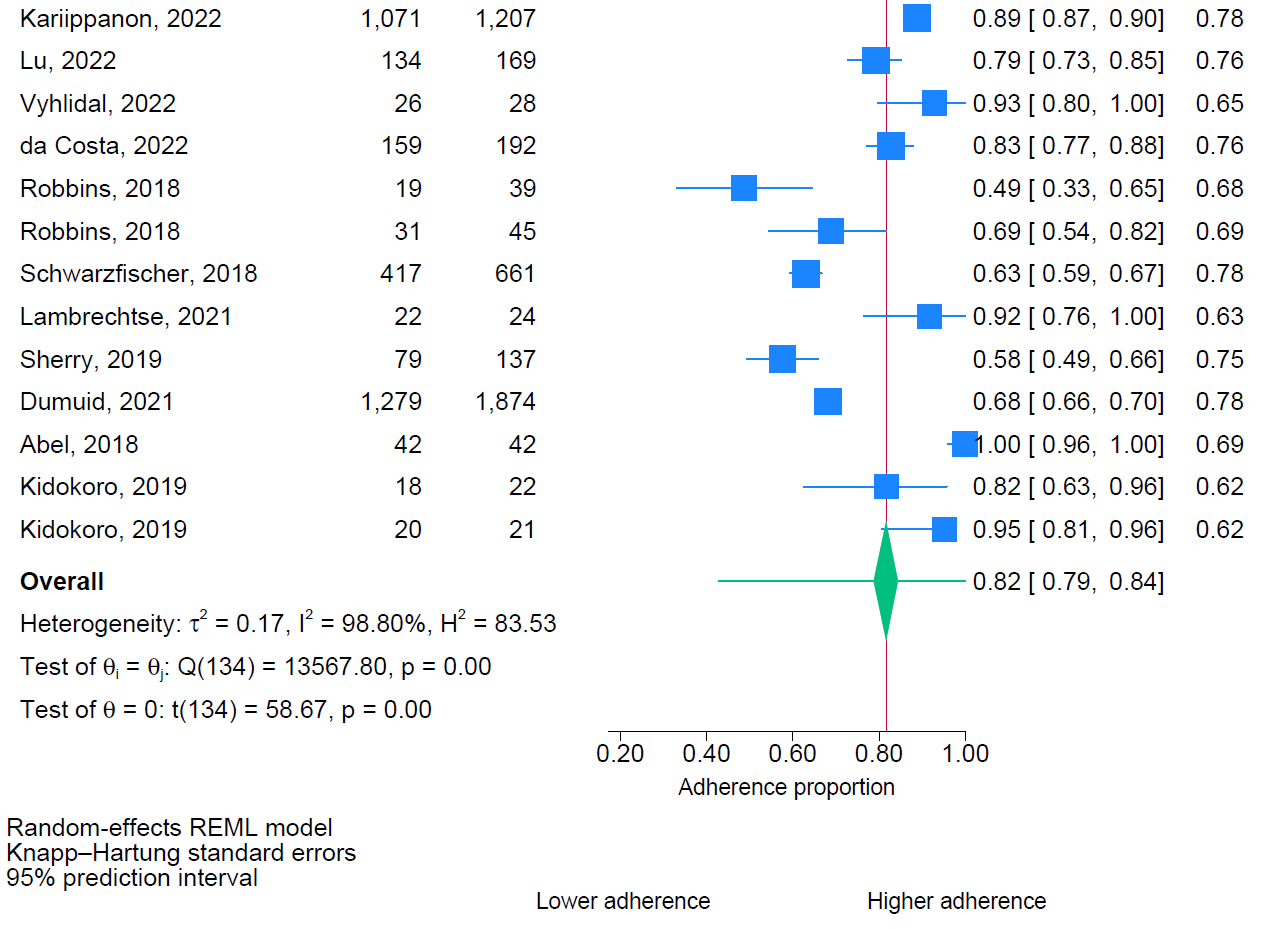
**

**Legend:** Square markers indicate prevalence estimates for individual studies, additionally the marker size is proportional to the weighting of the study in the meta-analysis. Error bars show 95% prediction intervals. The diamond data marker indicated the likely range for the pooled acitgraphic adherence across studies.
