# Supplementary material for: Adherence to Actigraphic Devices in Elementary School–Aged Children: Systematic Review and Meta-Analysis
Source: J Med Internet Res. 2025 Nov 3;27:e79718. doi: 10.2196/79718 (PMC12582557; doi:10.2196/79718)
Supplement: Multimedia Appendix 11 [file jmir-v27-e79718-s011.docx]

**Multimedia appendix 11. Full list of members of the CAMHS Digital Lab team**

Johnny, Downs, Alice Wickersham, Will Bennett, Zoe Firth, Craig Colling, Jess Penhallow, Stephen Douch, Jonathan Hind, Anna Morris, Sophie Epstein, Stephanie Lewis, Sarjhana Ragunathan Brindha, Shuo Zhang, Laurence Telesia, David Howard, Senta Haeussler, Brian Ching, Nicholas Cummins, Judith Dineley, Asilay Seker, Isabel Yorke, Akash Roy Choudhury, Lukasz Zalewski, Rosemary Sedgwick, Jane Anderson, Garry Moriarty, Daniel Smith, Sam Harris, Cato Zantman, Su Mon Latt, Hissah Al Abdulsalam, Nessma Abdelhafez, Po-Chang Tseng.
