# Supplementary material for: Adherence to Actigraphic Devices in Elementary School–Aged Children: Systematic Review and Meta-Analysis
Source: J Med Internet Res. 2025 Nov 3;27:e79718. doi: 10.2196/79718 (PMC12582557; doi:10.2196/79718)
Supplement: Checklist 2 [file jmir-v27-e79718-s013.docx]

**Checklist 2. Meta-analyses Of Observational Studies in Epidemiology (MOOSE) checklist**

| **Item No** | **Recommendation** | **Reported on Page No** |
| --- | --- | --- |
| Reporting of background should include | | |
| 1 | Problem definition | Pg 4-5 |
| 2 | Hypothesis statement | N/A |
| 3 | Description of study outcome(s) | Pg 5 |
| 4 | Type of exposure or intervention used | Pg 5 |
| 5 | Type of study designs used | Appendix 5 |
| 6 | Study population | Pg 5 |
| Reporting of search strategy should include | | |
| 7 | Qualifications of searchers (eg, librarians and investigators) | Pg 1 |
| 8 | Search strategy, including time period included in the synthesis and key words | Pg 5-6 and Appendix 2 |
| 9 | Effort to include all available studies, including contact with authors | N/A |
| 10 | Databases and registries searched | Appendix 2 |
| 11 | Search software used, name and version, including special features used (eg, explosion) | Appendix 2 |
| 12 | Use of hand searching (eg, reference lists of obtained articles) | Pg 10 (Fig. 1) |
| 13 | List of citations located and those excluded, including justification | Appendix 4 |
| 14 | Method of addressing articles published in languages other than English | Appendix 2 |
| 15 | Method of handling abstracts and unpublished studies | Appendix 2 |
| 16 | Description of any contact with authors | N/A |
| Reporting of methods should include | | |
| 17 | Description of relevance or appropriateness of studies assembled for assessing the hypothesis to be tested | Pg 5-7 |
| 18 | Rationale for the selection and coding of data (eg, sound clinical principles or convenience) | Pg 6-7 |
| 19 | Documentation of how data were classified and coded (eg, multiple raters, blinding and interrater reliability) | Pg 6 |
| 20 | Assessment of confounding (eg, comparability of cases and controls in studies where appropriate) | N/A |
| 21 | Assessment of study quality, including blinding of quality assessors, stratification or regression on possible predictors of study results | Pg 6-7 |
| 22 | Assessment of heterogeneity | Pg 8-9 |
| 23 | Description of statistical methods (eg, complete description of fixed or random effects models, justification of whether the chosen models account for predictors of study results, dose-response models, or cumulative meta-analysis) in sufficient detail to be replicated | Pg 8-9 |
| 24 | Provision of appropriate tables and graphics | Pg 8-9 |
| Reporting of results should include | | |
| 25 | Graphic summarizing individual study estimates and overall estimate | Appendix 10 |
| 26 | Table giving descriptive information for each study included | Appendix 5 |
| 27 | Results of sensitivity testing (eg, subgroup analysis) | N/A |
| 28 | Indication of statistical uncertainty of findings | Pg 15-17 |
